# Biodegradation of Biodegradable Polymers in Mesophilic Aerobic Environments

**DOI:** 10.3390/ijms232012165

**Published:** 2022-10-12

**Authors:** Anibal Bher, Pooja C. Mayekar, Rafael A. Auras, Carlos E. Schvezov

**Affiliations:** 1 School of Packaging, Michigan State University, East Lansing, MI 48824, USA; 2 Instituto de Materiales de Misiones, CONICET-UNaM, Posadas 3300, Misiones, Argentina

**Keywords:** plastics, degradation mechanisms, microorganisms, hydrolysis, biofilm, enzymes, depolymerization

## Abstract

Finding alternatives to diminish plastic pollution has become one of the main challenges of modern life. A few alternatives have gained potential for a shift toward a more circular and sustainable relationship with plastics. Biodegradable polymers derived from bio- and fossil-based sources have emerged as one feasible alternative to overcome inconveniences associated with the use and disposal of non-biodegradable polymers. The biodegradation process depends on the environment’s factors, microorganisms and associated enzymes, and the polymer properties, resulting in a plethora of parameters that create a complex process whereby biodegradation times and rates can vary immensely. This review aims to provide a background and a comprehensive, systematic, and critical overview of this complex process with a special focus on the mesophilic range. Activity toward depolymerization by extracellular enzymes, biofilm effect on the dynamic of the degradation process, CO_2_ evolution evaluating the extent of biodegradation, and metabolic pathways are discussed. Remarks and perspectives for potential future research are provided with a focus on the current knowledge gaps if the goal is to minimize the persistence of plastics across environments. Innovative approaches such as the addition of specific compounds to trigger depolymerization under particular conditions, biostimulation, bioaugmentation, and the addition of natural and/or modified enzymes are state-of-the-art methods that need faster development. Furthermore, methods must be connected to standards and techniques that fully track the biodegradation process. More transdisciplinary research within areas of polymer chemistry/processing and microbiology/biochemistry is needed.

## 1. Introduction

Plastics are pervasive and have become an indispensable part of our everyday life. The nature of plastics and their easy processability, durability, low cost, and availability favor their use, opening up an array of opportunities in market segments such as consumer goods, food and medical packaging, agriculture sector, construction, and automotive parts [1,2]. Between 1950 and 2020, global plastic production reached an accumulated amount of c. 9500 million metric tons [1,3]—estimations were obtained from references [1,3]. Results are based on production estimated from reference [3] until 2015 and the addition of production for the 2016–2020 period from reference [1]. With annual production of c. 370 million metric tons in 2020, estimates for 2030 are c. 600 million metric tons - estimation was obtained based on a linear projection growth rate from 2006 to 2018 from each global region from references [1,3] and extrapolated to 2030). However, the ability of plastics to persist, even in harsh environments, has led to white pollution (i.e., leakage and accumulation of plastics in the environment). Single-use plastics (SUPs) have been blamed as one of the main offenders of white pollution and are a growing concern for our modern society since increasing amounts end up in landfills as a portion of municipal solid waste (MSW), as litter on land, and in drainage systems, ultimately leaking into rivers and oceans [4,5,6]. At present, c. 8 million metric tons of plastic end up in our oceans annually, in addition to the 150 million metric tons that are already circulating in marine environments since the dawn of the plastic era [7,8]. A recent prediction reported that if business continues as usual without mitigation measures, c. 90 million metric tons of plastic waste will reach the world’s aquatic environments by 2030 [9].

Plastics ending up in the environment mostly start as macromolecular structures and then break down into smaller fragments called microplastics and can even be reduced to nanoplastics. Microplastics are a concern due to their ability to concentrate contaminants and become a channel for bioaccumulation, while nanoplastics are also a health concern since they can potentially translocate in cell membranes of living organisms and become a source for transporting toxic chemicals [10,11,12].

Most of the plastic waste in the ocean comes from land-based sources, such as agricultural soils, open dumps, and industries, or mismanaged plastic waste from land litter and incomplete collection, finding its way through river pathways and leading to global marine pollution [8,13,14]. Apart from rivers [13], wind and snow have also been identified as responsible for transporting airborne plastic debris to locations perceived uninhabitable and remote such as the polar regions and the French and Swiss Alps [15,16]. So, plastic pollution has called attention worldwide in the form of a global crisis leading to ecological imbalance [4,17].

A consumer paradigm shift is occurring due to the growing amount of unmanaged disposal of flexible SUPs, pushing industries to embrace the long-term circular economy of plastics [18,19,20]. As part of this circular economy, new challenges have been highlighted, such as *novel policies* targeting responsible consumption, a push for *worldwide waste management infrastructure* creation to recover plastics, and the development and production of *highly recyclable or biodegradable* plastics with a low environmental footprint (EFP) [21,22,23].

Novel policies targeting responsible consumption have been developed, such as the 2030 Agenda for Sustainable Development by the United Nations establishing the seventeen Sustainable Development Goals (SDGs) to achieve a better and more sustainable future for all [24]. Specifically, Goal 12 stipulates sustainable consumption and production, which has been adopted by countries around the world to create novel policies about the use of materials such as plastics [25]. In this sense, various U.S. states have established “extended producer responsibility” for packaging and have banned plastic bags [26,27,28]. Furthermore, bans or extra fees for some SUPs are already effective in the European Union and countries in Asia such as China and Indonesia [29,30,31], and they are in development in New Zealand and Australia [32].

The need for worldwide waste management infrastructure has been noted. In 2016, the world generated c. 2 billion metric tons of MSW and is expected to generate c. 2.6 billion metric tons of waste by 2030 if no measures are taken to curb the growing generation of waste [33]. Concentrated efforts are being directed to improve material recovery facilities around the world, with special emphasis on the lower-middle and low-income economies [34,35].

To address plastic pollution, cradle approaches related to the production of highly recyclable and biodegradable polymers with low EFP are increasingly being considered [36,37]. Replacing fossil-based plastics with bio-based plastics is one strategy to reduce the greenhouse gases (GHG) emission produced by plastics [38,39,40]. The production of biodegradable polymers is also a promising solution, primarily since they can be treated by traditional waste management options, including mechanical and chemical recycling, energy recovery and the additional route of disposal of aerobic industrial and home composting or anaerobic digestion. If enough volume of isotropic biodegradable polymers is collected and treated, they can also be commercially recycled. The efficacy of the biodegradation of these polymers is conditioned by drastically different environmental conditions, such as heat, humidity, and acidic or alkaline media, and by the polymer characteristics, such as chemical structure and physical properties.

Previous reviews on the biodegradation of polymers have focused on biodegradable polymers in general [41,42,43], biodegradable polyesters [44,45], and mechanisms of degradation [46,47]. Furthermore, recent works have reviewed and identified gaps and research needs in this area [48,49]. This comprehensive review expands on those previous works providing an overview and insights into the mechanisms, environments, and factors affecting the biodegradation of biodegradable polymers, giving special attention to the mesophilic range (20 to 45 °C). The specific goals of the review are to provide a transdisciplinary background on the aspects affecting the biodegradation of biodegradable polymers, to describe the different methods used for assessing biodegradation, and to provide insights on the degradation pathway followed by polymers susceptible to biodegradation with a focus on mesophilic conditions.

The review is organized as follows: a general discussion of the overall aspects to consider for understanding the biodegradation of polymers; a description of norms and methodologies to assess biodegradation; a discussion of microorganisms and polymers susceptible to biodegradation; and final remarks and future perspective for conducting future research.

## 2. Bio- and Fossil-Based Biodegradable Polymer Classification

**Figure 1:**
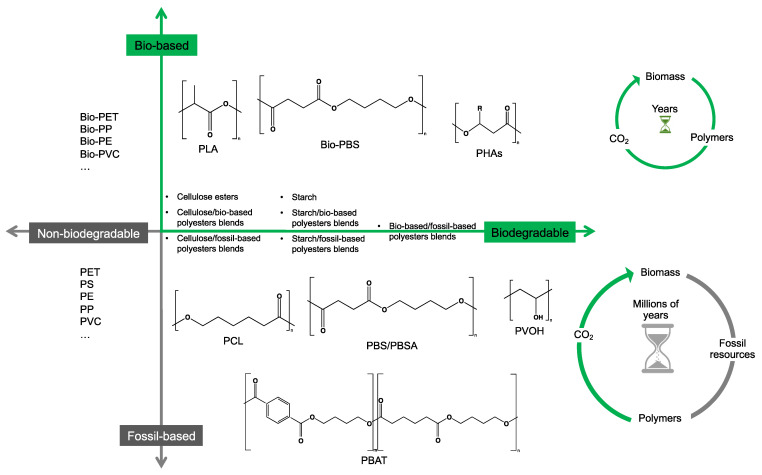
Classification of polymers considering their bio-based or fossil-based feedstock and condition of biodegradability or non-biodegradability in environments such as compost, soil, and aquatic media. Plastics can be biodegradable (right half of the quadrant) or non-biodegradable (left half of the quadrant) irrespective of their carbon feedstock. The carbon feedstock of plastics can be bio-based (upper half of the quadrant) or fossil-based (lower half of the quadrant). The relative carbon rate of bio-based and fossil-based polymers are shown on the left. PBAT, poly(butylene adipate-*co*-terephthalate); PBS, poly(butylene succinate); PBSA, poly(butylene succinate adipate); PCL, poly(caprolactone); PE, poly(ethylene); PET, poly(ethylene terephthalate); PHAs, poly(hydroxyalkanoates); PLA, poly(lactic acid); PP, poly(propylene); PS, poly(styrene); PVC, poly(vinyl chloride); PVOH, poly(vinyl alcohol). Adapted from [52,53,54].

Figure 1 provides a general classification of polymers according to their feedstock source and their ability to experience biodegradation. The first group of polymers is bio-based in nature and non-biodegradable, such as bio-based poly(ethylene terephthalate) (Bio-PET), bio-based poly(propylene) (Bio-PP), bio-based poly(ethylene) (Bio-PE) and bio-based poly(vinyl chloride) (Bio-PVC). The second group of polymers is bio-based and biodegradable, such as poly(lactic acid) (PLA), poly(hydroxyalkanoates) (PHAs), cellulose, and starch. The third group includes polymers that are derived from fossil-based sources but also present biodegradable characteristics, such as poly(butylene adipate-*co*-terephthalate) (PBAT), poly(butylene succinate) (PBS), poly(butylene succinate adipate) (PBSA), poly(caprolactone) (PCL), and poly(vinyl alcohol) (PVOH). The fourth group corresponds to the conventional group of polymers that are derived from fossil-based sources and are non-biodegradable, such as PET, polystyrene (PS), PE, PP, and PVC. This classification is very general since the characteristics of the material, the environment, and the rate of biodegradation for polymers vary widely among these groups.

As shown in Figure 1, biodegradability in regular environmental conditions is not related to the source of the polymer; however, factors such as its chemical structure and physical properties are essential [50]. Some bio-based polymers, such as bio-PE and bio-PET, are difficult to degrade as their fossil-based counterparts (i.e., PE and PET). Due to its chemical structure, as in PE, PP, PS, and PVC, the carbon-carbon backbone creates resistance to microbial degradation, and the absence of ester groups does not allow for abiotic hydrolysis but only degradation by oxidation, leaving other mechanisms, such as photooxidation that requires a timescale of the order of decades to centuries to break the backbone. However, some fossil-based polymers, such as PBS and PBAT, are biodegradable as some bio-based polymers, such as bio-PBS and PHAs, when assessed under standard conditions [51,52]. Under this classification, the prefix “bio” has been misused in the literature to refer to bio-based origin and/or biodegradable capabilities, creating much confusion, such as in the case of the term bioplastic, which has been used to refer to bio-based sources or biodegradable without specificity. Therefore, we are avoiding the use of the term bioplastic in this review. Instead, this work will address the two main groups of the classification: bio-based and fossil-based biodegradable polymers.

Considering the carbon used to produce polymers, the main benefits of biodegradable polymers can be obtained when the polymers are produced from renewable resources since they can restock the carbon cycle (i.e., the times needed to produce them and to convert them to biomass are equivalent) (Figure 1). Fossil-based polymers can also be considered renewable such as the bio-based polymers, but the main difference between both is the amount of time needed to convert to biomass and then back to their original form. Biodegradable polymers produced from bio-based resources take far less time to be converted to biomass, whereas the fossil-based polymers take millions of years to achieve the same. The longer time frames are due to the imbalance between the rate of consumption and the replenishment rate, which further leads to mass imbalance in the carbon cycle. There is no additional carbon footprint associated with renewable-carbon feedstock used to produce biodegradable polymers, such as starch-heavy crops not intended for human consumption, due to quite similar time frames for consumption and conversion to biomass [40,54,55].

## 3. Abiotic and Biotic Polymer Degradation Mechanisms

**Figure 2:**
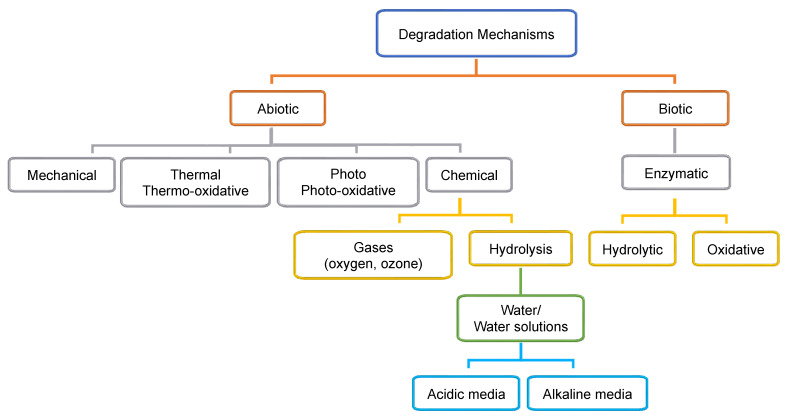
Main abiotic and biotic mechanisms of polymer degradation. Different mechanisms are involved in polymer degradation. The abiotic mechanisms can be classified as mechanical (action of environmental stressors), thermal (prolonged exposure to high temperatures), photo (action of radiation), and chemical degradation (gases and hydrolysis by water). The biotic mechanism involves the action of microorganisms (enzymatic process), which are present in each environment. One or more mechanisms can act simultaneously to bring significant changes in the polymer structure.

Polymer degradation is defined as an irreversible change of the chemical structure, physical properties, and visual appearance due to the chemical cleavage of the polymer’s constitutive macromolecules by one or more mechanism [43]. More than one mechanism can simultaneously take place due to the action of external factors, and one mechanism can be more dominant than others at any time [42]. External factors associated with the environment, such as heat, humidity, radiation, and acidic or alkaline conditions, could modify the degradation process and its rate. The degradation process can alter polymer properties such as mechanical, optical, electrical, discoloration, phase separation or delamination, erosion, cracking, and crazing [43]. The four main abiotic mechanisms associated with polymer degradation are mechanical, thermal (or thermo-oxidative), photo (photo-oxidative), and hydrolytic (chemical) degradation, some of which can be assisted by catalysis. In addition, ozone degradation (chemical) is considered a mechanism of degradation for polymers but is less common. The biotic degradation involves the action of microorganisms by enzymatic action (Figure 2).

### 3.1. Mechanical Degradation

Mechanical degradation is the loss of mechanical properties reflected in the polymer’s performance due to the exposure to either a harsh environment or the action of mechanical stresses. Mechanical degradation can occur due to compression, tension, and/or shear forces applied to a polymer. Mechanical factors are not generally predominant during the biodegradation process, but mechanical damage may happen before the action of microorganisms in activating or accelerating the biodegradation process [46]. Mechanical degradation due to loading in service is common for polymeric materials under mechanical stress, such as for biomaterials in the medical field [56]. On the other hand, physical forces, such as heating, cooling, wetting, and drying, or surface turbulence induced by air or water, can cause mechanical degradation due to stress cracking [42]. Mechanical degradation and biotic degradation are correlated, for example, when evaluating the degradation process of mulch films in agriculture settings and compostable films in industrial conditions [57]. In the scientific literature, it is common to find reports of loss of mechanical properties as an indicator of the ultimate biodegradation process, although these may not be the suitable properties to track for biodegradation but are instead complementary. The diminishing of tensile properties, flexural properties, hardness, and impact resistance are the main outcomes of mechanical degradation [46,47,58].

Evaluation of mechanical degradation in biodegradable polymers for agricultural films showed that fragmentation increased the biodegradation rate since it increased the surface area available for microbial degradation [57,59]. Furthermore, the presence of cracks and pores is typical evidence of mechanical degradation. The formation of cavities during mechanical degradation can allow for more water diffusion into the polymer matrix, affecting the hydrolytic abiotic degradation and consequently, the biodegradation process [60]. In the aquatic environment, such as marine, rivers or lakes, the stress due to the water’s natural dynamic can induce mechanical degradation of biodegradable polymers, as observed for PCL, PHAs, and PLA [61].

### 3.2. Thermal Degradation

Thermal degradation is the consequence of exposing a polymer to heat for an extended period and is called thermo-oxidative degradation in the presence of oxygen (O_2_). The first step of thermal degradation is the rupture of macromolecular bonds, resulting in monomeric units or radicals that can react with O_2_ to produce peroxide radicals [47].

For different levels of thermal energy and time exposure, thermal degradation induces different changes in the polymer structure: (1) for temperatures below the glass transition temperature (*T_g_*), thermal degradation results in physical aging, where the polymer shows a structural rearrangement; (2) for temperatures between *T_g_* and the melting temperature *(T_m_*), changes are associated with the loss of dimensions and original shape, crystallization processes and thermal decomposition of low molecular weight (*M_w_*) additives; (3) for temperatures above *T_m_*, loss of structure and disordered melt is observed due to loss of structure of the crystalline region; and (4) for temperatures even higher than the decomposition temperature, the material combusts and energy from the material can be recovered [58].

Thermal degradation occurs throughout the bulk of the polymer and consists of four different reactions that can occur at the same time: (1) chain-end scission or chain depolymerization of C-C bonds that generate volatile products; (2) random chain scission that leads to *M_w_* reduction; (3) degradation by substituent reactions; and (4) recombination reactions of cyclic and linear oligomers such as in the case of PLA [47,62].

Thermal degradation is the predominant mechanism at elevated temperatures, since its rate is higher than the rates of hydrolysis, photodegradation, and mechanical degradation. However, at temperatures lower than *T_g_*, it can induce aging of the polymer, improving the efficiency of the biodegradation process.

For biodegradable polymers, thermal degradation happens in the range of the melting temperature, which includes temperatures far higher than the range where the biodegradation process mostly occurs (i.e., at mesophilic and thermophilic conditions, 20–60 °C). The *T_m_* is around 155 °C for PLA and 175 °C for poly(hydroxy butyrate) (PHB), indicating that the thermal degradation will not affect or accelerate the biodegradation process. However, for some thermoplastic polymers such as PCL, the *T_m_* is around 60 °C, close to the thermophilic range of the composting process so that thermal degradation can play an active role during the biodegradation process [46]. The energy provided can introduce modifications in the macromolecular structure and enhance the biodegradation process due to increased polymeric chains’ mobility, rearrangement, and the creation of free volume [46].

### 3.3. Photodegradation

Polymers can undergo photodegradation and radiation degradation when exposed to wavelengths in the UV, visible, and infrared (IR) spectrum range or gamma radiation. Photodegradation may occur in the absence of O_2_ (photolysis) and the presence of O_2_ (photooxidative degradation), leading to rearrangement, chain scission, and cross-linking. The degree of photodegradation is associated with the wavelengths found in sunlight: infrared (IR) radiation, visible light, and UV radiation. The radiation reaching the earth’s surface is in the wavelength of 295 to 2500 nm, corresponding from UV-C to IR [63].

Polymers that absorb high energy in the UV range are susceptible to oxidation and cleavage due to electron activation at higher energies [43,64]. Photodegradation can break the polymer chains, produce radicals, change the physical and optical properties, generate a yellowing effect, induce loss of mechanical properties, and reduce the *M_w_*, leading to a useless material [47,65]. Photooxidative degradation in polymers can be induced by UV radiation with or without the action of a catalyst, and the increase in temperature can accelerate the process.

In photolysis, light absorption leads directly to the formation of chemical reactions that cause degradation. For polyesters and polyamides, the photolysis mechanism implies two photolytic reactions, Norrish I and Norrish II [66].

In semicrystalline polymers, the scission is mostly produced in the amorphous fraction and generates two end chains that can restructure and increase crystallinity as degradation continues. The termination step of photooxidative degradation collects free radicals to create inert products. The combination of free radicals can be natural or assisted using stabilizers in the polymer. A review of this process can be found elsewhere [47,65,67].

Photodegradation can lead to Norrish reactions, and/or crosslinking reactions, or oxidative reactions. The products of the Norrish reactions transform the polymer by photoionization (Norrish I) and chain scission (Norrish II) [58]. Studies on poly(l-lactide) (PLLA) and PCL have shown that photodegradation followed a Norrish II reaction [68,69]. Furthermore, crosslinking was observed for PBAT [70]; when PBAT films were exposed to solar radiation, the loss of integrity and mechanical degradation observed was due to chain scission and crosslinking [70,71].

On one hand, photodegradation can induce chain scission that can contribute to the biodegradation process. On the other hand, photodegradation can induce crosslinking, limiting the mobility of polymer chains and the access of water into the bulk’s polymer, reducing the activity of microorganisms, decreasing the rate of the biodegradation process. Enzymatic degradation of PLLA has been reported to be affected by UV treatment due to a dual effect of C=C double bonds formation and reduction in *M_w_* that also affected the chemical hydrolysis of PLLA films [69]. A similar effect was reported by Jeon and Kim [72], where for a short UV treatment, the initial *M_w_* was the dominant effect. However, at higher times of treatment, the crosslinking was probably dominant and reduced the biodegradation of PLA.

### 3.4. Ozone Degradation

The effect of atmospheric ozone on polymers is an increase in the aging rate, leading to a reduction in *M_w_* and loss of performance in mechanical and O_2_ barrier properties [73].

Poly(vinyl alcohol) (PVOH) has been shown to be degraded by the action of ozone. The associated mechanism starts with the oxidation of the -CHOH- group, which leads to ketonic groups. Fourier transform infrared (FTIR) spectroscopy has shown that the final product is a PVOH oligomer with several ketonic groups along the main oligomer backbone and carboxylic end groups [74]. Abiotic degradation by the action of ozone, as reported by Cataldo et al., resulted in a loss of original PVOH crystallinity, accelerating the biotic degradation process [74]. However, for PLA, an increase in crystallinity was reported by Olewnik-Kruszkowska et al., and the amorphous region was the most affected during ozone degradation [75]. Furthermore, changes in the polymer matrix surface due to ozone exposure were observed as an increase in the surface roughness [75,76]. Roughness can be beneficial for biofilm formation during biotic degradation. Overall, ozone degradation affects the bulk and surface structural properties of the polymer as observed for crystallinity and surface roughness, and consequently, it affects the biodegradation rate.

### 3.5. Hydrolytic Degradation

Chemical hydrolytic degradation is one of the main abiotic degradation mechanisms for biodegradable polymers, especially for aliphatic and aliphatic/aromatic polyesters. In this review, we refer to this mechanism also as chemical hydrolysis.

With the uptake of water, susceptible chemical bonds in polymers can undergo chain scission, resulting in a reduction in *M_w_*, loss of mass and mechanical properties, and increased surface area of the polymer, thereby increasing the available sites for attack by enzymatic activity, which is the biotic step initiated by microorganisms [42,46,77].

Chemical hydrolysis proceeds via two mechanisms when considering the macrostructure: bulk and surface erosion. Depending on the conditions, these mechanisms can occur independently or combined. Bulk erosion is the dominant mechanism when the water diffusion is faster than the hydrolysis reaction rate, and surface erosion is dominant when the water diffusion into the polymer bulk is slower than the hydrolysis reaction rate [42,44,56].

When bulk erosion is the dominant mechanism, the *M_w_* of the polymer is reduced so that the polymer loses its mechanical properties in a short period. Due to the *M_w_* reduction and higher mobility of shorter polymeric chain segments, crystallinity may change. Loss of mass and changes in geometric shape take more time. The by-products of bulk erosion are first accumulated; when the polymer chains are short enough and reaching *n*-mers size, they can start to diffuse out. When the polymer undergoes surface erosion, the mass loss is mostly from the surface while the bulk remains intact. As degradation advances, mass loss happens faster at the surface, and the polymer reduces in size. When compared with bulk erosion, the mechanical properties and *M_w_* are preserved for an extended period, and the release of by-products from the surface occurs from the beginning [56].

The kinetic rate of the chemical hydrolysis—surface or bulk dominant—depends on and can be affected by several factors associated with the polymer itself and the environment. The roles of some factors are discussed in the next sections, and additional information can be found elsewhere [42,44,56,78].

In terms of environmental factors, an increase in temperature and moisture intensifies the rate of chemical hydrolysis [79]. Polymer chain mobility increases as the temperature increases. Hence, the susceptibility of hydrolysable bonds to undergo chain scission increases. The chemical potential of water on the surrounding media plays a significant role in the hydrolysis of polymers [80]. Hydrolysis in acidic or alkaline conditions can occur through different mechanisms so the by-products of the reactions can differ [81]. Finally, catalysts can increase the rate of the hydrolytic process [82,83]. In terms of polymer factors, hydrophilic polymers are more susceptible to hydrolytic degradation than hydrophobic polymers [42].

Hydrolysis depends on the presence of hydrolyzable covalent bonds, such as esters, ethers, anhydrides, carbamide (urea), and ester amide (urethane), which increase the rate of chemical hydrolysis [46,84]. Table 1 compares the half-lives of hydrolyzable bonds in various polymers and shows that poly(anhydride)s are subjected to rapid hydrolysis due to the presence of hydrolyzable bonds of very low half-life. By contrast, polyamides are resistant to hydrolysis due to the resistance of the amide bonds to hydrolysis. The kinetics of the hydrolyzable bond half-life presented in Table 1 can increase or decrease due to the influence of neighboring groups.

The presence of amorphous regions increases the chemical hydrolysis rate due to the easy diffusion of water into the polymer matrix compared to semi- and crystalline polymers, showing well-organized structures where diffusion is limited, even at temperatures higher than *T_g_* [86,87,88]. So, for polymers with lower or similar values of *T_g_* than the mesophilic range, the diffusion is mostly controlled by the amorphous region where chemical hydrolysis is dominant.

The macro structure properties, such as the size and shape of the polymer, are factors that condition whether the dominant mechanism will be either surface or bulk erosion. In this way, a material can go from surface to bulk erosion when its thickness is reduced to a value lower than a critical value, which is called the critical sample thickness (*L_crit_*) [78,89].

Polyesters, due to the presence of ester groups, are degraded by chemical hydrolysis. Bulk degradation is predominant for aliphatic polyesters, such as poly(glycolic acid) (PGA), PLA, PCL, and PBS. The main stages of the hydrolytic degradation of polyesters undergoing bulk erosion can be summarized as (1) diffusion of water in the polymer matrix (amorphous regions); (2) water reacting with random ester linkage to produce shorter chains; (3) autocatalysis due to the presence of acid chain ends in the medium; and 4) release of water-soluble oligomers and monomers creating a void core and subsequent reduction in *M_w_* [42,90]. The duration of the chemical hydrolysis process depends mainly on the initial *M_w_*, crystallinity, temperature, and pH [47].

PLA is an example for chemical hydrolysable polymer degradation. In this sense, the environment to which the material is exposed and factors such as temperature, pH, and moisture play major roles in delaying or speeding up the hydrolytic degradation rate. In an industrial composting process (≈58 °C and ≈60% RH), PLA can absorb water and undergo chemical hydrolytic degradation. However, at lower temperatures, such as in agricultural soil environments (≈25 °C), the rate of chemical hydrolysis is low, increasing the time for the enzymatic hydrolysis process to start. One of the main differences between bulk and surface erosion mechanisms can be recognized in the diffusion of the degradation by-products. During the bulk degradation of polyesters, these hydrolysis-formed oligomer and monomer by-products, such as carboxylic acid and hydroxyl groups, are trapped and accumulated inside the bulk, leading to an autocatalytic degradation that tends to accelerate the degradation kinetics [44,91]. Burkersroda et al. [89] reported that the hydrolytic degradation of PLA, evaluated at 37 °C, follows a bulk erosion mechanism for thicknesses between 0.5 and 2 mm, a core-accelerated erosion for thicknesses between 2 and 74 mm, and surface erosion for thicknesses greater than 74 mm. Hoüglund et al. [92] reported that the hydrolysis of 100% PLLA increased upon the addition of a low percentage of d-Lactide units due to a reduction in the polymer order structure, showing the effect of tacticity and optical purity on the hydrolytic degradation of PLA. In comparison to PGA hydrolysis, PLA hydrolysis is delayed due to the presence of the methyl group in PLA that blocks the attack of water to interact with the hydrolysable bonds [42,56]. For more insights, a review of PLA’s hydrolysis has been reported by Tsuji [78].

PBAT, due to the presence of an aromatic group in its polyester chain (shown later in Section 5), experiences a lower hydrolytic degradation rate than polyester with only aliphatic units as PLA and PGA [93]. The presence of the aromatic group reduces chain flexibility, provides less susceptible bonds, and creates a steric interference effect to the access of the susceptible ester bonds [94]. The soft aliphatic domain bonds consisting of 1,4-butanediol and adipic acid monomers (BA) are more susceptible to hydrolysis than the hard aromatic bonds of 1,4-butanediol and terephthalic acid monomers (BT). In this sense, PBAT displays good biodegradability when the aromatic moiety concentration is kept below 55 mol% [95]. Kijchavengkul et al. [71] also demonstrated that the increase in crosslinking on PBAT has a detrimental effect not only on chemical hydrolysis but also on enzymatic hydrolysis.

Polymers that undergo surface erosion are desirable when designing surgical medical devices and for drug release, since the retention of mechanical properties and capacity for a controlled release of drugs can be achieved by mass loss without compromising the *M_w_*. Some examples are polyanhydrides, some poly(ortho esters), and some polycarbonates [96,97,98].

### 3.6. Biotic Enzymatic Degradation

**Figure 3:**
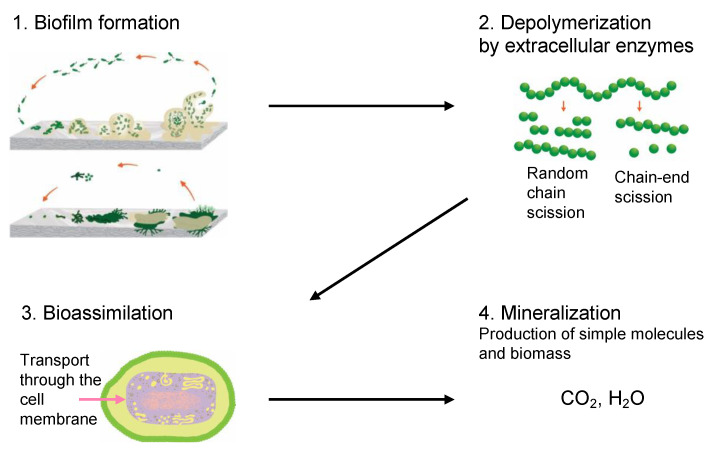
The four main stages involved in the biotic degradation process: (**1**) biofilm formation—establishment of microbial colonies on the polymer surface through the secretion of extracellular polymeric substances, (**2**) depolymerization—breakdown of polymer chains into small molecules such as oligomers, trimers, dimers, and monomers by the action of extracellular enzymes, (**3**) bioassimilation—metabolization of low *M_w_* compounds (dimers, monomers) by transportation through the cell membrane and (**4**) mineralization—carbon is biologically oxidized to CO_2_ through a series of cycles, releasing energy and water and other compounds. Adapted from [99].

Biotic enzymatic degradation is the mechanism where microorganisms break down organic substances through an enzymatic process. The four main stages of biotic degradation are shown in Figure 3. The main outcome of biotic degradation is the reduction of the polymer structure to small molecules that are assimilated by the microorganisms as a source of carbon and energy, resulting in final products such as CO_2_ and H_2_O in aerobic conditions. Microorganisms such as bacteria and fungi are actively involved in the biodegradation process. These microorganisms have their own optimal growth conditions; for this reason, biotic degradation is a complex process where several factors associated with the polymer, microorganisms, and the environment come into play [79]. The abiotic mechanisms described above, such as photo, hydrolytic, or even mechanical degradation, can enhance the biotic degradation process by increasing the surface area for biofilm formation or by reducing the *M_w_* [47]. However, the dominant mechanism in the biotic degradation process is related to biotic agents.

#### 3.6.1. Biofilm Formation

Biofilm formation has been identified as the dominant phase of life for microorganisms on Earth. Studies have shown that microorganisms, in general, live in aggregates or mixed species rather than as single cells in pure cultures [100,101]. When a biodegradation process occurs, biofilm formation is considered the first stage and a necessary one in the process. However, the formation of a biofilm on a surface does not necessarily imply that biodegradation will occur [102].

In biofilm formation, a microbial community is established on a surface. These surfaces, such as metals, sediments, or plastics, can exist in different forms, have different properties, and consist of different compositions. Biofilms are considered highly sophisticated and complex synergistic structures originated by the selective attachment of phylogenetically and functionally diverse communities of bacteria, fungi, protozoans, or algae [103]. The organization of microorganisms on a surface is specific to the material and dependent on that material’s surface properties and the environmental conditions. Biofilms can be developed in solid/liquid and solid/air interfaces [102].

**Figure 4:**
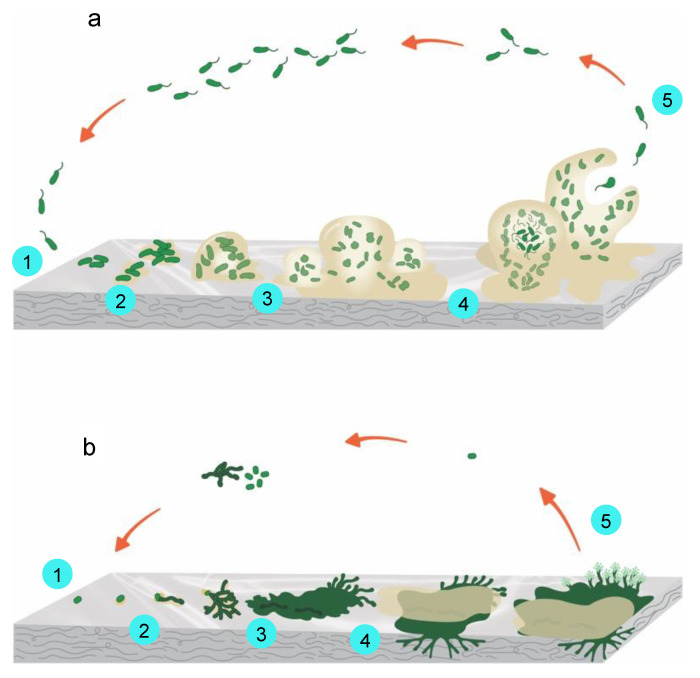
(**a**) Bacteria biofilm formation, main steps: (1) attachment of microorganisms to the surface using a specialized glue-like substance and tail-like structures, (2) colonization, (3) growth, (4) maturation, and (5) detachment; (**b**) Fungi biofilm formation, main steps: (1) attachment of microorganisms to the surface using a specialized glue-like substance and tail-like structures, (2) colonization, (3) growth, (4) maturation, and (5) detachment. Adapted from [101,106,110].

The first step of biofilm formation for bacteria is the microorganism’s initial attachment to the surface via the cell pole or the flagellum (within minutes after the first contact with the substrate) (Figure 4a). The initial attachment is a reversible step. The second step of biofilm formation is the microorganisms’ irreversible attachment to the surface using a glue-like substance and tail-like structures. The attached microorganisms start producing slimy extracellular polymeric substances (EPS), formed by proteins, polysaccharides, nucleic acids, lipids, and humic substances, and they develop clusters of cells in contact with each other and with the substrate. EPS production allows the microbial community to develop a complex structure highly influenced by environmental factors, and it is the main factor responsible for the adhesion to surfaces and the integrity of the biofilm [104]. During this second step, the growth of microbial communities can occur in a matter of hours. Biofilm maturation occurs in the third step when cell clusters embedded in the EPS become mature and layered. A high level of biofilm maturation is achieved as cell clusters, and microcolonies reach their maximum average thickness in the fourth step. In the final step, as the maturation of the colonies progresses, the complex structures weaken, detach from the substrate, release, and propagate. This variable-sized group of cells can now attach to a different zone of the surface or another previously optimally developed biofilm. The detachment step is characterized by cells evacuating from the interior of the clusters, forming void spaces [100,105].

In the case of the fungi population, the development of filamentous fungi biofilm has been proposed (Figure 4b) [106]. The first step, similar to bacteria biofilm, implies the deposition and adsorption of spores and/or hyphal fragments. The second step implies the development of a fungal EPS for active attachment to the surface. In the third step, a microcolony is formed with the branching of a monolayer hyphal and extension of the EPS for better adherence of the microcolony to the surface of the substrate. In the fourth step, a colony is formed, a hyphal compacted network is developed, and the maturation of the colony occurs. Finally, in the fifth step, the dispersal or release of new cells takes place. These new cells can start a new cycle.

A detailed discussion of the biofilm formation mechanism can be found elsewhere [107,108,109].

#### 3.6.2. Depolymerization

**Figure 5:**
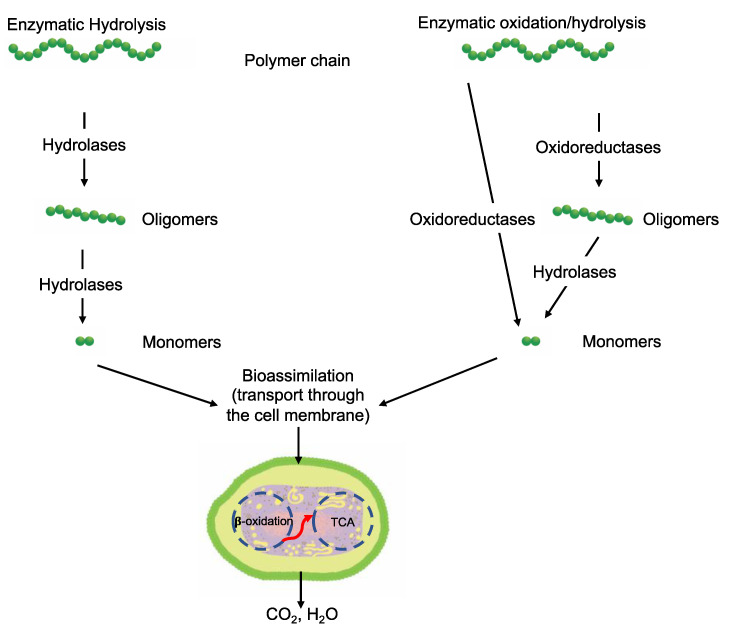
Enzymatic hydrolysis or oxidation routes for depolymerization. Enzymes specific to the polymer substrate and the environment are released by the microorganisms to initiate depolymerization of the polymer chains. The low *M_w_* fragments (oligomers and monomers) are utilized for energy production essential to its biochemical processes. Hydrolases (EC 3) and oxidoreductases (EC 1) are the main group of enzymes linked to the depolymerization of polymer chains. Adapted from [111].

The enzymatic activity that occurs after biofilm formation is the main contributor to the depolymerization stage. Enzymatic activity can occur via a hydrolytic or oxidative route (Figure 5), involving either random or end chain scission [47,79].

The oxidative mechanism is called enzymatic oxidation. In the case of non-hydrolysable polymers, due to the absence of hydrolysable groups, redox reactions are the most effective way to break the backbone made of C-C bonds. However, extracellular enzymes must have redox potentials high enough to allow the electron extraction from non-reactive C-H or C-C bonds. A high redox potential requirement could be an important obstacle for ultimate polymer biodegradation [111].

As chemical hydrolysis progresses, the *M_w_* is reduced, and consequently, the polymer becomes available for enzymatic hydrolysis, which starts dominating the depolymerization stage. For hydrolysable polymers, with ester, carbonate or amide groups, the hydrolytic enzymatic degradation by extracellular hydrolases has been reported and is presented and discussed in the next sections.

Within the major enzyme classes (Table 2), hydrolases (EC 3) and oxidoreductases (EC 1) are the main groups of enzymes linked to depolymerization.

The lower activation energy needed for the enzymatic hydrolysis of ester linkages, such as those in aliphatic and aliphatic/aromatic polyesters, appears to facilitate the depolymerization of polyesters in comparison to polyolefins, where non-hydrolysable linkages are present. However, large differences have been reported in the rates of biodegradation for polyesters as a function of their morphology and chemical structure. For example, the aromatic polyester PBT is considered non-biodegradable; however, the copolymer obtained from terephthalic acid and adipic acid, PBAT, is biodegradable. In addition to the presence of the aromatic ring in both structures as well as hydrolysable bonds, the presence of the adipic acid component in PBAT improves the flexibility of the polymer structure, making it more susceptible to the attack by extracellular enzymes [45].

Enzymes are macromolecules made up mostly of proteins, which are complex chemical structures, with high *M_w_* and hydrophilic groups acting as biocatalysts that accelerate the depolymerization reaction rates by lowering the activation energy of the reaction [113]. The simplest enzymes consist entirely of amino acids, while conjugated enzymes contain a non-protein component, a cofactor (or co-enzyme) along with a protein component.

**Figure 6:**
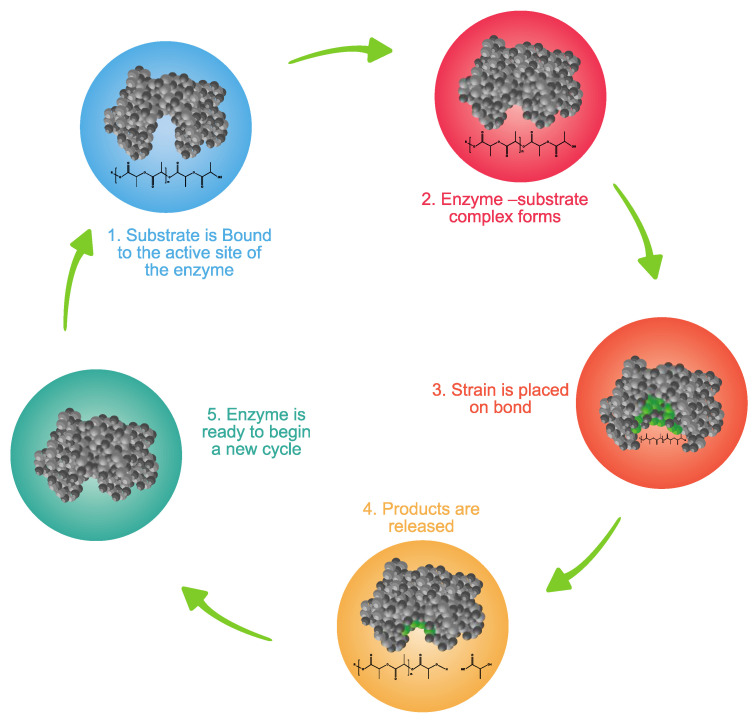
Catalytic cycle of an enzyme. Enzymes secreted by the microorganisms contain an active site to catalyze the depolymerization reaction. (1 and 2) Polymer substrate enters the active site of the enzyme and fits in a specific orientation to form intermediate enzyme–substrate complex. (3, 4, and 5) The substrate is converted to products, and the enzyme is available to take up another substrate. Adapted from [117].

Extracellular enzymes are key for the breakdown of water-soluble substrates (e.g., polysaccharides, proteins, and nucleic acids) or water-insoluble substrates (e.g., cellulose, lipids, and bio- or fossil-based polymers chains), leading to depolymerization [104,114]. Extracellular enzymes are released when optimal conditions are present between the substrate and the attached biofilm. Enzymes bind to a substrate by their active site and transform the substrate into a product. Figure 6 shows the steps of this process. First, an enzyme binds to its substrate and positions it properly in its active site to catalyze the reaction. In the second step, the enzyme–substrate complex is formed. In the third step, the enzyme–substrate complex aligns reactive groups in the substrate and places strain on specific bonds, reducing the activation energy required for making the reaction occur. In the fourth step, the cleaved products are released. Finally, in the fifth step, the enzyme is ready to begin the catalytic cycle again.

The main factors influencing the susceptibility of a polymer toward microbial attack by extracellular enzymes are:

1. *Enzyme availability*. Availability is determined by the type of microorganisms and the environment.
2. *Available sites on the polymer for enzyme attack*. Extracellular enzymes are classified as exo- and endo-enzymes. Exo-enzymes are responsible for chain end scission, while endo-enzymes are responsible for random chain scission [115].
3. *Enzyme specificity*. Enzymes are known as catalysts of biochemical reactions with high substrate specificity. This means that an enzyme catalyzes a special reaction with high efficiency. Therefore, many different reactions catalyzed by different enzymes can run in parallel simultaneously. The specificity is a function of the three-dimensional structure of the enzyme [115].
4. *Presence of cofactors.* Cofactors are additional chemical groups incorporated to the structure of the active site of the enzyme to facilitate a biochemical reaction. Cofactors can be metal ions (e.g., calcium, magnesium, potassium, sodium, or zinc) or co-enzymes (organic cofactors). A common function of cofactors is to provide a geometric place for the substrate to bind to the enzyme by maintaining the stability and activity of the enzyme at the active site [116].

The priority of extracellular enzymes is to obtain carbon to ensure the supply of resources. Additionally, the microbial community can shift enzyme production between groups of substrate-specific enzymes and non-specific enzymes to match substrate requirements. In other words, enzymes are selectively produced to increase the supply of the most limiting element and to target the most available substrates [118]. From an energy point of view, enzyme production is energy intensive. For this reason, microorganisms produce enzymes at the expense of growth and metabolism when nutrients are scarce. Furthermore, when available nutrients are scarce, microorganisms can produce adaptive enzymes to obtain resources from complex sources [119]. On the other hand, when assimilable nutrients are available and abundant, the production of constitutive enzymes may be decreased [120].

For polymer degradation, depolymerases are the extracellular enzymes secreted by microorganisms that cleave complex polymeric substrates into oligomers, dimers, and monomers. The hydrolytic cleavage can be by exo-attack or endo-attack. Exo-attacks occur at the end of the polymer chain, and the by-products are oligomers or monomers that can be assimilated by the cell. Endo-attacks occur randomly along the polymer chain, reducing the *M_w_*; hence, products are not assimilable without further depolymerization [121,122]. An important characteristic of extracellular enzymes is that they are too large to penetrate deeper into the polymer material. For this reason, enzymes can only act on the polymer surface, making depolymerization by enzymatic activity a surface erosion process [115]. Increasing the surface area can increase the rate of depolymerization by extracellular enzymes [123]. Fragments small enough to go through the membrane cells as monomers are transported inside the cell and transformed to obtain energy for the growth process by the action of intracellular enzymes. Usually, these are oxidative enzymes, and the process is called bioassimilation or assimilation.

#### 3.6.3. Bioassimilation

**Figure 7:**
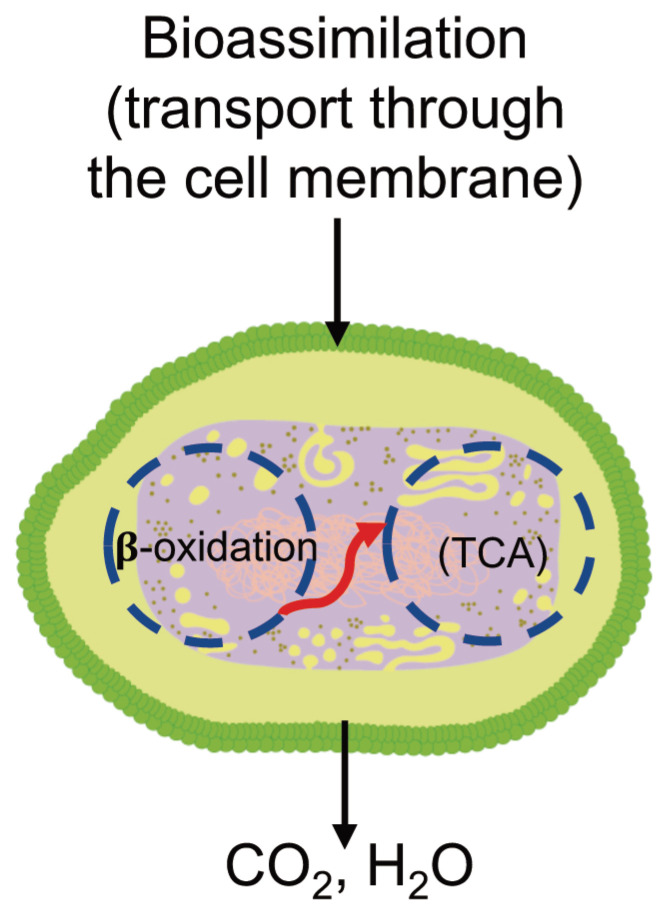
Microbial bioassimilation and mineralization during the polymer biodegradation process. The depolymerization products (oligomers, dimer, and monomers) are transported through the cytoplasmic membrane and are used as an energy source via the β-oxidation and the tricarboxylic acid (TCA) cycles thereby releasing CO_2_ and water. Adapted from [126].

Bioassimilation is related to the acquisition or uptake of substances for the microbial metabolic process. Compounds small enough to pass the semi-permeable membrane after the depolymerization stage can be potentially processed by the metabolism of the microorganism and finally mineralized (dissimilation) or be used for the biosynthesis of new products through metabolic pathways (assimilation) (Figure 7). In general, the periplasmic space—the cell membrane—is where the cleavage takes place and from where oligomers can be transported across the cytoplasmic membrane for further oxidation in the β-oxidation cycle. Oligomers can be internalized with the aid of surfactants produced by microorganisms during biofilm formation and be used as carbon and energy sources by the action of intracellular enzymes. The presence of water for the transport of components is a critical factor during the bioassimilation stage [117].

#### 3.6.4. Mineralization

Mineralization, or ultimate biodegradation, refers to the degradation of polymer fragments to the mineralized components and biomass, plus CO_2_ and H_2_O in aerobic conditions (Figure 7). Depending on the polymer composition, other compounds also can be released, including sulfide, sulfate or sulfite, ammonia, nitrite or nitrate, phosphate or phosphite, chloride, and fluoride. By measuring the mineralization levels (i.e., CO_2_ released or evolved), biodegradation rates and percentage of mineralization can be quantified. Bioassimilated monomers are part of the catabolism cycle. During this step, organic compounds, such as carbohydrates and proteins, are used as metabolites of the tricarboxylic (TCA) cycle or Krebs cycle by aerobic microorganisms to produce energy [104,124]. Insights on external factors affecting the mineralization of biodegradable polymers can be found elsewhere [125].

## 4. Biodegradation Environments

There are many feasible waste management recovery processes for polymers, ranging from recycling, biodegradation as defined by the U.S. Environmental Protection Agency (EPA), waste-to-energy conversion, and landfill, each with trade-off environmental impacts. Littering or leakage to the environment must not be considered part of the waste management process. Each of these waste management environments present specific conditions that can tailor the degradation rate of polymers. So, the evaluation of degradation of a polymer in different environments may reveal different rates due to the influence of external abiotic and biotic factors [127,128,129]. Table 3 presents a summary of the temperature ranges and the main environments where biodegradation occurs.

### 4.1. Soil Environment

Soil is a typical disposal scenario for biodegradable and non-biodegradable polymers employed as agricultural mulch films [130,131]. For several decades, non-biodegradable fossil-based polymers, such as PE, have been employed as mulch films for crops. However, in the last 15 years, bio-based and fossil-based biodegradable polymers have gained market momentum, since their use can avoid the removal of the plastic film after harvest and reduces the leakage of plastic debris [132,133].

**Figure 8:**
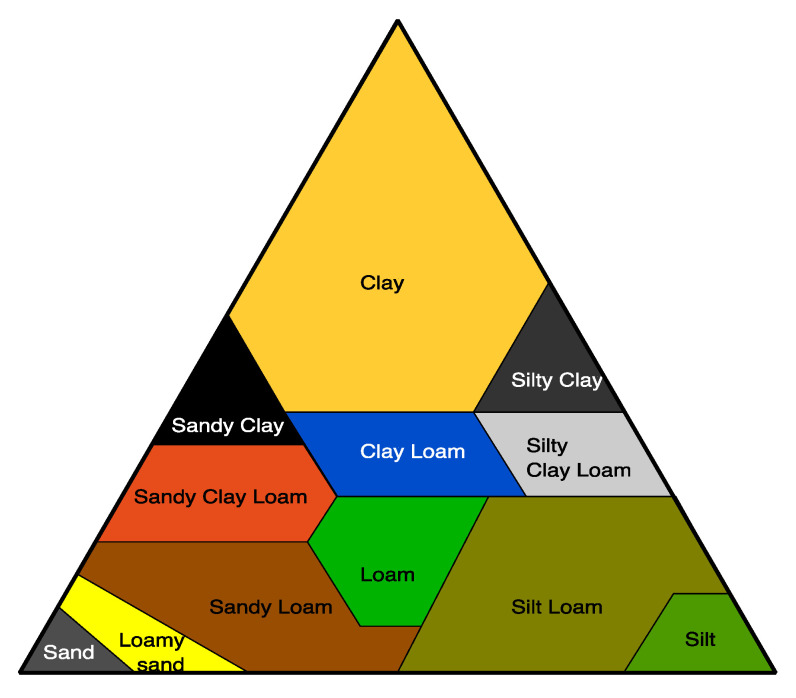
United States Department of Agriculture (USDA) soil texture classes. The USDA classifies soil into twelve major categories based on the texture (particle size) and the composition of sand, silt, and clay. Adapted from [136,138].

Soil is a diverse habitat for microorganisms [134,135], and biodegradation usually takes place in the mesophilic range of temperature. The main parameters used to classify a soil are based on its granularity and porosity due to the relative proportions of clay, sand, and silt (Figure 8). The texture and structure of soils is determined by the relative proportions of clay, sand, and silt and their relative sizes [136,137]. Silty soils can possess high water retention capacity, but clay soils possess the highest water retention capacity.

The chemical and biological properties of soils are characterized by acidic/alkaline media, cation exchange capacity, organic carbon concentration, and soil respiration [139]. These properties control the formation and activity of the microbial diversity, and the combination of the mentioned factors creates habitats where only certain microorganisms can grow [84]. The distribution of the different particles creates pores of different sizes that can retain water or surrounding living organic material. The soil connectivity determines the circulation of nutrients, soluble organic compounds, and water, and it is ultimately tied to the pore geometry and network [140]. Thus, the size of the pores is a factor that determines and helps explain the spatial separation of living organisms [134].

The biodegradation process occurring in soil environments should consider the surface layer and underground matrix [141]. The surface layer of the soil is highly affected by abiotic factors. On the other hand, the underground matrix is associated with the microbial population and factors for its optimal activity [84]. The factors playing major roles in the biodegradation process in soil are soil texture and structure, water content, organic matter, pH, temperature, O_2_, and sunlight [142].

A dry soil encourages the formation of fungal populations, while a wet soil promotes the genesis of bacterial populations [142]. Fungi spread through the soil using hyphae, which are thin filaments forming the mycorrhizal network. Under dry conditions, while in search of water and nutrients, the hyphae spread and take different routes. The fungi continue enlarging this network and bridge the gaps between different small pockets of water and nutrients, thus enabling survival and growth in soil, where the moisture content may be low [143].

Microorganisms can adapt to specific ranges of pH values. Thus, the soil pH is a factor that can limit the growth of microorganisms. Alkaline to neutral pH favors bacterial growth, whereas acid pH favors fungal development [142]. The pH influences the availability of nutrients and concentrations of trace metals such as zinc, iron, calcium, magnesium, and phosphorus. Fungi take in these molecules across their membranes by creating a proton gradient; this proton gradient affects the ability to take up the nutrients when exposed to extreme pH conditions [144]. In acidic media, certain nutrients, such as phosphorus, become less available and other nutrients such as magnesium and aluminum can become more toxic, thus creating a hostile environment for helpful soil bacteria.

The O_2_ content of the medium determines whether the microbial population expressed is aerobic or anaerobic. Soil temperature governs the physical, chemical, and biological processes in the soil. Changes in soil respiration rate due to the fluctuation in temperatures also affect the bioactivity. Microbial activity is inhibited or reduced drastically with lowering temperatures [145]. Radiation, mostly from UV light, can inhibit the growth of microbial populations, depending on the intensity of the radiation. The optimal conditions of temperature, organic matter, aeration and O_2_, and water content are in the first 30 cm of the soil layer [136,141].

**Figure 9:**
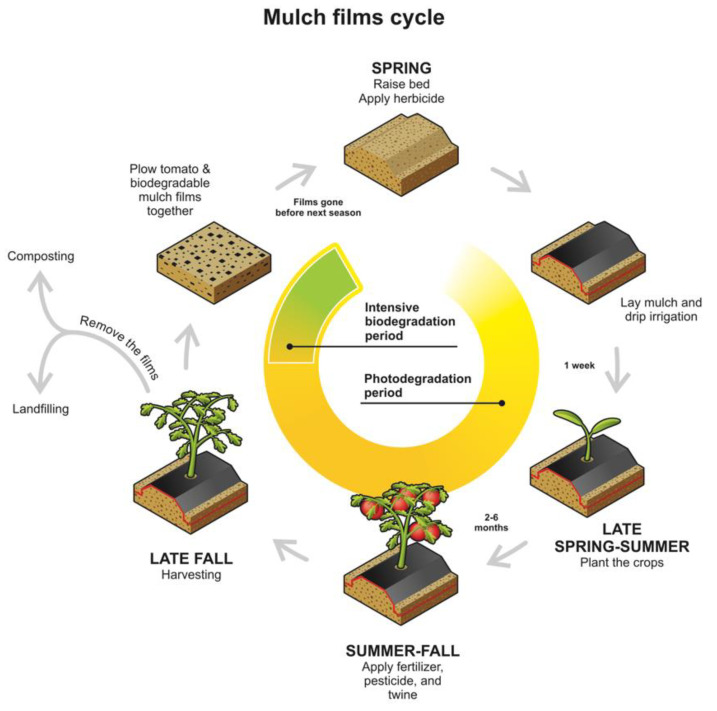
Biodegradable mulch film cycle, starting from raising the bed and applying herbicide in spring, to harvesting and the disposal of the films in late fall, and the associated degradation processes where different factors such as UV radiation, mechanical stress, temperature, and environmental weathering contribute toward the physical and chemical aging and determine the degradation of mulch film in soil [70] (Copyright 2008. Reproduced with permission from Elsevier Science Ltd.).

Agricultural soils can be considered as a particular type of soil environment, and they have been extensively studied in the plasticulture field [132,146]. One of the most studied applications has been polymeric mulch films, which undergo several steps in biodegradation. This process involves a period of intense photodegradation when the mulch film starts crosslinking and eroding, which is followed by an intensive period of biodegradation [64,145,147,148]. Figure 9 shows a typical life cycle of polymeric mulch films in agriculture soils.

### 4.2. Home and Industrial Composting Environment

**Figure 10:**
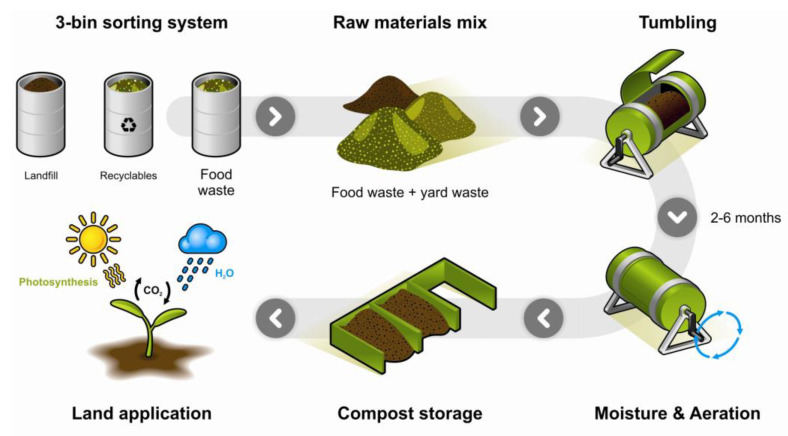
Home composting representation. Kitchen and garden waste are the main raw materials for home composting. Food waste along with yard trimmings are piled up in small-scale composters to form a compost heap. Organic materials are added periodically, with frequent turning to produce finished compost, which can be later used as a soil conditioner. Type of home composting operations vary widely [54] (Copyright 2008. Reproduced with permission from Wiley & Sons, Ltd.).

Home composting is garnering interest since it can be very instrumental in diverting household organic fraction waste from going to landfill [149]. Additionally, as consumers are becoming more aware of plastic pollution, home composting has become important as a potential methodology to reduce organic waste and contaminated packages that cannot be efficiently recovered or diverted through the MSW management system. Home composting is described as the natural aerobic decomposition of organic waste or materials, usually in small-scale composters by “slow-stack” treatment methods where temperatures are in the psychrophilic (0–20 °C) to mesophilic (20–45 °C) range [150]. Home composting can also be labeled as “backyard” or “composting at home”. However, the terminology varies in different geographical regions worldwide since “composting at home” may imply composting in designed vessels inside the apartment or house [151,152], and “backyard” composting may refer to uncontrolled composting units outside the house subject to the environmental conditions. The typical matrix for home composting includes food waste, and garden waste such as weeds and leaves (Figure 10).

Many factors, such as temperature, pH, moisture, substrate, C/N ratio, and microbial populations, affect the composting process [153,154]. Home composting is a less controlled process in comparison to industrial composting. Usually, it never reaches the high temperatures of the thermophilic range for long periods of time, as seen in industrial composting. The small installation size, accompanied by difficulties in reaching an optimum control of factors, results in home composting requiring a longer time to achieve a mature compost [142]. The material volumes that can be handled and the abundance of microorganisms are lower for home composting settings. In addition, seasonal changes can influence “backyard composting” depending on the geographical location, and hence, lower and more variable temperatures are inevitable.

One advantage is that it can be helpful in rural and suburban areas where the collection of organics is limited or there is no infrastructure for industrial composting [149,155,156].

**Figure 11:**
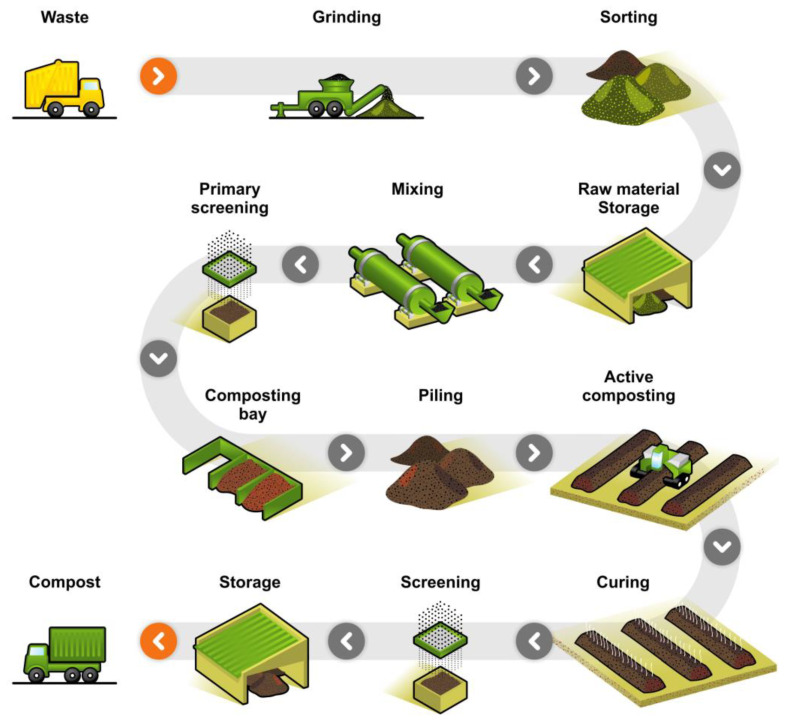
Industrial composting process. Materials collected from curbside bins are sorted to remove any large contaminants present. Feedstock comprises yard, kitchen waste along with compostables mixed in the right proportions. After an initial stage to kill pathogens and seeds’ germination, the feedstock is arranged in long piles and turned frequently to provide aeration. The active composting stage ensures that ideal conditions are maintained to ensure successful breakdown by the microbes present to achieve the desired compost. Then, the compost is cured, screened for any remaining contaminants and is ready for commercial use [54] (Copyright 2008. Reproduced with permission from Wiley & Sons, Ltd.).

Industrial composting is designed to handle large volumes of yard, food, and manure waste [54,154,157]. By employing better aeration, moisture control, and higher temperatures, the biodegradation in industrial composting is accelerated significantly in comparison to natural and home composting processes. The industrial composting process requires a proper system in place for collection of wastes and a good infrastructure (e.g., windrow, aerated static piles, and in-vessel composting) [158]. Biodegradation in industrial composting takes place mostly in the thermophilic temperature range. Figure 11 shows a representation of an industrial composting process.

The composting process follows four main stages. The first stage is the mesophilic stage (20–45 °C), where microorganisms adapt and decompose the simplest organic, degradable substances into CO_2_ and water in an exothermic reaction. The high amount of substrate ensures high microbial activity, which leads to the generation of large quantities of metabolic heat energy that causes the temperature to rise swiftly. The second stage is the thermophilic stage (45–60 °C) where bacteria and fungi mesophiles become less active and are replaced by thermophiles. As the temperature rises above 55 °C, microorganisms such as pathogens are destroyed. For safety reasons, several certifying entities require that the temperature reach above a certain temperature, such as 55 °C, and remain at that level for some time, such as 15 days, to ensure that the resulting compost is pathogen free [159]. Temperatures in some industrial compost facilities during the early stages commonly reach values of c. 70 °C [160]. Such high temperatures accelerate the disintegration process of high-energy carbohydrates and structurally complex molecules. As the disintegration process comes to an end, there is no longer any supply of these high-energy compounds, and the third stage kicks into action where the mesophiles take over once again. The third stage is a transition stage from high to low temperature. The final stage, also called “curing” or maturation, can take several months to result in stabilized compost [142]. The total composting time varies in systems used worldwide, from two to more than six months; thus, certified compostable packages can encounter difficulties to fully disintegrate in some operations [161].

### 4.3. Aquatic Environment

Natural aquatic environments (i.e., oceans, rivers, and lakes), unfortunately, are environments where discarded polymers from activities such as fishing and shoreline recreation are commonly found [8,13,162]; however, these are not formal waste management scenarios and must not be considered as such. The natural aquatic environment is a non-desired end-of-life scenario due to the creation of white pollution and a lack of proper conditions for biodegradation and control of the process due to its complexity [163]. Biodegradation in the aquatic environment can happen in lakes, rivers, and oceans as well as in reservoirs and wastewater facility treatments (aerobic or anaerobic); however, our discussion is focused on the natural aquatic environments.

**Figure 12:**
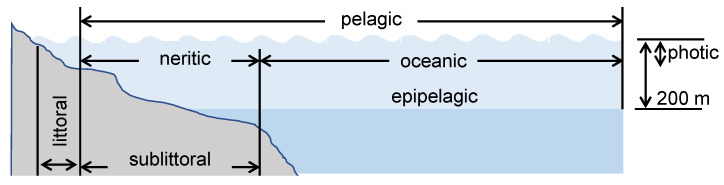
Main habitats to consider when addressing the degradation of polymers in the marine environment. The pelagic zone, an illuminated and aerated column of water; the littoral zone, which is the beach sediment periodically covered by water due to waves or tide; and the sublittoral zone, which is the seabed interface up to 200 m in depth that is aerated and photosynthetically active. Adapted from [170].

Geographical considerations of the aquatic environment play an important role in understanding the presence and flow of plastics. Lakes are generally low-flow environments and act as a point for the accumulation of plastics and microplastics [164]. Rivers are considered the essential route for transporting plastics to the ocean from the mainland. Considering their proximity to urban and industrial areas, rivers become an easy access point to the marine environment with respect to plastic pollution. Plastics are extensively carried out during floods in cities with poor waste management systems [9]. For the marine environment (seawater), three main habitats can be considered when addressing the degradation of plastics (Figure 12): the pelagic zone, an illuminated and aerated column of water; the littoral zone, which is the beach sediment periodically covered by water due to waves or tide; and the sublittoral zone, which is the seabed interface up to 200 m in depth that is aerated and photosynthetically active. The physical and chemical properties of seawater, including the essential nutrients for living organisms, vary with the depth, latitude, and proximity to land. Because of this variation, the microbial populations within seawater also vary. Furthermore, the degradation process of the plastics entering this environment can be altered by agitation and turbulence caused by ocean currents, salinity, temperature gradients, and solar radiation, among others [165,166].

The biodegradation of polymers in aquatic environments is described in terms of scarce evolution for synthetic biodegradable polymers. However, high efficiency has been reported for natural polymers as cellulose, starch, and PHAs regardless of the low temperatures reached. The “plastisphere,” the development of biofilms on the surface of polymers present in water, has been extensively studied to elucidate the main components and behavior of microorganisms during the colonization and depolymerization of polymers in aquatic environments [167,168,169].

## 5. Factors and Properties That Affect the Degradation Rate

The rate of polymer degradation is affected by the degradation mechanisms, the environments, and the polymer properties. This framework creates a complex interplay governing what is reflected in the rate and efficiency of the whole degradation process. In the next subsections, we selectively discuss factors important for mesophilic biodegradation and correlate these factors to the information already provided about mechanisms and environments. Detailed discussions of these factors are also provided in selected reviews [42,44,47].

### 5.1. Environmental Factors

Factors that can affect the degradation rate of a polymer are related to the environment where the degradation process takes place, and they include thermal energy (heat), acidic/alkaline media, moisture, aeration, and microbial populations. Some of these factors are more relevant or critical than others and are important during the abiotic and biotic degradation stages, affecting both the polymer’s properties and the microbial activity.

#### 5.1.1. Heat

The amount of thermal energy, identified as the system’s temperature, is one of the main factors affecting the rate of abiotic and biotic degradation and varies with the environment (Table 3). At an early stage in the degradation process, mechanisms such as chemical hydrolysis can be dominant, and the temperature plays a crucial role in the rate [78,171]. For example, for PLA, the chemical hydrolysis is dependent on the temperature since a large initial reduction in *M_w_* is needed before microorganisms can assimilate the by-products [78]. Higher temperatures activate chain mobility, increasing free volume and polymer rearrangements. If the temperature is higher than the *T_g_* of the polymer, mobility and reaction are accelerated, increasing the rate of polymer degradation (Table 4). Furthermore, the presence and potential growth of different microorganisms depends on the environment temperature, and a change in temperature regulates both presence and activity [117].

#### 5.1.2. Moisture

The presence of water plays a crucial role in the degradation of hydrolyzable chemical bonds, such as in polyesters, since they are susceptible to chain scission reactions [172,173]. Furthermore, microorganisms need water to transport nutrients through the cell membrane and for growth. The amount of water in different environments, such as soil and composting, can create different surroundings for the microorganisms. Low levels of moisture can lead to dry environments with low biological activity [174] while high values of moisture can lead to loss of porosity of the matrix (soil or compost), turning the process into an anaerobic one [175]. Pore spaces are essential for the normal airflow and aerobic regimen; the optimal humidity range for microbial activity is a function of the percentage of pore space needed that does not obstruct the airflow required for microbial activity [176]. For example, for the composting process, an optimal moisture content range is 45 to 65% [154].

#### 5.1.3. Acidic and Alkaline Media

Acidic or alkaline media can modify the rate of reactions and the mechanism of hydrolytic degradation [78]. For example, for PLA in acidic conditions, the hydrolysis proceeds via a chain-end scission, while in alkaline solution, the hydrolysis takes place via backbiting [78]. In the case of PCL films evaluated at extreme pH values (1 and 13) at 37 °C, different behavior was observed for reduction in *M_w_* and crystallinity, suggesting a surface erosion process in alkaline media and bulk erosion in acidic media [177]. During the biodegradation process, pH values close to neutral are highly favorable for the growth of microbial populations. In soil environments, a pH range close to alkaline-neutral values is favorable for bacteria populations, whereas fungi are more tolerant to acidic and alkaline media; fluctuations of pH are considered a harmful situation for living organisms [142].

#### 5.1.4. Light and UV Radiation

If sufficient energy is absorbed by light and UV radiation, polymers can be subjected to photodegradation, experiencing changes in their chemical structure and physical properties. Light and UV radiation are important in agricultural soils and aquatic environments. So, photodegradation can be the precursor of the degradation process before microorganisms can use by-products [71,148].

#### 5.1.5. C/N Ratio

Microorganisms need carbon as a source of energy and nitrogen to synthesize amino acids, proteins, and nucleic acids [154]. The C/N ratio is a key parameter in environments such as compost and soil. Optimal values for the C/N ratio in compost and soil are in the range of 15:1 to 30:1. During the active aerobic phase of breakdown, microorganisms use around 30 parts of carbon for each part of nitrogen due to the high energy requirement. If carbon levels are higher, microorganisms need to undergo several life cycles to oxidize the excess carbon, slowing down the biodegradation process. If carbon levels are low, microorganisms do not have a sufficient energy source to survive [154].

#### 5.1.6. Oxygen Flow and Porosity

Aeration and porosity are key factors for the normal activity of the microbial population in soil and compost environments. To maintain aerobic conditions, the porosity should allow O_2_ concentrations of around 5%. Porosity is highly correlated with the airflow within a matrix. Low porosity hinders air flow, whereas high porosity can lead to excessive aeration and low water retention capacity. The shape, size, and structure of the particles on the matrix (soil or compost) affect its texture. Therefore, a tight packing arrangement reduces the porosity, and the compressed matrix impacts the airflow [154].

### 5.2. Polymer Properties

Factors affecting degradation associated with the bulk polymer matrix can be categorized as chemical structure and physical properties such as morphology, crystallinity, constitutional unit, flexibility, crosslinking, *M_w_*, tacticity, density, shape, and polarity. The surface properties affecting degradation are related mostly to hydrophobic/hydrophilic ratio, roughness, surface energy, and available surface area.

#### 5.2.1. Bulk Properties

*Chain flexibility*. A polymer chain that is highly flexible is more accessible to attack by microorganisms. Longer aliphatic chains can exhibit high biodegradation rates. However, aromatic rings can act as obstacles, providing steric hindrance to the enzyme attacking the ester bonds, thereby lowering the rate of biodegradation [84]. During the depolymerization step, enzyme binding is favored by the high flexibility of the polymer chains. In this sense, it is aptly recognized that microorganisms are more likely to start the biodegradation process in the amorphous region of the polymer [42,43]. Polymers with *T_g_* values in or below the mesophilic range, such as PCL, PBS, PBAT, PHAs, and PGA, will be more flexible in favoring chemical and enzymatic hydrolysis in the mesophilic range (see Table 4). Flexibility and mobility are enhanced by copolymerization, blending, or by increasing the temperature, and they are reduced by crystalline domains [94,178].

*Chemical structure (functional units and functional groups).* Chemical structure is an inherent property of a material and determines whether the polymer is prone to undergo biodegradation. The chemical structure depicts the spatial arrangement of chemical bonds and atoms in the molecule influencing the molecular geometry and governs how the molecules are packed together, allowing the formation of crystalline or amorphous regions [95]. Modifications such as the inclusion of functional groups by copolymerization in the main chain of initially non-biodegradable chemical structures can make a polymer more prone to biodegradation [50]. The addition of functional groups also can impart a hydrophilic nature to a hydrophobic polymer, thus improving its likelihood of undergoing biodegradation [121].

*Chain structure configuration (side chains and crosslinking)*. The length of side chains influences the degradation process. For example, Li et al. [179] concluded that the enzymatic degradation of PHA was dependent on the length of side chain in the PHA structure. Crosslinking can occur and play a significant role in polymer mass transfer properties and chain flexibility hindering biodegradation. Kijchavengkul et al. demonstrated that increasing the amount of crosslinking reduces the biodegradation of PBAT [180].

*Crystallinity.* Crystallinity can increase the stiffness and density of a polymer [42]. A high crystalline fraction decreases the abiotic and biotic degradation rates. The amorphous region is more susceptible to chemical hydrolysis due to the ease of water diffusion. A characteristic of the crystalline region is its low mass transfer to gases and vapors, decreasing the rate of the hydrolytic degradation [181,182]. Extracellular enzymes mainly attack the amorphous region of the polymer structure [115,183]. Biodegradable polymers are, in general, semicrystalline polymers with a crystalline and amorphous region. 

*Molecular weight (M_w_)*. To obtain polymers with usable thermal, mechanical, and barrier properties, a high *M_w_* is required. However, microorganisms assimilate polymers when selected thresholds of low *M_w_* fractions of the polymer are reached. The higher the *M_w_* value of the polymer residue, the harder it is for microorganisms to assimilate the chain segments, which reduces the rate of the biodegradation. So, a critical threshold low *M_w_* value must be reached to kick off the degradation by enzymatic attack [84]. Generally, this *M_w_* is attainable by a precursor degradation mechanism such as photodegradation or chemical hydrolysis, as with polyesters. In the case of PLA, the polymer first undergoes primarily chemical hydrolysis, accelerated under industrial composting conditions, until reaching a *M_w_* ≤10 kDa, and then, enzymatic activity becomes the dominant degradation mechanism, featuring a high mineralization rate [125].

*Density and porosity*. Denser and more compact polymers have low chances of experiencing water diffusion. For polyesters, chemical hydrolysis is generally the initial trigger mechanism of degradation, mostly through a bulk erosion process, so water diffusivity of the polymer plays a crucial role. One way to modify the diffusion or the hydrophilicity of a polymer matrix is by blending different polymers. So, biodegradable blends and copolymers can be used to tailor some of these bulk properties. Blends of PLA and TPS have shown higher biodegradation rates [184].

#### 5.2.2. Surface Properties

The hydrophobic/hydrophilic ratio, surface roughness, surface energy, and surface/volume ratio are the more relevant factors during the degradation process. Chemical hydrolysis is highly affected by the hydrophobic/hydrophilic ratio of the polymer surface. Furthermore, enzyme activity, biofilm formation, and colonization are also linked to surface properties.

*Hydrophobic/hydrophilic ratio*. In the case of isotropic polymers, surface and bulk water sensitivity plays a major role in the degradation process. Hydrophobic surfaces will not allow water to be adsorbed and will delay water uptake, so that any degradation mechanism triggered by water diffusion will be delayed. Table 5 shows that polymers with hydrophobic surface and high-water diffusion, such as the polyester PLA, mostly degrade under a bulk degradation process [56]. So, by tailoring the surface and bulk hydrophobicity and the water diffusion of the polymer matrix, the overall chemical hydrolysis can be controlled, as shown for PLA [172]. In terms of enzymatic activity, a hydrophobic/hydrophilic balance allows the presence of necessary water for optimal microbial activity [168]. Some studies have demonstrated that biofilms develop faster on hydrophobic nonpolar surfaces [185]. However, Tsuji et al. reported an alkaline treatment to increase the hydrophilicity of PLLA and PCL to improve enzymatic attack. The effect was important for PLLA films, where enzymatic attack by Proteinase K was higher on hydrophilic surfaces [186,187]; however, the attack by lipases on PCL films remained unchanged [187]. The fact that lipases need a hydrophobic surface to be active could be an important conditioning of the scarce activity on PCL films. Furthermore, the exposure to hydrophobic surfaces has been reported to be a relevant signal for the production of extracellular enzyme cutinases by fungi to act on the surface of polyesters such as PCL, PBS, and PBSA, among others [188]. Tribedi et al. reported the effect of cell hydrophobicity when comparing the enzymatic esterase activity of two strains of *Pseudomonas* on the surface of PES. The strain with higher hydrophobicity also showed higher microbial activity, which is indicative that the interaction and hydrophobic balance between the microorganism and polymer surface is also relevant for microbial and enzymatic activity [189].

*Surface roughness*. Surface roughness is a measure of the finely spaced micro-irregularities on the surface texture and depicts the irregularities on the polymer surface. Some researchers have used surface roughness as an indicator of surface biodegradation [145,190]. The types of microbes able to colonize a surface and the formation of biofilms depend on the surface roughness. Increased roughness favors bacterial adhesion because of the greater area of contact between the polymeric material and the bacterial cells [191]. A rough surface offers micro- and nano-irregularities in the range of 0.5 to 2 μm, which appear as voids and can provide sites for microorganisms to attach and eventually access the polymer chains, increasing the rate of biodegradation [192,193].

*Surface area*. The shape (e.g., film, pellet, powder, and fiber) and size (macro, micro, and nano) of the polymer play important roles during the degradation process [47]. For example, thick polyester samples take more time to biodegrade [89]. The surface area available has a high effect on the rate of biodegradation; as the surface to volume ratio increases with time, so does the speed at which biodegradation occurs. Pits and cracks continue to increase as time proceeds, and gradually, the sample shape and size change, enabling access to the inside of the matrix [194]. Extracellular enzymes are highly active on the surface of a polymer, since they are relatively large to penetrate the bulk. Hence, increasing the surface area available for enzymatic attack translates into an increase in the kinetics of the biodegradation process. Herzog et al. [123] showed that the enzymatic degradation of a polyester by *Candida cylindracea* at 40 °C was more effective on nanoparticles (100 nm diameter) than on thick films (110 μm thickness) of the same polyester. The effect of morphology on the water biodegradation of PHBV was evaluated by Komiyama et al. [195]. Samples evaluated in powder form showed the faster biodegradation due to the larger surface area available for biofilm formation and microorganism attack in comparison to film, undrawn fiber, and fivefold drawn fiber.

## 6. Biodegradation Assessment

The misuse of the terms “biodegradable” or “biodegradation” has given rise to inflated and unsubstantiated claims. Claims about general biodegradable products that are used to deceive consumers into believing that products are environmentally friendly have been coined “greenwashing.” It is essential to avoid such false claims, guarantee transparency to consumers, and stop the unqualified use of vague terms. Certification for biodegradation, per se, does not exist worldwide. Some polymer and paper materials are certified for biodegrading in specific environments such as home and industrial composting, soil, and water [196]. Standards and methods have been developed to aid certification, avoid confusion, and define the environment and conditions in which the samples can be biodegraded [197].

### 6.1. Standards for Evaluation of Biodegradation at Mesophilic Conditions

Several organizations are associated with developing the standards for the biodegradability of materials in different environments for different countries and world regions [197]. Various reviews and reports have provided the standards available for biodegradation in soils [198], aquatic environments [199], or home and industrial composting [200,201]. In this review, we specifically summarize, in Table 6, the different standards used to assess biodegradability under aerobic conditions for mesophilic temperatures and tracking evolution of CO_2_ and O_2_ demand and cite published works that reported the use of these standards. Furthermore, standards with specifications of materials to be evaluated and for certification are described in ASTM 6400, ISO 17088, and EN13432. The environmental conditions in which the biodegradation takes place are an important aspect since the biodegradability of a material differs from one environment to another. The development of standards for assessing biodegradation in different environments is essential [202,203].

### 6.2. Methods for Biodegradation Assessment

Different methodologies, both quantitative and qualitative, are used to determine the biodegradation process. When used in combination, the different methodologies help to recognize if there is any disagreement among the achieved results. In addition, supporting quantitative methodologies, such as CO_2_ evolution and *M_w_* reduction, with qualitative methodologies, such as scanning electron microscopy (SEM), visual observation, and spectroscopy, is helpful in corroborating the biodegradation of the material under study. The main methodologies to assess and report the degree of biodegradation in aerobic conditions have been summarized in several reviews [43,201,242,243,244]. The oldest and most common methodology is the gravimetric reduction in weight or mass loss of the material under biodegradation. Significant deterioration in mechanical properties has also been reported as a degree of biodegradation. However, macro visualization, mass loss, and deterioration of mechanical properties are methods for the approximate assessment of biodegradation. These methods are more related to physical degradation of the material and not to the biological process conducted by a population of microorganisms. In general, they are more useful for gaining insights during the early step of polymer biodegradation such as during abiotic degradation or during biofilm formation on the surface of the polymer.

For enzymatic activity, clear zone formation, turbidimetric assays, and techniques that monitor the release of soluble products into the supernatant solution such as total organic carbon (TOC) and spectroscopy combined with chromatography have been reported. Nowadays, the use of microbalance with dissipation monitoring measurement constitutes an additional analytical technique to evaluate the evolution of the enzymatic hydrolysis of hydrolysable polymers.

For tracking CO_2_ evolution and mineralization, respirometric methods has been developed and are supported by standards for assessing the conversion of the carbon present in the polymer to CO_2_. Furthermore, standards also describe for specific environments the measurement of biochemical oxygen demand (BOD) instead of CO_2_. The radio labeling and tracking of carbon has been reported as an adequate technique to complement respirometric methods.

Associated with each of the main evaluation methodologies are several techniques used to quantify the degree of biodegradation. Table 7 lists published studies conducted to measure biodegradation using techniques to measure CO_2_ and/or O_2_ under aerobic conditions at mesophilic temperatures. This section provides a brief description of the methodologies used and the published studies using those methodologies in the mesophilic range.

#### 6.2.1. Mass Loss and Mechanical Properties Deterioration

Measurement of mass loss is the most commonly used method to indicate the extent of degradation and is indicated as mass loss measured from the samples retrieved during the degradation test [201]. Mass loss is used mostly to designate the degradation occurring on the polymer surface and is contingent on the disintegration phenomena. Many researchers have reported the use of mass loss determination to indicate that the material has undergone degradation. Furthermore, the deterioration of mechanical properties (assessed on films, sheets, or dumbbell specimens) indicative of degradation by the action of abiotic mechanisms has been reported along with mass loss.

*Quartz crystal microbalance with dissipation* (QCM-D). In terms of enzymatic degradation, a microbalance weight loss technique in the nanogram scale has been reported during the enzymatic hydrolysis of aliphatic and aromatic polyesters such as PCL and PBAT. This is a unique approach to monitor the dynamic of the enzymatic hydrolysis and has showed high sensitivity [270,271,272,273,274].

#### 6.2.2. Macro and Micro Visual Analysis of the Polymer Surface

Macro visual analysis is the second most used technique after mass loss. Macro visual changes of the polymer do not necessarily indicate biodegradation, but these changes are usually the first evidence of microbial colonization and biofilm formation.

Micro visual inspection using microscopic techniques such as SEM, transmission electron microscopy (TEM), or atomic force microscopy (AFM) can impart more knowledge regarding the biodegradation process at the early stage, specifically biofilm formation and the structure of the sample [43]. The topographical changes occurring in the polymer are usually seen as the formation of holes, cracks, cavities (material erosion), discoloration, or surface roughness [275]. Kijchavengkul et al. studied the surface evolution during biodegradation of PBAT films and demonstrated the consequences of the degradation by using SEM methodology, among other techniques [276]. For PBAT samples with c. 30% or less crosslinking, biofilm formation was observed. A large number of microbes consumed PBAT samples, creating pits in the film surface. For samples with more than 30% crosslinking, no cavities were observed on the PBAT film surface, indicating that increased crosslinking results in reduced biodegradation [276]. Shah et al. reported changes in surface morphology, such as pit formation and erosion, due to the biodegradation of PHBV films in a basal salt medium after two weeks of immersion [277]. Techniques such as TEM and AFM were extensively used to identify the chemical and enzymatic degradation of polyesters [278,279,280,281].

#### 6.2.3. Chromatography

*Size exclusion chromatography* (gel permeation chromatography) is used to study the reduction in *M_w_*. Reduction and distribution in *M_w_* are preferred parameters that provide evidence of the degradation process. When accompanied by mineralization data, the *M_w_* reduction can provide more insights into understanding the process.

*High-performance liquid chromatography* is widely used for the qualitative and quantitative analysis of soluble compounds derived from the enzymatic activity released into solution.

Lu et al. [282] examined the biodegradability of PPC/starch composites in soil at room temperature; the study of *M_w_* change for unburied, 40 and 180 days along with weight loss and other qualitative techniques such as FTIR, SEM, and photographs helped the researchers conclude that PPC was the last component to biodegrade post microbial colonization and starch degradation. Reduction in *M_w_* by chemical hydrolysis has been reported for aliphatic and aromatic polyesters such as PLA, PCL, PHB, and PBAT, among others [78,283,284].

#### 6.2.4. Spectroscopy

A qualitative way to assert biodegradation is by identifying the chemical changes in the polymer structure [201]. These changes could translate into the formation of low *M_w_* compounds resulting from polymer degradation. Changes in the molecular structure can be identified by various spectroscopic analysis methods.

*Nuclear magnetic resonance (NMR)*: The nuclei of any given type (C, H, N, P, or O) resonate at different energies. The information from the NMR signal (position and pattern) gives critical information about the nuclei environment and presence [93,285]. NMR has been reported for the degradation of different polymers in different environments. Kijchavengkul et al. studied the biodegradation of PBAT in compost and tracked the evolution of the BT and BA dimers using ^1^H NMR and showed that the soft aliphatic portion and the amorphous region are more susceptible to hydrolysis and biodegradation than the rigid aromatic portion and the crystalline region [93].

*Fourier-transform infrared spectroscopy (FTIR)*: FTIR analysis of any given material provides a specific fingerprint spectrum for that material, and the appearance and disappearance of peaks associated with the functional groups can help explain the changes happening in the material structure [93,201].

*Mass spectroscopy* is an analytical technique widely used to identify products during the enzymatic degradation of polymers. In general, it is used along with techniques such as liquid chromatography.

Weng et al. studied the biodegradation of PHB/PLA blends buried in soil at different depths at c. 20 °C; the FTIR spectra showed that the peaks in the 4000 to 3000 cm^−1^ region were broad in nature due to the formation of -OH and -COOH groups after degradation [286]. Furthermore, Mbarki et al. [287] conducted both the FTIR and NMR analysis on PDLA samples immersed in the soil/liquid culture at 37 °C and found no significant difference in the chemical structure before and after immersion (45 days for FTIR and 28 days NMR); the conclusion derived was that the biodegradation phenomena was only surface and not bulk.

#### 6.2.5. Plate (Clear Zone Formation) and Turbidimetry Assays

Plate tests were originally designed to gauge the resistance of plastics to degradation via microorganisms. However, in addition to testing resistance, they are now used to see if the polymer can support the growth of microorganisms through biofilm formation. The polymeric material is dispersed in a petri dish containing a mineral salts agar medium that serves as the sole carbon source. The polymer in the surface, suspended in the medium, is then inoculated with microorganisms and held for a predetermined amount of time at a constant temperature to allow the microorganisms to grow. The formation of a halo or clear zone around the microorganism colony marks an end for this test since the clear zone indicates that the microorganism can at least depolymerize the polymeric material. The test is also used in screening, isolating, and identifying the potential degrading microorganisms for any given polymer [288,289]. Urbanek et al. isolated, screened, and assessed the degrading capability of Antarctic soil microorganisms on PCL, PBS, and PBSA at low temperature with the help of the clear zone formation technique [290]. Table 8 presents several works that have used clear zone formation for studying the enzymatic degradation of biodegradable polymers.

#### 6.2.6. Respirometric Tests for CO_2_ Evolution and Biochemical O_2_ Demand

These tests involve measuring the consumption of O_2_ or formation of CO_2_ under aerobic conditions. The CO_2_ evolved can be measured by three different techniques [54]: in cumulative measurement respirometry (CMR), the evolved CO_2_ (trapped in basic solution such sodium hydroxide or barium hydroxide) is quantified by the titration method [291]; in gravimetric measurement respirometry (GMR), the evolved CO_2_ is trapped in absorption columns, and the weight increase is used to quantify the amount of CO_2_ [291]; and in direct measurement respirometry (DMR), the evolved CO_2_ is quantified by means of an inline non-dispersive infrared gas analyzer or gas chromatograph [125]. Kale et al. compared the use of CMR, GMR, and DMR to assess the biodegradation of PLA under simulated composting conditions, and they found a similar evolution of biodegradation [291]. They concluded that the biodegradation process is further dependent on various factors, including shape, size, thickness, and sample/compost ratio, among others. The advantages and disadvantages associated with these techniques are explained in detail elsewhere [276].

Techniques measuring O_2_ consumption, reported as BOD, are assessed in specific aquatic environments as sewage sludge and wastewater. However, standards have been also developed for assessing O_2_ consumption in soil environments (Table 6).

#### 6.2.7. Radiolabeling

An understudied approach for assessing the degree of biodegradation is the use of radiolabeled carbon. This is one of the absolute tests to determine biodegradation and involves tracking carbon from biodegradable polymers into CO_2_ and biomass. The approach is based on labeling the carbon atoms in the polymer backbone with carbon isotopes: ^13^C (stable in nature) and ^14^C (radioactive) [292,293].

Early works conducted by Albertsson et al. showed that the technique of radiolabeling polymers using ^14^C was useful not only for detecting the biotic stage of the biodegradation process but also the abiotic stage [294,295,296,297]. In this sense, PE films produced using a ^14^C marker showed ^14^CO_2_ evolution of the carbonyl oxidized by-products when the films were exposed to soil [294].

Zumstein et al. employed the use of ^13^C labeled polymer along with isotope-specific analytical methods (i.e., cavity ring-down spectroscopy) to track the biodegradation of PBAT in soil [148]. This technique allowed for tracking of the basic biodegradation steps by distinguishing the labeled PBAT CO_2_ from the CO_2_ evolved due to the mineralization of organic matter in the soil.

In summary, two or more methods are commonly employed together to determine biodegradation. The change in *M_w_*, weight loss, and surface analysis are used widely, but these alone do not guarantee biodegradation, and at most, they hint toward disintegration of the material under study. The evolution of CO_2_ and radiolabeling represents the complete assessment of the breakdown of the material into biomass, and they need to be employed on a more regular basis for biodegradation studies. Although the respirometry method gives the mineralization value, radiolabeling is far more advanced by showing the actual integration of polymer carbon into the microbial biomass.

With respect to standards, there is no international standard specifying how home composting should be conducted for the effective biodegradation of biodegradable polymers. In addition, many standards for determining polymer biodegradation in aquatic environments, as listed in Table 6, mention temperatures (laboratory-simulated settings conditions) that are much higher than the actual conditions encountered in real-world environments. In general, adaptations of the international standards to specific conditions are implemented to assess and report, for example, the biodegradation of polymers in home composting or at mesophilic temperatures.

## 7. Microorganisms and Enzymes Able to Biodegrade Polymers

The ability to degrade biodegradable polymers is widely distributed among bacteria, fungi, and actinomycetes, and there is much variation in ability. Table 8 lists extracellular enzymes and/or microorganisms able to biodegrade polymers in different mesophilic environments, as reported in the published literature.

### 7.1. Microbial Population

Some microorganisms can digest several polymer structures in different environments, and degradation rate efficiency can differ. A high portion of the published works have reported the digestion activity of a specific microorganism in the highly controlled conditions of incubated or culture media; under these conditions, the polymer substrate is mostly the only source of nutrient for the microorganism. In contrast, in less restricted environments, such as soil, home composting, industrial composting, or aquatic environments, the complexity of the biological activity process increases, and several sources of substrates and microorganisms may be available. In natural soil or aquatic environments, an active population of microorganisms with different requirements, in terms of nutrients and optimal growth conditions, are competing or working cooperatively for the resources available.

The presence of microorganisms and the formation of a biofilm, due to the colonization of the polymer surface, creates an effect that sometimes can alter the abiotic degradation of the polymer. For example, when PCL biodegradation was evaluated under low stirring, the impediment of biofilm formation resulted in a higher weight loss [298].

**Table 8:** Enzymes and/or microorganisms with activity for degrading biodegradable polymers when tested in mesophilic conditions. Different parameters such as the enzymes released, microbial species used, the environment from which the microorganisms were isolated/testing media, polymer studied, the temperature and pH for conducting the biodegradation study, optimal conditions for the microorganisms, and studies reporting them are mentioned.

Enzymes *	Microorganism *	Environment	Polymer	T (°C), pH	Optimal Conditions of T (°C) and pH	Reference
Alcalase (3.4.21.62)	*Bacillus licheniformis* (B)	Buffer solution	PLA	40, 8.0	60, 9.5	[299]
Amidase (3.5.14)/esterase (55 kDa)	*Rhodococcus equi* strain TB-60	Soil/culture	PU	30, 7	45, 5.5	[300]
Carboxyl esterase (3.1.1.1)	*Alcanivorax borkumensis* (B), *Rhodopseudomonas palustris* (B)	Culture	PCL, PDLLA, PBSA	30, 8.0	30–37, 9.5–10	[301]
Carboxyl esterase	*Alcanivorax borkumensis* (B)	Culture	PES, PHBV, PDLLA	30, 8.0	55–60, 9.5–10	[301]
Chymotrypsin (3.4.21.1)	-	Culture	PLLA, PEA	37, 7.0	-, -	[302]
Cutinase (3.1.1.74) (21.6 kDa)	*Aspergillus oryzae RIB40 (F)*	Culture	PBS, PBSA, PLA	37, 8.0	35–55, 9.0	[188]
Cutinase	*Alternaria brassicicola (F), Aspergillus fumigatus (F), Aspergillus oryzae (F), Humicola insolens (F), Fusarium solani (F)*	Culture	PCL	40, 3, 5, 8	-, -	[303]
Cutinase	*Fusarium solani* (F)	Buffer solution	PBAT	30, -	-, -	[148]
Cutinase (21 kDa)	*Cryptococcus magnus* (F)	Larval midgut of stag beetle (Aegus laevicollis)/culture	PBS, PBSA, PCL, PDLLA, PLLA	30, 7.4	40, 7.5	[304]
Cutinase	*Fusarium solani* (F)	Buffer solution	PCL	37, 7.2	-, -	[305]
Cutinase (20 kDa)	*Fusarium sp. FS1301 (F)*	Soil/liquid culture	PBS, PCL	30, -	50, 8.0	[306]
Cutinase (19.7 kDa)	*Paraphoma*-related fungal strain B47-9 (F)	Barely phyllophane/liquid culture	PBAT, PBS, PBSA, PCL, PDLLA	30, 7.2	45, 7.2	[307]
Cutinase	*Pichia pastoris* (F)	Buffer solution	PBS	37, 7.4	-, -	[308]
Cutinase	-	Culture	PBS, PBA	37, 7.4	-, -	[309]
Cutinase (20.3 kDa)	*Pseudozyma antarctica* JCM 10,317 (Y)	Culture	PBS, PBSA, PCL, PLLA, PDLLA	30	40, 9.5	[310,311]
Cutinase	*Fusarium solani* (F), *Fusarium moniliforme* (F)	Culture	PCL	22	9–10	[312]
Cutinase	*Bacillus* sp. KY0701	Culture	PCL	30, 7	50, 7	[313]
Cutinase	*Aspergillus oryzae* (F)	Buffer solution	PCL	40, 8	-, -	[314]
Cutinase	*Pseudozyma jejuensis* OL71 (F)	Leaves of Citrus unshiu/culture	PCL	30, -	-, -	[315]
Cutinase-like enzyme (22 kDa)	*Cryptococcus flavus* GB-1 (Y)	Culture	PBSA	30, 6.8	45, 7.8	[316]
Cutinase-like enzyme	*Cryptococcus* sp. Strain S-2 (F)	Liquid culture	PBS, PLA, PCL	30, -	37, 7.0	[317]
Close related to Cutinase	*Pseudomonas pachastrellae* JCM12285^T^ (B)	Marine, coastal seawater/culture	PCL	30, -	-, -	[318]
Elastase	-	Culture	PLA	37, 7.0	-, -	[302]
Esterase (3.1.1.1)	*Aspergillus* sp. Strain S45 (F)	Solid waste dump site/liquid culture	PU	30, 7.0	-, -	[249]
Esterase	*Bacillus* sp. AF8 (B)*, Pseudomonas* sp. AF9 (B), *Micrococcus* sp. 10 (B), *Arthrobacter* sp. AF11 (B), *Corynebacterium* sp. AF12 (B)	Soil/culture	PU	30–35	-, -	[258]
Esterase	Hog liver	Buffer solution	PGA	37, 7.5	-, -	[319]
Esterase	*Bacillus subtilis* (B)	Buffer solution	PCL, PLA	37, -	-, -	[266]
Esterase	*Aspergillus tubingensis* (F)	Soil/solid and liquid culture	PU	(30, 37, 40), (5–9)	37, 7.0	[320]
Esterase	*Bacillus licheniformis* (B)	Compost/liquid culture	PLLA	32, 7.4	-, -	[321]
Esterase	*Alicycliphilus* sp. (B)	Culture	PU	37, 7	-, -	[322]
Esterase	*Leptothrix* sp. TB-71 (B)	Soil, fresh water/culture	PBSA, PES, PCL	30, -	-, -	[323]
Esterase (62 kDa)	*Comamonas acidovorans* strain TB-35 (B)	Soil/liquid culture	PU	30, 7.2	45, 6.5	[324,325,326]
Esterase (28 kDa)	*Curvularia senegalensis* (F)	Soil/liquid culture	PU	(21–25), 30, 35, 45, (4.0–8.0)	-, 7–8	[327]
Esterase (42 kDa)	*Comamonas acidovorans* (B)	Culture	PU	30, 5–8	-, -	[328]
Esterase	*Penicillium verrucosum* (F), *Aspergillus ustus* (F)	Compost soil/culture	PLA	30, 5.6	-, -	[329]
Esterase	*Pseudomonas aeruginosa* MZA-85 (B), *Bacillus subtilis* MZA-75 (B)	Soil/liquid culture	PU	37, 7.0	-, -	[254,255,256]
Esterase	*Pseudomonas aeruginosa* strain S3 (B)	Culture	PLA	30–37, 8	37, 8	[330]
Esterase	*Pseudomonas* (B)	Soil/Culture	PES	30, -	-, -	[189]
Esterase	Porcine liver	Buffer solution	PLA	40, 8.0	40, 8.0	[299]
Close related to esterase	*Bacillus pumilus* strain KT1012 (B)	Soil, water/culture	PES, PCL	30, 7.0	40–45, -	[331]
Lipase (3.1.1.3)	*Rhizopus delemar* (F)	Buffer solution	PLA	37, 7.2	-, -	[332]
Lipase	*Acidovorax delafieldii Strain BS-3 (B)*	Soil/solid and emulsified substrate	PBS, PBSA	30, 7.0	-, -	[333]
Lipase	*Rhizopus oryzae* (F), *Burkholderia* sp. (B)	Liquid culture	PCL	30, -	-, -	[317]
Lipase	*Candida rugosa (F)*	Buffer solution	PCL, PLA	37, -	-, -	[266]
Lipase (36 kDa)	*Aspergillus niger* MTCC 2594 (F)	Liquid culture	PCL, PLA	30, 7	37, 7.0	[334]
Lipase	*Aspergillus oryzae* (F)	Buffer solution	PCL	37, 7.0	-, -	[335]
Lipase	*Aspergillus tubingensis* (F)	Soil/solid and liquid culture	PU	(30, 37, 40), (5–9)	37, 5.0	[320]
Lipase	*Burkholderia cepacia* PBSA-1 (B), *Pseudomonas aeruginosa* PBSA-2 (B)	Soil/culture	PBSA	27, 37	-,	[259]
Lipase	*Candida cylindracea* (F)	Buffer solution	PLA	40, 8.0	40, 8.0	[299]
Lipase	*Candida antarctica* (F)	Buffer solution	PCL, PBS	45, 7.2	-, -	[305,336,337]
Lipase	*Candida rugosa* (F)	Liquid culture	PU	(20–50), (4–9)	35, 7.0	[338]
Lipase	*Chromobacterium viscosum* (B), *Rhizopus orizae* (F), *Rhizopus niveus* (F)	Culture	PCL, PBS, PBSA	37, 7.0	-, -	[339]
Lipase (23 kDa)	*Cryptococcus* sp. MTCC 5455 (F)	Liquid culture	PBAT	25, -	-, -	[340]
Lipase	*Cryptococcus* sp. MTCC 5455 (F)	Buffer solution	PU	30, 7.0	37, (7.0–8.0)	[341]
Lipase	*Lactobacillus plantarum* (B)	Culture	PCL	37, 8.0	-, -	[342]
Lipase (25 kDa)	*Penicillium* sp. Strain 14-3 (F)	Soil/liquid culture	PEA	30, 6.0	45, 4.5	[343]
Lipase	*Pseudomonas* (B)	Buffer solution	PLLA, PCL, PDLLA	37, 7.0	-, -	[344,345]
Lipase	*Pseudomonas cepacia* (B)	Buffer solution	PCL	37, 7,0	-, -	[207]
Lipase	*Pseudomonas cepacia* (B), *Rhizopus delemar* (F)	Buffer solution	PCL, PPS	30, 7.2	-, -	[346]
Lipase	*Pseudomonas fluorescens* (B)	Buffer solution	PCL	37, 7.4	-, -	[347]
Lipase (22 kDa)	*Cryptococcus* sp. (Y)	Buffer solution	PBS, PBSA	30, 7	-, -	[348]
Lipase	*Fusarium solani* (F)	Culture	PCL	22, 6.8	-, -	[349]
Lipase (34 kDa)	*Pseudomonas* sp. Strain DS04-T (B)	Activated Sludge/liquid medium	PLLA, PCL, PHB	37, 8	50, 8.5	[350]
Lipase	*Rhizopus oryzae* (F)	Solution	PBS, PLLA, PBA	40, 5	40, 7	[271]
Lipase	*Rhizopus arrhizus* (F)	Buffer solution	PCL	30, 7	-, -	[187]
Lipase	*Pseudomonas* (B)	Buffer solution	PCL	25, 37, 7	-, -	[351]
Lipase	*Rhizopus oryzae* (F)	Buffer solution	PBAT	30, -	-, -	[148]
Lipase	*Rhizopus delemar* (F)	Buffer solution	PU	37, -	-, -	[352]
Lipase	*Pseudomonas* (B)	Buffer solution	PCL	37, 7	-, -	[353]
Lipase	*Achromobacter* sp (B), *Candida cylindracea* (F), *Rhizopus arrhizus* (F), *Rhizopus delemar* (F), *Geotrichum candidum* (F)	Buffer solution	PEA, PCL	37, 7.0	-, -	[354]
Lipase	*Bacillus* sp. (B)	Soil/culture buffer solution	PBAT	30–37, 7.4	-, -	[355]
Lipase	*Pseudomonas* sp. (B)	Buffer solution	PEA	37, 7.0	-, -	[356]
Lipase	*Stenotrophomonas* sp. YCJ1	Soil/culture	PBAT	30, 7.2	37, 7.5	[357]
Lipase	*Candida Antarctica* (F)	Buffer solution	PBAT	45, 7.2	-, -	[358]
PBAT hydrolase (closely related to lipase)	*Rhodococcus fascians* NKCM 2511 (B)	Soil/liquid culture	PBAT, PCL, PBSA, PES, PBS (low activity)	25, -	-, -	[267]
PBAT hydrolase (closely related to cutinase) (18.9 kDa)	*Rhodococcus fascians* (B)	Liquid culture	PBAT, PCL, PBSA, PES, PBS	30, 7	-, -	[359]
PBAT hydrolase (closely related to Lipase)	*Bacillus pumilus* (B) (NKCM3101, NCKM3201, NCKM3202, KT1012), *Brevibacillus choshinensis* PBATH (B)	Soil/liquid culture	PBAT (low activity), PBSA, PBS, PES, PCL	30, 7.0	-, -	[95]
PLA depolymerase (related to lipase)	*Paenibacillus amylolyticus* Strain TB-13 (B)	Soil/culture	PBS, PBSA, PDLLA, PCL, PES	37, 8	45–55, 10.0	[360]
PBAT hydrolase	*Isaria fumosorosea* strain NKCM1712 (F)	Soil/culture	PBAT, PBA, PBS, PBSA, PES, PHB, PCL	25–45, 7.0	-, -	[268]
PBS-degrading enzyme (44.7 kDa)	*Aspergillus* sp. XH0501-a (F)	Soil/culture	PBSA	30	40, 8.6	[361]
PCL depolymerase (63.5 kDa) (esterase)	*Brevundimonas* sp. strain MRL-AN1 (B)	Liquid culture	PCL, PLA, PES, PHB, and PHBV	37, 7	30, 6–8	[362]
PCL depolymerase	*Penicillium oxalicum* strain DSYD05-1 (F)	Soil/liquid culture	PCL, PHB, PBS	30, 6.8	-, -	[363]
PCL depolymerase	*Alcaligenes faecalis* TS22 (B)	Culture	PCL	30, -	-, -	[364]
PCL depolymerase	*Paecilomyces lilacinus* strain D218 (F)	Soil/solid culture	PCL	30, 5.2	30, 3.5–4.5	[365]
PLA depolymerase (58 kDa)	*Pseudomonas tamsuii* TKU015 (B)	Soil/culture	PLLA	30, 7.0	60, 10	[366]
PLLA degrading enzyme	*Actinomadura keratinilytica* T16-1 (B)	Culture	PLLA	45, 7	45, 6–8	[367]
PHA depolymerase (3.1.1.76)	*Alcaligenes faecalis* (B)	Buffer solution	PHB, PHBV, PHA	37, 7.4	-, -	[368]
PHA depolymerase (48 kDa)	*Pseudomonas stutzeri* YM1414 (B)	Fresh water/buffer solution	PHB	37, 7.4	55, 9.5	[369]
PHA depolymerase	*Ralstonia pickettii* T1 (B)	Buffer solution	PHB, PHBV	37, 7.5	-, -	[179]
PHA depolymerase	*Ralstonia pikettii* T1 (B), *Acidovorax* sp. TP4 (B)	Buffer solution	PHA	37, 38, 7.5, 8.0	-, -	[370]
PHA depolymerase	*Comamonas* sp. DSM 6781 (B), *Pseudomonas lemoignei* LMG 2207 (B), *Pseudomonas fluorescens* GK13 DSM 7139 (B)	Liquid culture	PHB, PHV, PHBV	30, 7.2	-, -	[371]
PHA depolymerase (50 kDa)	*Comamonas testosteroni* (B)	Buffer solution	PHB, PHBV	37, 7.4	-, 9.5–10	[372]
PHA depolymerases (33.8 and 59.4 kDa)	*Pseudomona mendocina* DS04-T (B)	Mineral medium	PHB, PHBV	37, -	50, 8 and 8.5	[373]
PHA depolymerase (intracellular)	*Pseudomonas putida* LS46 (B)	Culture	PHB, PCL, PES	30, 7	-, -	[374]
PHB depolymerase (3.1.1.75)	*Alcaligenes faecalis* (B)	Culture	PHB	37, 7.4	-, -	[375]
PHB depolymerase	*Alcaligenes faecalis (B), Pseudomonas stutzeri (B), Comamonas acidovorans (B)*	Buffer solution	PHB, PEA, PES	37, 7.4	-, -	[376]
PHB depolymerase (57 kDa)	*Aspergillus fumigatus* (F)	Buffer solution	PHB, PHBV, PEA, PES	45, 8.0	70, 8	[377,378]
PHB depolymerase (49 kDa)	*Comamonas testosteroni* strain ATSU (B)	Soil/culture	PHB, PHBV	37, 7.4	70, 8.5	[379]
PHB depolymerase (42.7)	*Aureobacterium saperdae* (B)	Buffer solution	PHB	37, 7	45, 8	[380]
PHB depolymerase (57 kDa)	*Aspergillus fumigatus* 76T-3		PHB, PES, PBS	45, -	55, 6.4	[381]
PHB depolymerase (50–48 kDa)	*Emericellopsis minima* W2 (F)	Wastewater/liquid culture	PHB, PHBV	30, 8.0	55, 9.0	[382]
PHB depolymerase (40 kDa)	*Microbacterium paraoxydans* RZS6 (B)	Dumping yard/culture	PHB	30, -	30, 7	[383]
PHB depolymerase (46.8 kDa)	*Penicillium* sp. DS9701-D2 (F)	Activated sludge/culture	PHB	28–30, 6.8	30, 5	[384]
PHB depolymerase	*Streptoverticillium kashmirense* AF1 (A)	Sewage sludge/culture	PHBV	30, 8	-, -	[385]
PHB depolymerase (50 kDa)	*Acidovorax* sp. strain TP4 (B)	Pond water, river water, farm soil/culture	PHB	30, 8.5	-, -	[386]
PHB depolymerase (47 kDa)	*Arthrobacter* sp. strain W6 (B)	Soil/culture broth	PHB, PHBV	30, 7	50, 8.5	[387]
PHB depolymerase (85 kDa)	*Fusarium solani* Thom (F)	Wastewater/culture	PHB	25, 8	55, 7	[388]
PHB depolymerase (62.3 kDa)	*Bacillus megaterium* N-18-25-9 (B)	Culture	PHB	30–37, 9	65, 9	[389]
PHB depolymerase (44.8 kDa)	*Penicillium* sp. (F)	Culture	PHB	40, 4–6	50, 5	[390]
PHB depolymerase (61.8–70 kDa)	*Marinobacter* sp. NK-1 (B)	Culture	PHB	37, 7.4	-, 8	[391,392]
PHB depolymerase	*Nocardiopsis aegyptia* sp. nov. DSM 44442^T^ (B)	Marine seashore sediments/culture	PHB, PHBV	30, 7	-, -	[393]
PHB depolymerase (33 kDa)	*Penicillium funiculosum* (F)	Culture	PHB	30, 7.5	-, 6.5	[394]
PHB depolymerase (36 kDa)	*Penicillium simplicissimum* LAR13 (F)	Soil/culture	PHB	25, 30, 37, -	45, 5.0	[395]
PHB depolymerase	*Paecilomyces lilacinus* D218 (F)	Soil/liquid culture	PHB, PCL	30, 6.0	50, 6.5–7.5	[365]
PHB depolymerase	*Pseudomonas fluorescens* (B), *Pseudomonas aeruginosa* (B), *Pseudomonas putida* (B)	Contaminated soil/culture	PHB, PHBV	30, 7.9	-, -	[116]
PHB depolymerase (48 kDa)	*Comamonas acidovorans* YM1609 (B)	Freshwater/culture	PHB, PHBV	37, 7.4	-, -	[396]
PHB depolymerase	*Pseudomonas stutzeri* (B)	Sea water/Buffer solution	PHB	30–45, 7.4	-, 7–7.5	[397]
PHB depolymerases (44, 46 kDa)	*Agrobacterium* sp. K-03 (B)	Culture	PHB, PHBV	30, 8	45, 7,9 and 8.1	[398]
PHB depolymerase (49 kDa)	*Streptomyces exfoliatus* K10 (B)	Culture	PHB	25–37, 8	40, 8.5–9	[399]
PHB depolymerase (40 kDa)	*Pseudomonas pickettii* (B)	Culture	PHB	37, 7.4	40, 5.5	[400]
PHB depolymerase (53 kDa)	*Comamonas* sp. (B)	Solid culture	PHB	37, 8	-, -	[401]
PHB depolymerase (65 kDa)	*Alcaligenes faecalis* AE122 (B)	Seawater/culture	PHB	37,	-, -	[402]
PHB depolymerase (95.5 kDa)	*Alcaligenes faecalis* AE122 (B)	Seawater/culture	PHB	30, 6.8–7.5	55, 9	[403]
PHB depolymerase (40 kDa)	*Aspergillus fumigatus* (F)	Culture	PHB	30–32, 8	-, -	[404]
PHB depolymerase (48 kDa)	*Alcaligenes faecalis* T_1_ (B)	Activated sludge/culture	PHB	30, 7.5	-, 7.5	[405]
PHB depolymerase	*Ralstonia pikettii* (B)	Culture	PHB, PHBV	20, 7.5	-, -	[278]
PHB depolymerase (45 kDa)	*Paecilomyces lilacinus* F4-5 (F)	Soil/culture	PHB, PHBV	27–37, 7	50, 7	[406]
PHB depolymerase (52.2 kDa)	*Diaphorobacter* sp. PCA039 (B)	Culture	PHB, PHBV	30, -	45, 8	[407]
PHB depolymerase (63.7 kDa)	*Aspergillus fumigatus* 202 (F)	Soil/culture	PHB	30, 37, 45, 7	45, 7	[408]
PHB depolymerase (20 kDa)	*Penicillium expansum* (F)	Wastewater/culture	PHB	30, 5	50, 5	[409]
PHB depolymerase	*Streptomyces* sp. SNG9 (B)	Marine/liquid culture	PHB, PHBV	30, 7	-, -	[410]
PHB depolymerase (45 kDa)	*Bacillus* (B), *Clostridium* (B), *Streptomyces* (B), *Alcaligenes* (B), *Comamonas* (B), *Pseudomonas* (B), *Zoogloea* (B)	Soil, lake water, activated sludge, air/liquid culture	PHB, PHV, PHBV	4–58, 4.8–10.6	29–35, 9.4	[411]
PHB depolymerase (37 kDa)	*Penicillium funiculosum* (F)	Culture	PHB	30, 5	-, 6	[412]
PHB depolymerase (48 kDa)	*Paecilomyces lilacinus* D218	Buffer solution	PHB, PHBV	30, 6.8	45, 7	[413]
PHB depolymerase	*Aspergillus clavatus* strain NKCM1003 (F)	Soil/culture	PES, PHB, PCL, PBS	30, -	-, -	[414]
PHBV depolymerase (36, 68, 72, 90 kDa)	*Aspergillus* sp. NA-25 (F)	Soil/solid culture	PHBV	30, 7.0	45, 7.0	[415]
PHBV depolymerase (43.4 kDa)	*Acidovorax* sp. HB01	Activated sludge/	PHBV, PHB, PCL	37, 6.8	50, 7	[416]
PHBV depolymerase (51 kDa)	*Streptomyces* sp. strain AF-111 (B)	Sewage sludge/culture	PHBV	30–37,	35–55, 7–8	[417]
PHV depolymerase (43.6 kDa)	*Pseudomonas lemoignei* (B)	Liquid culture	PHB, PHV	37, 8	-, -	[418,419]
Polyurethanase—lipase (28 kDa)	*Bacillus subtilis (B)*	Soil/liquid culture	PU	30, 7	-, -	[420]
Polyurethanase esterase (27 kDa)	*Pseudomonas chlororaphis* (B)	Liquid culture	PU	30, 7.2	-, 7–8	[421]
Polyurethanase esterase/protease (63 kDa), Polyurethanase esterase (31 kDa)	*Pseudomonas chlororaphis* (B)	Yeast extract salts medium	PU	30, -	-, 8.5 and 7	[422]
Polyurethanase protease (29 kDa)	*Pseudomonas fluorescens* (B)	Liquid culture	PU	30, 7.2	25, 5.0	[423]
Polyurethanase lipase	*Pseudomonas protegens* strain Pf-5 (B)	Liquid culture	PU	27, 7.4	-, -	[424]
Polyurethanase (66 kDa)	*Acinetobacter gerneri* P7 (B)	Liquid culture	PU	30, 7.0	37, 8.0	[425]
Polyurethanase—protease	*Alternaria solani* Ss1-3 (F)	Soil/liquid culture	PU	(20–35), (4.0–8.0)	30, 7.0	[426]
Polyurethanase—esterase and amidase	*Alicycliphilus* sp. BQ8 (B)	Liquid culture	PU	37, 7.0	-, -	[427]
Polyurethanase serine hydrolase family (21 kDa)	*Pseudomonas chlororaphis* (B), *Pestalotiopsis microspora* (E2712A, 3317B) (F), *Lasiodiplodia* sp. E2611A (F), *Bionectria* sp. strain E2910B (F), *Aspergillus niger* (F), *Pleosporales* sp. E2812A (F)	Soil/liquid culture	PU	30, -	-, -	[428]
Protease (3.4.21)	*Amycolatopsis orientalis* (A)	Liquid culture	PLLA	30–40, 7.0	-, -	[429]
Protease	*Bacillus licheniformis* (B)	Buffer solution	PLA	37, -	-, -	[266]
Protease	*Tritirachium album* (F), *Lentzea waywayandensis* (A), *Amycolatopsis orientalis* (A)	Culture	PLLA	30, 7	-, -	[430]
PLA-degrading enzyme closely related to Protease (40–42 kDa)	*Amycolatopsis* sp. strain 41 (A)	Soil/liquid culture	PLLA	37, 7.0	37– 45, 6.0	[431]
Protease, esterase, and lipase	*Amycolatopsis* sp. strain SCM_MK2-4 (A)	Soil/liquid, solid culture	PLA, PCL	30, 7.0	-, -	[432]
Protease, PLA-degrading enzyme	*Stenotrophomonas pavanii* CH1 (B), *Pseudomonas geniculata* WS3 (B)	Soil, wastewater sludge/liquid culture	PLA	30, -	30, 7.530, 8.0	[433]
Proteinase K (3.4.21.64)	-	Buffer solution	PLLA	37, 8.6	-, -	[187]
Proteinase K	-	Buffer solution	Amorphous PLLA (not crystalline PLLA)	37, 8.6	-, -	[344]
Proteinase K	*Tritirachium album*	Liquid culture	PLA	30, -	-, -	[317]
Proteinase K	-	Culture	PLLA, PES, PEA, PBS, PBSA, PCL	37, 7.0	-, -	[302]
Proteinase K	-	Culture	PLLA	37, 8.6.	-, -	[69,186]
Proteinase K	*Tritirachium album*	Buffer solution	PLA	37, -	-, -	[266]
(PVAase)-Cu_3_(PO_4_)_2_	*Bacillus niacini* (B)	Culture	PVOH	30, 8.0	30, 7	[434]
PVOH oxidase (1.1.3.30)	*Sphingomonas* sp. (B)	Activated sludge/culture	PVOH	25, 7.5	-, -	[435]
PVOH oxidase	*Sphingopyxis* sp. PVA3 (B)	Activated sludge/culture	PVOH	30, 7.2	-, -	[436]
PVOH-degrading enzyme (30 kDa)	*Pseudomonas* (B)	Buffer solution	PVOH	27, 7.3	40, 7–9	[437]
PVOH-degrading enzyme	*Streptomyces venezuelae* GY1	Culture	PVOH	30, 8	-, -	[438]
PVOH-degrading enzyme	*Penicillium* sp. WSH0-21 (F)	Activated sludge/culture	PVOH	30, 7	-, -	[439]
PVOH-degrading enzyme (67 kDa)	*Alcaligenes faecalis* KK314	River water/culture	PVOH	30, 7.2	-, -	[440]
Serine enzyme (3.4.21) (24 kDa)	*Amycolatopsis* sp. strain K104-1 (A)	Soil/liquid medium	PLLA	37, 7.0	55–60, 9.5	[441]
Subtilisin (3.4.21.62)	-	Culture	PLA, PEA, PBS, PBSA, PCL	37, 7.0	-, -	[302]
Trypsin (3.4.21.4)	-	Culture	PLA, PEA	37, 7.0	-, -	[302]
Aliphatic–aromatic co-polyester-degrading enzyme (27–31 kDa)	*Roseateles depolymerans* TB-87 (B)	Soil, fresh water/culture	PBS, PBSA, PCL, PBST, PES	20–40, 6–11	35, 7	[442,443]
Esterase and protease activity	*Paenibacillus amylolyticus* TB-13 (B)	Soil/culture	PLA, PBSA, PBS, PCL, PES	30, -	-, -	[444]
Esterase and amidase	-	Buffer solution	PU	37, 7	-, -	[445]
PU esterase (48 kDa)	*Pseudomonas fluorescens* (B)	Culture	PU	37, -	-, -	[446]
Lipase, manganese peroxidase, laccase	*Penicillium brevicompactum* OVR-5 (F)	Liquid medium	PVOH	28, -	30, 7	[447]
Fungal peroxidase (1.11.1.7), Laccase (1.10.3.2)	*Aspergillus* sp. (F)	Buffer solution	PU	30, 7	-, -	[448]
Esterase deacetylase (3.5.1.)	*Comamonas* sp. strain NyZ500	Activated sludge/culture	PVOH	37, -	-, -	[449]
-	*Pseudomonas aeruginosa* (B)	Culture	PU	37, -	-, -	[450]
-	*Nocardioides* OK12	Culture	PHB, PHBV	30, -	-, -	[451]
-	*Aspergillus flavus* (F)	Culture	PU	28, 6–6.5	-, -	[452]
-	*Aspergillus versicolor* (F)	Culture	PBSA	30, 7.2	-, -	[453]
-	*Pseudomonas chlororaphis* ATCC 55,729 (B)	Culture	PU (foam)	29, -	-, -	[454]
-	*Aspergillus fumigatus* (F), *Paecilomyces farinosus* (F), *Fusarium solani* (F), *Penicillium simplicissimum* (F), *Penicillium minioluteum* (F), *Penicillium pinophilum* (F), *Penicillium funiculosum* (F)	Activated sludge soil/farm soil	PHB	28, 37, -	-, -	[251]
-	*Pseudonocardia* sp. RM423 (A)	Culture	PLA	30, 7	-, -	[227]
-	*Fusarium solani* (F), *Candida ethanolica* (F)	Compost, Soil	PU	25, 45	-, -	[455]
-	*Enterobacter* sp. IBP-VN1 (B), *Bacillus* sp. IBP-VN2 (B), *Gracilibacillus* sp. IBP-VN3 (B), *Enterobacter* sp. IBP-VN4 (B), *Enterobacter* sp. IBP-VN5 (B), *Enterobacter* sp. IBP-VN6 (B)	Seawater/culture	PHB, PHBV	27.1–30.4, 7.0–7.5	-, -	[456]
-	*Acidovorax delafieldii* (B7-7, B7-21, B7-28) (B), *Streptomyces acidiscabies* A2–21 (A), *Streptomyces griseus* A2–10 (A), *Fusarium oxysporium* F1–3 (F), *Paecilomyces lilacinus* F4–5 (F), *Paecilomyces farinosus* F4–7 (F)	Natural Soil/incubated artificial soil	PHBV	30, -	-, -	[457]
-	*Pseudomonas aeruginosa* (B)	Soil/liquid culture	PDLA	37, -	-, -	[287]
-	*Fusarium solani* WF-6 (F)	Soil/culture	PBS	30, -	-, -	[458]
-	*Flammulina velutipes* (F)	Culture	PVOH	28, -	-, -	[459]
-	*Aspergillus flavus* (F), *Aspergillus oryzae* (F), *Aspergillus parasiticus* (F), *Aspergillus racemosus* spp. (F)	Soil/culture	PHB, PHBV	28–30, 6–7	-, -	[460]
-	*Azospirillum brasilense* BCRC 12,270 (B)	Liquid culture	PBSA	30, 7.0	-, -	[461]
-	*Aspergillus fumigatus* (F)	Compost/culture media	PCL	23, 25, 30, 37, 5.5	-, -	[183,462]
-	*Aspergillus fumigatus* (F) strain NKCM1706	Soil/culture	PBS, PBSA, PES, PHB, PCL	30, 7	30, -	[463]
-	*Leptothrix* sp. TB-71 (B)	Culture nutrient broth	PBST, PBAT	30, -	-, -	[464]
-	*Burkholderia cepacia* (B)	Culture	PLLA	35, 7	-, -	[465]
-	*Bacillus pumilus* strain 1-A (B)	Soil/Culture	PBSA, PBS, PCL	30, 7.0	-, -	[466]
-	*Bacillus* sp. JY14 (B)	Marine/culture	PHB, PHBV	30, -	-, -	[467]
-	*Pseudomonas* sp. (B)	Marine water/culture	PCL	25, -	-, -	[468]
-	*Actinomadura* AF-555 (A)	Soil/culture	PHBV	37, -	-, -	[277]
-	*Trichoderma viride* (F)	Soil/liquid culture	PLA	28, -	-, -	[469]
-	*Chryseobacterium* S1 (B), *Sphingobacterium* S2 (B), *Pseudomonas aeruginosa* (S3, S4) (B)	Compost/liquid culture	PLA	30, 7.2	-, -	[470]
-	*Amycolatopsis* sp. (SST, SNC, SO1.2, SO1.1) (A)	Soil/basal medium	PLLA	30, 7	-, -	[471]
-	*Amycolatopsis* sp. (A)	Culture	PLLA, PCL, PHB	30, 7.3	-, -	[472]
-	*Amycolatopsis* sp strain 3118 (A)	Soil/liquid medium	PLLA	(30, 37, 43, 48), 7.0	43, 7.0	[473]
-	*Amycolatopsis* sp. strain HT-32 (A)	Soil/liquid culture	PLLA	30, 7.0	-, -	[474]
-	*Amycolatopsis* sp. strain KT-s-9 (A)	Soil/liquid medium	PLLA	30, -	-, -	[475]
-	*Acidovorax facilis* (B), *Varivorax paradoxus* (B), *Pseudomonas syringae* (B), *Comamonas testosteroni* (B), *Cytophaga jhonsonae* (B), *Bacillus megaterium* (B), *Bacillus polymyxia* (B), Streptomyces spp. (B), *Aspergillus fumigatus* (F), *Paecilomyces marquandii* (F), *Penicillium daleae* (F), *Penicillium simplicissimum* (F), *Penicillium ochrochloron* (F), *Penicillium adametzii* (F), *Penicillium chermisimun* (F), *Penicillium restrictum* (F), *Acremonium* sp. (F)	Soil/incubated	PHB, PHBV	(15, 28, 40),(3.5, 3.9, 6.3, 6.5, 7.1)	-, -	[476]
-	*Acinetobacter calcoaceticus*, *Arthrobacter artocyaneus*, *Bacillus aerophilus*, *Bacillus megaterium*, *Bacillus* sp., *Brevibacillus agri*, *Brevibacillus invocatus*, *Chromobacterium violaceum*, *Cupriavidus gilardii*, *Mycobacterium fortuitum*, *Ochrobactrum anthropi*, *Staphylococcus arlettae*, *Staphylococcus haemoliticus*, *Staphylococcus pasteuri*, *Pseudomonas acephalitica*, *Rodococcus equi*, *Bacillus cereus*, *Bacillus megaterium*, *Bacillus mycoides*, *B. agri*, *Gordoniaterrari*, *Microbacterium paraoxydans*, *Burkholderia* sp, *Streptomyces*, *Mycobacterium* spp, *Nocardiopsis*, *Gongronella butleri*, *Penicillium*, *Acremonium recifei*, *Paecilomyces lilacinus*, *Trichoderma pseudokoningii*,	Soil	PHB, PHBV	(26–31), -	-, -	[477]
-	*Amycolatopsis thailandensis* strain CMU-PLA07^T^ (A)	Soil/liquid culture	PLLA	30, -	-, -	[478]
-	*Bacillus pumilus* B12 (B)	Soil/minimal salt medium agar	PLA	30, -	-, -	[479]
-	*Kibdelosporangium aridum* (B)	Solid/liquid culture	PLLA	30, 6.6–7.8	-, -	[480]
-	*Lentzea* (B), *Saccharothrix* (A), *Amycolaptosis* (B), *Kibdelosporangium* (B), *Streptoalloteichus* (B)	Culture	PLLA	30, 7	-, -	[481]
-	*Pseudonocardia alni* AS4.1531^T^ (A)	Soil	PLA	30, -	-, -	[482]
-	*Saccharothrix waywayandensis* (A)	Culture	PLLA	30, 7	-, -	[483]
-	*Tritirachium album* ATCC 22,563 (F)	Liquid culture with gelatin	PLLA	30, -	-, -	[484]
-	*Parengyodontium* (F), *Aspergillus* (F), *Penicillium* (F), *Fusarium* (F)	Soil/agar medium	PLLA, PCL	25, 7.0, 6.0	-, -	[485]
-	*Stenotrophomonas maltophilia* LB 2-3 (B)	Compost/Sturm test	PLLA exposed to UV irradiation	37, 7	-, -	[72]
-	*Mortierella* sp. (F), *Doratomyces microsporus* (F), *Fusarium solani* (F), *Fennellomyces* sp. (F), *Aspergillus fumigatus* (F), *Verticillium* sp. (F), *Lecanicillium saksenae* (F), *Cladosporium* sp. (F), *Trichoderma* sp. (F)	Compost, soil	PLLA	25, 7.2	-, -	[486]
-	*Bordetella petrii* PLA-3 (B)	Compost	PLLA	30, 37, 7.0	-, -	[248]
-	*Flammulina velutipes* (F)	Quartz sand/culture	PVOH	28, -	-, -	[459]
-	*Bacillus cereus* RA 23 (B)	Oil sludge/culture	PVOH	30, 7.0	28, 7	[487]
-	*Bacillus* sp. (B), *Curtobacterium* sp. (B)	Sewage sludge/culture	PVOH	35, 8.0	-, -	[488]
-	*Eutypella* sp. BJ (F)	Soil compost/culture	PVOH	30, -	-, -	[489]
-	*Geomyces pannorum* (F), *Phoma* sp. (F)	Soil/solid culture	PU	<25, 5.5, 6.7	-, -	[490]
-	*Geomyces* sp. B10I (F), *Fusarium* sp. B3′M (F), *Sclerotinia* sp. B11IV (F)	Antarctic soil/liquid culture	PCL, PBS	(14, 20, 28), -	-, -	[290]

* Enzymes and microorganism name(s) as reported in publication. A, actinomycetes; B, bacteria; F, fungi; Y, yeast.

### 7.2. Extracellular Enzymes

**Figure 13:**
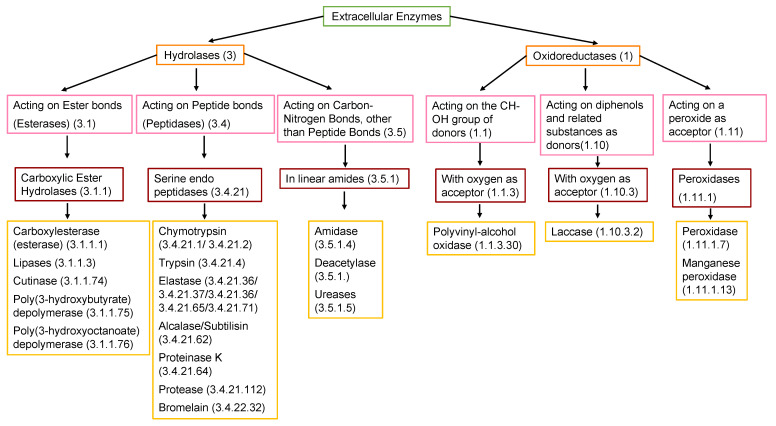
Classification of the main extracellular enzymes reported for enzymatic activity of aliphatic and aliphatic/aromatic polyesters, poly(urethanes) (PUs) derived from esters, and poly(vinyl alcohol) (PVOH). The two main groups of extracellular enzymes involved in the enzymatic activity are hydrolases (EC 3) and oxidoreductases (EC 1). Main enzymes are further classified into different groups depending on their action toward specific groups. The numbers in parentheses are the enzyme codes according to the Enzyme Commission (EC) nomenclature [112].

Figure 13 depicts the main extracellular enzymes reported for the depolymerization of aliphatic and aliphatic/aromatic polyesters, poly(urethanes)s (PUs) derived from ester, where the ester bond cleavage is considered as the rate-determining step [115], and PVOH. These enzymes belong to the esterase (EC 3.1) and peptidase (EC 3.4) groups of the main group hydrolases (EC 3); and oxidoreductases (EC 1).

Enzymes such as cutinases, esterases, lipases, and PHA/PHB depolymerases are the main extracellular enzymes for enzymatic hydrolysis of the ester group and belong to the α/β hydrolase family that are structurally similar but with diverse functionality [178,491,492]. The natural activity of the esterase group of enzymes is the hydrolysis of lipids. Proteases are the main group for enzymatic degradation of the peptidase group. For polyurethanes, various esterases, proteases, amidases (EC 3.5.1.4), and ureases (EC 3.5.1.5) also have been reported to induce enzymatic degradation. In this case, esterases are involved in ester scission, and ureases are more inclined to the scission of urethane bonds and are more resistant to chemical and enzymatic hydrolysis [111]. In the case of PVOH, and some PU, an oxidative pathway prior to the hydrolytic enzymatic degradation has been reported, and the main extracellular enzymes are the oxidoreductases (EC 1).

#### 7.2.1. Carboxylesterases

In general, carboxylesterases (3.1.1.1) are reported as esterases, creating some confusion in the literature, since the main classification group (3.1) is esterases. Since the natural function of esterases is the hydrolysis of lipids, for polymer attack, they need a hydrophobic surface to be activated for the scission of ester bonds. Carboxylesterases, in general, act hydrolyzing short-chains (C < 10) and present a lid domain that covers the active site. The lid domain (present also in lipases), when binding to the hydrophobic substrate, opens the active site to promote the catalysis. The lid domain structure is important since some differences can determine the specificity of the enzymes toward some substrates. Disulfide bonds are not present in carboxylesterases [493]. Hajighasemi et al. reported the action of carboxylesterases from *Alcanivorax borkumensis* and *Rhodopseudomonas palustris* on PLA and other polyesters; the enzymatic endo and exo activity resulted in the production of oligomers, dimers, and monomers of PDLLA but did not show activity for PDLA or PLLA [301].

#### 7.2.2. Lipases

Lipases (3.1.1.3) are water-soluble extracellular enzymes reported to show enzymatic hydrolytic activity for several biodegradable polymers such as PLA (PLLA and PDLA), PCL, PBS, PBSA, PBAT, PBA, PEA, and PU esters (Table 8). The typical structure of lipases is a protein structure covered by a lid-like structure. Like carboxylesterases, lipases need a hydrophobic surface to be activated, since its natural function is the hydrolysis of lipids; increased lipase activity is observed when a hydrophobic substrate starts to form an emulsion due to its contact with a hydrophilic aqueous medium [491,494]. However, the difference with respect to carboxylesterases is that lipases prefer to break down long chains (C > 10). Lipases are unable to hydrolyze ester bonds in intermediates that become water soluble [123,178]. However, Rizzarelli et al. reported some ability of lipases to hydrolyze dissolved esters in water solution [495]. Such findings indicate that the nature of the polymer could be more important than the stereo chemistry in the vicinity of the ester bond for substrate preference by enzymes with lid-like structures, such as lipases. In addition, some works have reported that lipases act preferentially by random chain scission, showing an endo-type behavior where *M_w_* reduction is highly affected in comparison to end chain scission [496].

In the case of lipases, the active site is found in a deep cavity of the protein structure. This is shielded by a lid-like α-helical structure that is reoriented when in contact with the substrate. The degree of freedom of polymer chains to move is a key factor in controlling the hydrolytic depolymerization of polyesters. This mobility ensures that the polymer chain can fold itself and fit in the active site of the lipase enzyme to carry out the depolymerization [491]. Hence, polyesters must be mobile enough to reach the active site of the lipase, making thermal and conformational properties key factors for depolymerization, since exposure to temperature controls the mobility of polymer chains [178]. In general, lipases require a hydrophobic surface to reach full hydrolytic activity. For this reason, lipases are not likely to be observed developing high enzymatic activity for the PHAs family of aliphatic polyesters. The *M_w_* of lipases from bacteria has been reported in the range of 20 to 77 kDa [491]; thus, their size allows activity on the surface of polymers.

#### 7.2.3. Cutinases

Cutinases (3.1.1.74) are hydrolytic enzymes considered the smallest members of the α/β hydrolase superfamily (20–25 kDa) [497,498]. Enzymatic activity for cutinases has been reported for several biodegradable polymers. Cutinases are mainly produced and released by fungal pathogens and can degrade the polyester cutin, which is a natural crosslinked lipid polymer composed of *n*-C_17_ and *n*-C_18_ hydroxy and epoxy fatty acids, present in plant cell walls and insoluble in water. However, some bacteria can also produce cutinases [499]. Cutinases are able to show enzymatic activity without needing interfacial activation like lipases and are capable of being active in both soluble and emulsified substrates [499], which is primarily due to the absence of the hydrophobic lid that covers the active site. Furthermore, the active site of cutinases is considered large enough to locate and catalyze even high *M_w_* polyesters [500]. As shown in Table 8, cutinases act mostly against aliphatic polyesters; however, results for aliphatic–aromatic polyesters are scarce. A comparative study of five extracellular cutinases released by five species of microorganisms found that some cutinases are more stable and have higher activity toward polymer substrates than others; the higher stability and efficiency were related to additional disulfide bond formation [303]. In general, the presence of covalent disulfide bonds and neutral charge in the crowning area of the active site provides extra stability to the tertiary structure by linking regions of proteins. The presence of disulfide bonds in a cutinase was also reported by Liu et al. [314], and together with a favored catalytic triad, it resulted in improved activity, enhanced thermostability, and higher activity toward PCL. A cutinase (21.6 kDa) from the fungus *Aspergillus oryzae* was able to degrade PBS and PBSA and also showed low activity for PLA [188]. Furthermore, PCL was reported to be an optimal substrate for cutinases [312].

A study on the effect of pH on the surface charge of the area around the active site of cutinases reported that the active site becomes more positive as pH decreases from alkaline to acidic values, resulting in lower activity toward polymers such as PCL [303]. Electrostatic surface potentials generated by charged residues affect the enzyme/substrate interaction, transition stage stabilization, and efficiency during the product release stage [303]. Similar results were reported from the interaction of cutinase and PBS; the release of acidic monomers from PBS affected the pH and the activity of the cutinase, lowering the degradation rate of the PBS films [305,308]. The presence of the cofactors Ca^2+^, Na^+^, and K^+^ increased the activity of cutinases toward polymers such as PCL, PBS, and PBSA; however, the cofactors Mg^2+^ or Zn^2+^ did not show a significant effect or significantly inhibited the activity of the enzymes [304,306,307].

#### 7.2.4. PHA, and PHB Depolymerases

PHA and PHB depolymerases (3.1.1.75 and 3.1.1.76) are produced by microorganisms and accumulate within the cells as intracellular carbon and energy storage. Thus, they can undergo enzymatic degradation by functioning intra or/and extracellular. PHB depolymerases (3.1.1.75) show activity against short-chain length PHAs as PHB, PHV, and PHBV, while others (3.1.1.76) show more depolymerization activity on medium-chain length PHAs [498]. The primary structure of PHA depolymerases is formed by two functional domains, a catalytic domain, and a substrate-binding domain; and it is activated by the presence of Ca^+2^ and Mg^+2^ or inhibited by Cu^+2^, Fe^+2^, Mn^+2^, and Hg^+2^ [498]. The inhibition of enzymatic hydrolysis due to the presence of detergents highlights the likely presence of a hydrophobic region near the active site of PHA depolymerases [498]. Furthermore, PHA depolymerases are reported to have exo and endo behavior, since they were able to release monomers and oligomers [498]. The extended presence of hyphae due to the colonization of fungi on the PHBV surface has been reported as evidence of enzymatic degradation by extracellular PHAs depolymerases released by fungus [457]. In terms of the structure, some PHA depolymerases are reported to belong to the serine esterases group due to the presence of lipase boxes [395]. Even though PHA depolymerases are specific for PHAs, enzymatic activity has been reported also for other polyesters (Table 8).

The presence of additional carbon sources may reduce the enzymatic activity against polymers. For example, the reduction in PHA depolymerase produced by *Aspergillus* sp. showed a repression apparently influenced by the type of carbon source added to the media, which was indicative of a regulated behavior as a function of the available carbon source [460]. This finding is in accordance with the hypothesis that when abundant labile nutrients are present, the decomposition of more recalcitrant compounds is inhibited [119].

#### 7.2.5. Peptidases (Proteinase K and Protease)

Peptidases (EC 3.4), a group of enzymes acting on peptide bonds, are also commonly called proteases, generating some confusion in the literature. Peptidases or proteases hydrolyze peptide bonds that link amino acids in a protein. For example, Proteinase K (3.4.21.64) and proteases (3.4.21.112) belong to the serine endo peptidases (3.4.21), which are enzymes that preferentially catalyze bond scission in the middle of the substrate chain. In addition, it has been reported that enzymes belonging to the serine endo peptidases are able to hydrolyze polyesters such as PLA. Proteinase K and proteases have been identified for major enzymatic activity on PLA. Lim et al. [302] reported the ability of Proteinase K to depolymerize PES, PEA, PBS, PBSA, and PCL but at lower levels of enzymatic activity than on PLA. More specifically, Proteinase K showed a higher enzymatic activity for PLLA (amorphous preferentially) than for PDLA and PDLLA. 

The activity of these classes of enzymes toward PLA is still not fully understood in the sense that these enzymes are more prone to attack the scission of peptide bonds. Tokiwa and Jarerat [501] concluded that enzymes showing activity on PLA belong to the peptidases or protease-type group, and these enzymes are able to recognize the repeated l-lactic acid unit of PLA as the natural homologue l-alanine unit of silk fibroin, which is a natural protein present in silk. Later work by Lim et al. [302] reported the enzymatic ability of serine proteases on PLA, PHB, PES, PEA, PBS, PBSA, and PCL; in particular, alpha-chymotrypsin, a mammalian enzyme, showed preferential activity for PLA. In studies on PLA biodegradability, the incorporation of agents to the media, such as silk fibroin or gelatin, as a nitrogen source to induce the production of protease, has resulted in increased enzymatic activity for PLA, since proteases are more prone to interact with peptide bonds [433].

#### 7.2.6. Amidases and Ureases

In addition to esterases and proteases, PUs derived from esters can be enzymatically degraded by amidases (3.5.1.4) and ureases (3.5.1.5). Amidases attack the amide groups, proteases can attack amide and urethane bonds, esterases attack the ester bonds, and ureases catalyze the hydrolysis attack of the urea groups. However, the information in terms of amidases and ureases showing enzymatic activity toward PUs derived from esters in mesophilic environments is scarce [502,503,504,505].

#### 7.2.7. Oxidoreductases PU and PVOH-Oxidases

Oxidoreductases (EC 1) have shown activity for PVOH and PU within the groups EC 1.1, EC 1.10, and EC 1.11. More specifically 1.1.3 (with O_2_ as electron acceptor), polyvinyl-alcohol oxidase (1.1.3.30) or dehydrogenase have shown enzymatic activity on PVOH; these are enzymes that can act extra or intracellular. Laccase (EC 1.10.3.2) has been reported to show enzymatic activity against both PVOH and PU (Table 8).

### 7.3. Biosurfactants and Synthetic Surfactants

Biosurfactants are amphipathic molecules with the capacity of reducing surface and interfacial tension between liquids, solids, and gases. They contain both hydrophilic and hydrophobic moieties that can improve the interaction between phases of different degrees of polarity and hydrogen bonding [506,507]. Microorganisms are capable of synthesizing and releasing biosurfactants such as glycolipids and phospholipids to emulsify the substrate and stimulate other functions as extracellular enzymatic activity [508].

Hydrophobins are a type of amphipathic surfactant secreted by fungi microorganisms; besides other functions, they attach to the surface of biodegradable polymers and stimulate the hydrolysis by extracellular enzymes. They present a dual behavior with hydrophobic and hydrophilic parts (amphipathic proteins), and they are adsorbed to the surface of the polymer, condensing and stimulating its enzymatic hydrolysis by recruiting extracellular enzymes [509,510]. Hydrophobins are important to support the growth of fungal aerial structures (hyphae) and conidiospores by playing an important role for fungal adhesion to hydrophobic surfaces, the development of a protective surface coating, and reduction in water tension [509,510].

Synthetic commercial surfactants are widely used during studies of extracellular enzymatic activity on polymers and are classified according to the nature of their polar grouping. Researchers have reported on the use of ionic and nonionic surfactants. Some common synthetic nonionic surfactants are added to culture studies for emulsification, including commercials ones such as Polysorbate 80 and polyoxyethylene type, to increase microbial activity on the polymer surface by increasing the hydrophilicity of the surface [121].

The interaction between surfactants and enzymes is still a subject of exploration. Holmberg mentioned that probably nonionic surfactants are more benign than ionic surfactants. The way in which ionic surfactants interact with enzymes can introduce significant changes in the conformational structure of the enzyme [511]. Detailed discussion about bio and commercial surfactants can be found elsewhere [506,507,511,512].

## 8. Polymers Susceptible to Biodegradation

**Figure 14:**
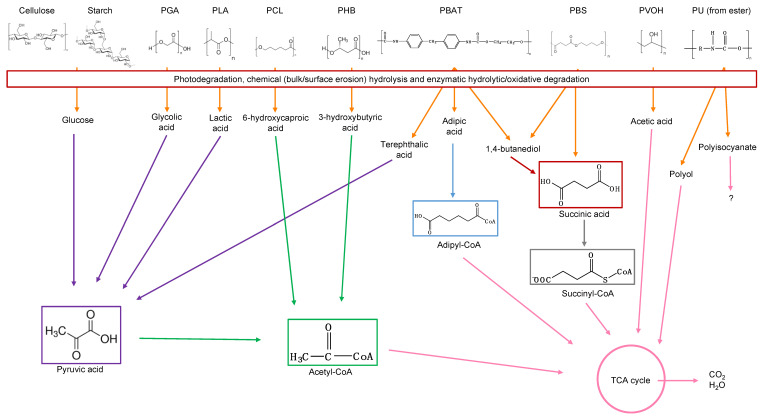
Main tentative abiotic and biotic pathways for biodegradable polymers cellulose, starch, poly(glycolic acid) (PGA), poly(lactic acid) (PLA), poly(caprolactone) (PCL), poly(hydroxybutyrate) (PHB), poly(butylene adipate-*co*-terephthalate) (PBAT), poly(butylene succinate) (PBS), poly(vinyl alcohol) (PVOH), and poly(urethane) (PU) in aerobic conditions. Different mechanisms such as photodegradation, chemical hydrolysis, enzymatic degradation, and oxidative degradation breakdown the polymer chains into small fragments for microbial assimilation. The monomer constituents for different polymers once small enough diffuse through the cell wall and further break down through a series of β-oxidation and tricarboxylic acid (TCA) cycles to produce energy for metabolic processes under aerobic conditions while releasing CO_2_ and water.

The main group of biodegradable polymers susceptible to biodegradation in the mesophilic range are the aliphatic and aliphatic–aromatic polyesters; besides that, PVOH and the soft segment of PUs derived from esters are also considered biodegradable to some extent. Commercialized cellulose and starch-derived polymers, which are bio-based and naturally biodegradable, are also important to consider when discussing biodegradable polymers. Figure 14 shows the tentative pathways of the most common bio- or fossil-based polymers reported to undergo depolymerization by specific microorganisms and enzymes (Table 8); and reported to undergo mineralization in the mesophilic environments. The behaviors of these polymers in mesophilic environments are reviewed individually in this section.

For biodegradable polymers, complete degradation results from the action of both abiotic and biotic factors acting upon the polymer structure and affecting the degradation mechanisms. Degradation mechanisms can vary from abiotic (mechanical, thermal, photo, and/or chemical hydrolysis) to biotic (enzymatic activity and microbial assimilation), bringing both irreversible physical and chemical changes to the polymer structure [99].

### 8.1. Cellulose

Cellulose is a linear homopolymer of D-glucose units joined by β-1,4 glycosidic linkages, with a degree of polymerization ranging from several hundreds to over 10,000 [513]. Each glucose molecule is upside down in relation to the neighboring glucose molecule so that the repeating unit is cellobiose, consisting of two glucose molecules linked by a β-1,4 glycosidic bond. The fibrils of cellulose can have crystalline and amorphous regions. Depending on the origin and treatment, the crystallinity of cellulose can vary from fully amorphous to fully crystalline. Higher crystallinity makes cellulose resistant to chemical attacks. In the secondary wall of plant cells, cellulose forms several sheets organized as parallel microfibrils. These microfibrils are embedded in the matrix of hemicellulose and lignin. Pure cellulose is available in several forms, such as cotton and filter paper. Before 1950, cellulose-based polymers were one of the most important groups of polymers. Cellulose nitrate, the oldest plastic, was produced by replacing nitrates on all three hydroxyl groups of the cellulose glucose units. Several other cellulose ether and ester thermoplastics, such as cellulose acetate and cellulose butyrate, have been produced through the years [514]. The main cellulose ethers are: methylcellulose (MC)—non-thermoplastic, water-soluble with high O_2_ barrier, generally used as filler and thickener agent; carboxymethyl cellulose (CMC)—hydrophilic, non-thermoplastic, generally used as a viscosity modifier and thickener; hydroxypropyl cellulose (HPC)—thermoplastic, with water barrier and grease resistance, generally used for coatings, and as binder and thickener; and hydroxypropyl methyl cellulose (HPMC)—non-thermoplastic, non-heat-sealable, generally used for coating purposes [515,516,517,518]. Cellulose esters are thermoplastic, and they are produced by the reaction of organic or inorganic acid substituting the hydroxyls of the glucose unit. The prominent cellulose esters include cellulose acetate (CA), which is thermoplastic, used for molding and extrusion, and can exist in several forms such as cellulose acetate butyrate (CAB), cellulose acetate propionate (CAP), and cellulose triacetate (CTA) [514].

Cellulolytic and non-cellulolytic mixed populations of microorganisms are present where cellulosic waste is present. These microorganisms interact synergistically to complete the biodegradation of cellulose, which is ultimately converted to CO_2_ and H_2_O in aerobic environments through the pathways shown in Figure 14.

**Figure 15:**
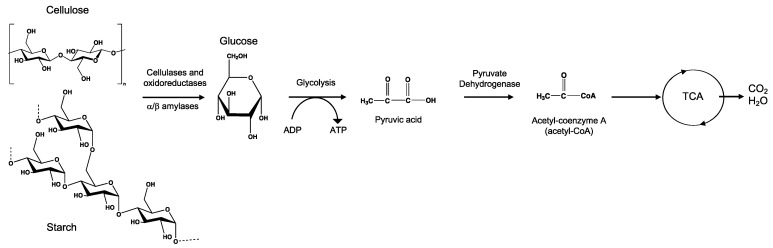
Pathway for enzymatic degradation, bioassimilation and mineralization of the natural polymer cellulose and starch. Cellulose and starch are enzymatically hydrolyzed by extracellular enzymes, cellulases and α/β amylases, respectively, to glucose. The soluble product glucose is converted to pyruvic acid via glycolysis. Pyruvate dehydrogenase catalyzes the conversion of pyruvic acid to acetyl-CoA, which is later metabolized through the tricarboxylic acid (TCA) cycle to produce energy and release CO_2_ and water.

Cellulose, as well as starch, is enzymatically hydrolyzed to glucose by extracellular enzymes, which are produced by bacteria and fungi (Figure 15). Natural polymers, such as cellulose and starch, are mostly attacked for enzymatic hydrolysis by cellulases and α/β amylases. In addition, oxidoreductases have been identified that can act prior to hydrolytic enzymes on cellulose [519].

**Figure 16:**
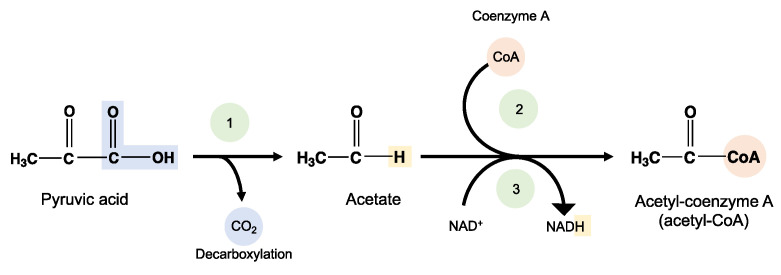
Pyruvic acid to Acetyl-CoA reaction pathway. (1) A carboxyl group is removed from pyruvic acid as a carbon dioxide molecule, and the resulting acetate is oxidized to acetyl. (2) Acetyl is attached to coenzyme A (CoA) to form acetyl CoA. (3) Electrons are given and taken up by NAD^+^ to form NADH. Adapted from [117].

After glucose is produced, glycolysis converts the glucose to pyruvic acid, which acts as the precursor for the TCA cycle. Glucose, together with the adenosine triphosphate (ATP), which is the molecule providing the energy source in the cell plus NAD^+^, and inorganic phosphate, breaks down into two pyruvates. In the pyruvic acid cycle, Figure 16, three main steps take place. First, a carbonyl group is removed from pyruvic acid, releasing CO_2_ to the surrounding media, resulting in a two-carbon hydroxyethyl group bound to the enzyme pyruvate dehydrogenase. Second, the hydroxyethyl group is oxidized to an acetyl group, and the electrons are picked up by the NAD^+^ (nicotinamide adenine dinucleotide), forming NADH. This electron will later be used by the cell to create energy through the ATP process. Third, the enzyme bound to the acetyl group is transferred to CoA, producing a molecule of acetyl CoA. This molecule is then further converted through the TCA cycle [117].

Cellulose biodegradation occurs primarily by cellulolytic microorganisms belonging to the bacteria and fungi. The aerobic biodegradation of cellulose occurs mostly by cellulolytic bacteria; several species identified in the genera *Cellulomona*, *Pseudomona*, *Thermomonospora*, and *Microbispora* have been shown to biodegrade cellulose [520].

Cellulose undergoes biodegradation in several environments. Amorphous forms of cellulose are used as positive controls for biodegradation studies due to their negligible chemical hydrolysis and rapid enzymatic hydrolysis rates and assimilation by microorganisms. In thermophilic and mesophilic environments, such as industrial composting or soil biodegradation, cellulose is widely used as a positive control, as stated in ASTM and ISO standards (Table 6). In marine environments, the mineralization of cellulose powder was reported to reach ≈95% after 450 days of testing at 25 °C [240], which is indicative of its high biodegradability in aquatic environments. Anunciado et al. [236] used cellulose in the form of a mulch paper, instead of powder, as a positive control in soil and composting conditions; after 365 days of testing mineralization, values were in the range of 50 to 80% for samples in soil at 27 °C.

### 8.2. Starch

Low-cost starch, mainly obtained from crops not intended for human consumption, is a bio-based material that can be blended with other polymers to produce novel bio-based and biodegradable blends. Starch consists of two main molecules making up the constitutional unit: amylose (linear) and amylopectin (branched). Starches with high amylose content have been used to produce suitable blends and to improve the thermal, mechanical, and gas barrier properties of the resulting blends [521,522,523,524,525]. The *T_g_* of pure starch is above its decomposition temperature, meaning the material does not soften and flow. To make it processable, starch needs to be combined with plasticizers such as glycerol, poly(ethylene glycol) (PEG), or sorbitol to obtain thermoplastic starch (TPS). The starch granules are plasticized by using plasticizers under heating, which provides a viscous melt that can then be processed using traditional methods such as extrusion foaming and injection molding [526]. TPS is highly hydrophilic, resulting in the leaching of plasticizer during storage and poor dimensional stability and mechanical properties with time [527]. However, TPS can be used to blend with other bio-based polymers, improving O_2_ barrier and elongation at break due to the presence of glycerol [523]. Since the properties of TPS by itself are not sufficient for producing polymeric structures for some applications, the possibility of blending TPS with other polymers to improve its mechanical and water barrier properties has opened a wide field for the development of novel TPS blends, with reactive functionalization as one of the suitable methods to enhance the compatibilization of TPS [525].

Since starch is sensitive to water, starch or the portion of blends containing TPS will mostly hydrolyze by enzymatic hydrolysis to glucose. The main extracellular enzymes involved during the enzymatic degradation of starches are α/β-amylases (Figure 14). The general pathways for the biodegradation and bioassimilation/mineralization of starch are shown in Figure 15 and Figure 16, respectively.

Starch, TPS, or TPS blends with other biodegradable polymers have shown high production of CO_2_, which is indicative of the high biodegradability of TPS even in mesophilic environments. Ho and Pometto [246] reported values of mineralization of ≈70% for starch at 28 at 40 °C in a soil environment under laboratory conditions after 180 days of testing. The main characteristic was rapid initial degradation at 40 °C, with a negligible abiotic phase of degradation, reaching the plateau stage at around day 60; lower activity was observed at 28 °C, reaching the plateau stage at around day 100.

### 8.3. Poly(Glycolic Acid)—PGA

PGA, the simplest aliphatic polyester, is a biodegradable and biocompatible thermoplastic that has been extensively used for many decades in the medical field for implants [528]. PGA can be synthesized using several mechanisms. The direct polycondensation polymerization of glycolic acid results in low *M_w_* PGA (*M_w_* < 50 kDa). The ring-opening polymerization of glycolic acid results in high *M_w_* PGA (*M_w_* >50 kDa). Solid-state polycondensation is used to increase the *M_w_* by increasing the polymer chain lengths in the absence of heat and O_2_ by constant removal of by-products using inert gas or under vacuum [529,530].

PGA has a *T*_g_ in the range of 35 to 40 °C and *T*_m_ between 220 and 230 °C (Table 4) [531]. PGA displays good gas barrier properties due to its crystalline and stereochemistry structure [528]. PGA is also resistant to most organic solvents. In addition, the high density of c. 1.53 g/cm^3^ awards PGA good mechanical properties compared with other biodegradable polymers; however, the high cost associated with the PGA production process has hampered its entry into the consumer market compared to other biodegradable polymers [528]. In general, PGA is blended with other polymers to improve their properties. For example, when PGA is blended with PLA, the result is better mechanical properties and improved flexural modulus of the PLA/PGA blend [532]. Due to its high O_2_ and H_2_O barrier properties, PGA can be used in packaging of products sensitive to O_2_ [533]. PGA is widely used in biomedical applications such as sutures, drug delivery, and tissue engineering [534].

**Figure 17:**
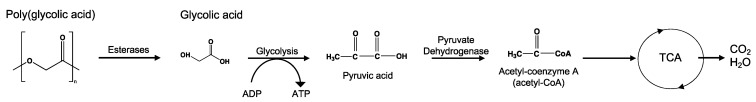
Biodegradation pathway for poly(glycolic acid) (PGA) in aerobic conditions. PGA is enzymatically hydrolyzed by extracellular enzyme, esterase to glycolic acid. Glycolic acid is converted to pyruvic acid via glycolysis. Pyruvate dehydrogenase catalyzes the conversion of pyruvic acid to acetyl-CoA, which is later metabolized through the tricarboxylic acid (TCA) cycle to produce energy and releasing CO_2_ and water.

PGA degradation starts by abiotic degradation, and chemical hydrolysis is by a non-specific chain scission of the ester backbone, with bulk erosion as the dominant mechanism (Figure 14) [44,528]. Therefore, water diffusion activated by temperature plays a crucial role in the initial hydrolysis of the ester backbone. The absence of asymmetrical methyl groups turns PGA more hydrophilic than PLA, increasing its bulk chemical hydrolysis rate. Currently, there is limited published information on PGA depolymerization by enzymatic activity in the mesophilic range in open environments. Since PGA has been used mainly for biomedical applications, most of the biodegradation data are from in vivo studies at 37 °C. Extracellular enzymes such as esterases have been reported to have enzymatic activity on PGA sutures [319]. After the initial chemical and enzymatic hydrolysis, PGA is degraded into small oligomers and glycolic acid, which can be bioassimilated and oxidized to become a substrate for the TCA cycle, as shown in Figure 17.

In terms of CO_2_ evolution and mineralization studies, the biodegradation of PGA in a marine environment at around 30 °C, which is high for marine environments, showed a longer lag phase than for cellulose, but ≈75% mineralization was reached at 28 days for both PGA and cellulose [528]. At thermophilic conditions in a simulated industrial composting environment at 58 °C, PGA showed lower mineralization than cellulose; 70% mineralization was reached at around day 40 for cellulose and at around day 70 for PGA [528].

### 8.4. Poly(Lactic Acid)—PLA

PLA, a biodegradable aliphatic polyester, is a widely used alternative for conventional fossil-based plastics. In addition to PLA being biocompatible and biodegradable (compostable), its production from renewable resources results in energy savings and lower greenhouse gas (GHG) emissions [535]. The building block for PLA is lactic acid or lactide, which is derived from the fermentation of glucose obtained from varied sources such as corn and sugar cane. Lactic acid has two enantiomers: l-lactic and d-lactic acid [536]. Lactide can be produced in three stereochemical configurations: l, l-lactide; l, d-lactide, and d, d-lactide. High *M_w_* PLA is obtained by ring-opening polymerization of the different lactides and polycondensation of low *M_w_* lactic acid [537,538]. PLA presents acceptable thermal, mechanical and barrier properties, and its main applications include food and medical product packaging, medical devices, fibers, textiles, plasticulture, and automotive parts [537]. The ratio of l-lactic and d-lactic acid in a final PLA formulation plays a crucial role in its final properties and degradation rate [537,538,539].

**Figure 18:**
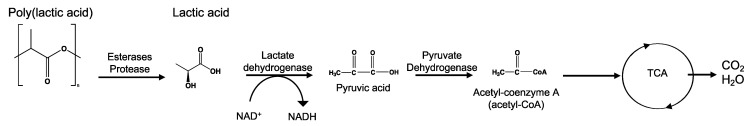
Biodegradation pathway for poly(lactic acid) (PLA) in aerobic conditions. PLA is enzymatically hydrolyzed by extracellular enzymes, namely esterases and proteases to lactic acid. Lactic acid monomers are converted to pyruvic acid. Lactate dehydrogenase catalyzes the conversion of pyruvic acid to acetyl-CoA, which is later metabolized through the tricarboxylic acid (TCA) cycle to produce energy and release CO_2_ and water.

The hydrolysable ester bonds in the backbone of the PLA structure (Table 4) make it susceptible to chemical hydrolysis. The chemical hydrolysis can proceed via bulk or surface erosion. Several mechanistic, phenomenological, and probabilistic models have been developed for PLA and can be extended to other aliphatic polyesters, explaining how diffusion and geometric properties can modify the pathways and incentivize one or the other mechanism [89]. The mechanism proceeds in different stages, starting with water diffusion into the material, followed by the degradation of amorphous regions. After degradation of the amorphous regions, the random chain scission and cleavage of ester bonds results in the release of soluble oligomers and monomers [77], which can be used as substrates for bioassimilation (Figure 18). The hydrolysis rate of PLA, as well as other polyesters, is highly dependent on temperature (below or above *T_g_*), pH, and several other properties of the polymer such as *M_w_* and crystallinity, as reviewed elsewhere [78]. In the absence of other factors accelerating other mechanisms, chemical hydrolysis is the most important mechanism in the mesophilic range for the abiotic process.

The degradation activity of PLA by microorganisms has been monitored by different methods and correlated to different biodegradation stages. The crystal structure change or biofilm formation for PLA degraded in a compost environment was observed using SEM [248]. Weight loss indicating the depolymerization of PLA was measured by size exclusion chromatography [433], the degree of biofragmentation of PLA fibers was monitored by X-ray diffraction (XRD) [299], and the generation of lactic acid was detected using an enzymatic bioanalysis kit [484].

The biotic degradation stage implies enzymatic activity and microbial assimilation. The enzymatic degradation of PLA involves interaction of the polymer with a reagent, such as water, in the hydrolysis reaction. Hydrolases, such as proteases and esterases, catalyze the hydrolysis reactions. When lactic acid becomes available for bioassimilation, it is transported through the semi-permeable membrane and is oxidized to pyruvic acid through a dehydrogenization reaction, which then follows the pyruvic acid pathway (Figure 18), as previously described.

Various bacteria, fungi, and actinomycetes strains have been identified as having some ability to degrade PLA in different forms such as pellet, film, powder, and sheet. These microorganisms were isolated from different environments, such as soil, compost, and wastewater sludge, by enrichment culture media, while some were procured from research facilities, as shown in Table 8. The extracellular enzymes secreted by these microorganisms have been reported to preferentially degrade the amorphous regions of PLA, since the backbone chains are highly disordered and have higher mobility as compared to the crystalline region. This flexibility and mobility aids in the binding of the backbone chain to ensure a fit into the active site of the enzyme [332]. The extracellular enzymatic activity efficiency is dependent on the type of PLA (PLLA, PDLA, or PDLLA) as well as the temperature, crystallinity, and *M_w_* of the PLA [248,540].

The enzymatic degradation of PLA involves the hydrolases with esterases (3.1) and peptidases (3.4) as the main groups of enzymes. Carboxyl esterases ABO2449 and RPA1511 (3.1.1.1) have been reported to hydrolyze PLLA and PDLA (Table 8) with the highest activity for ABO2449 in the range of 30 to 37 °C and for RPA1511 in the range of 55 to 60 °C [301]. The analysis of the hydrolysis suggested that, similar to other hydrolases (e.g., nucleases and proteases) that are active in depolymerizing polymeric substrates, these enzymes can exhibit both exo- and endo-esterase types of cleavage [301]. Table 8 also lists several esterases (3.1) able to degrade PLA such as lipases, cutinases, and carboxyl esterases. Peptidases (3.4) have been reported to be able to degrade PLA in culture media. For example, Proteinase K (3.4.21.64) has been shown to be efficient during the scission of polymer chains, favoring the hydrolysis of the amorphous region of PLLA [541]. The enzymatic degradation of PLA revealed the preferential activity of proteases for PLLA and for PDLA of lipase/cutinase/esterase type. The enzymatic activity of lipase on PLLA was affected by the addition of Na^+^ and K^+^ that increased the activity. However, Zn^+2^, Mg^+2^, Cu^+2^, and Fe^+2^ showed inhibition of the enzymatic activity [350]. Furthermore, the presence of anionic surfactant showed a significant inhibition of Proteinase K activity toward PLLA [302]. However, the presence of the same anionic surfactant showed a dual behavior during the enzymatic activity of α/β hydrolases on PDLLA, facilitating the binding of carboxylesterases on PDLLA and also reducing the hydrolytic activity by lipase-like esterases [301,542]. Nonionic surfactant also reduced the enzymatic activity toward PDLLA by lipase-like esterases [542]. Several studies have reported the importance of factors such as stereochemistry, crystallinity, and hydrophilicity on the enzymatic degradation of PLLA, PDLA, and PDLLA mostly by the action of Proteinase K [543,544,545,546,547,548].

Some biodegradation studies for PLA in the mesophilic range and different environments have reported high values of mineralization, while other studies have reported low values of CO_2_ evolution or mineralization. Ho and Pometto reported values of mineralization for three types of PLA films in soil environments under laboratory conditions; after 180 days of testing, mineralization values ranged from 10 to 45% at 28 °C and from 30 to 90% at 40 °C, depending on the film [246]. The effect of temperature can be also observed in the work of Muniyasamy et al., where mineralization values for PLA films in soil environment at c. 25 °C were a negligible 5% after 190 days of testing [226]. Biodegradation studies by Kim et al. in compost showed different mineralization values for PLA with different *M_w_* and crystallinities after 40 days of testing [248]. The dependence of enzymatic activity on the initial *M_w_* of PLA was evident, with mineralization values of c. 70% for low *M_w_* PLA (5 kDa) and c. 30% for higher *M_w_* PLA (34 kDa). The same study reported a similar trend for different levels of crystallinities at 30 °C, with reduced biodegradation rates for PLA with high crystallinity [248].

In aquatic environments, the biodegradation of PLLA granules resulted in a mineralization value of c. 10% after 180 days of testing at 25 and 37 °C [247]. After 50 days of testing, mineralization values had reached a similar plateau at both temperatures. Lower mineralization values are indicative of limited chemical and enzymatic hydrolysis in aquatic conditions for PLA.

When PLA (initial *M_w_* c. 188 kDa) powder was exposed to UV irradiation and studied by using the Sturm test and in compost for 40 days, the highest mineralization values were found in both the Sturm test (c. 20%) and compost (c. 45%) for samples treated for 8 h; longer UV irradiation treatment times resulted in decreased mineralization values after 40 days of testing [72]. The authors stated that a Norrish reaction was not identified as the main effect for reduced biodegradation with longer UV irradiation time, leaving the presence of crosslinking as the most probable one.

The biodegradation of PLA sheets after 180 days of testing in soil at 28 °C resulted in c. 10% of mineralization [219]. Lower values obtained for PLA sheets in comparison to powder samples indicates the effect of shape and size as important factors decreasing the chemical hydrolysis and the mineralization rate.

The biostimulation and bioaugmentation of soil environments to improve PLA biodegradation under mesophilic conditions was studied by Satti et al. [235]. After 150 days of testing, improved results, with respect to natural biodegradation of PLA, were obtained for biostimulated soil with lactate and bioaugmented soil with previously isolated PLA-degrading bacteria strains. Techniques such as biostimulation and bioaugmentation to improve biotic conditions are increasingly considered as feasible alternatives to increase the biodegradation rate of polymers. In this sense, UV-irradiated PLA sheets in soil, inoculated with *Pseudomonas geniculata* WS3 at 30 °C, showed maximum biodegradation values of c. 35% after 60 days of testing. However, in the case of soil non-inoculated, the biodegradation was just about 15% after 60 days [221].

### 8.5. Poly(Caprolactone)—PCL

PCL is a synthetic, aliphatic biodegradable polymer, semicrystalline in nature, and it is obtained by the ring-opening polymerization of caprolactone [549]. PCL has a *T_g_* of around −60 °C and a *T_m_* of 60 °C. Since the *T_g_* is so low, PCL shows high molecular chain mobility due to its rubbery state [550]. In addition, this low *T*_g_ provides good flexibility and malleability to PCL [549]. PCL is non-hazardous and biocompatible, so the polymer is often used in biomedical applications such as tissue engineering, drug delivery, and in the construction of scaffolds and sutures [551]. PCL displays excellent rheological and viscoelastic properties. Aside from the many listed advantages, the mechanical properties are less suited for rigid applications. The inferior mechanical advantage coupled with improved degradation rate warrants the use of fillers and incorporation of different polymers to attain the necessary mechanical properties. PCL is usually a raw material to produce polyurethanes as polyol polyester-type [504,552].

The main abiotic degradation mechanism for PCL in the mesophilic range is chemical hydrolytic degradation through bulk erosion [553]. Furthermore, PCL can photodegrade when exposed to radiation via Norrish II reactions [68]. UV treatment also has been effective in increasing the degradation rate of PCL films, making it easier for microorganisms to attack during the biodegradation phase [554]. Due to its relatively low *T_m_* (60 °C), PCL can undergo thermal degradation at conditions such as the thermophilic conditions of the industrial composting process. A short abiotic lag phase was reported for PCL in home composting conditions, showing a biodegradation trend similar to readily biodegradable materials such as cellulose or starch [127]. However, the initial *M_w_* of the used PCL was low (*M_w_* c. 50 kDa) in comparison to other polyesters evaluated such as PLA and PHAs [555].

A comparison of the hydrolysis mechanisms for PCL in water and phosphate buffer solutions revealed that in general, enzymatic hydrolysis was faster than abiotic chemical hydrolysis in terms of mass loss, and that enzymatic hydrolysis is a surface erosion process whereas abiotic chemical hydrolysis is a bulk erosion process [347]. However, in more real-world conditions, the enzymatic hydrolysis also could be affecting the chemical hydrolysis. For example, a comparison test for PCL abiotic degradation at 30 °C by *Aspergillus fumigatus* showed a different pattern; samples studied for chemical hydrolysis remained without surface changes, while samples in culture media showed an erosion pattern indicative of surface enzymatic degradation [183].

Table 8 shows that carboxyl esterase (3.1.1.1), lipases (3.1.1.3), and cutinases (3.1.1.74) are able to degrade PCL. In addition, low enzymatic activity by peptidases such as Proteinase K (3.4.21.64) has been observed. Cutinases from fungal phytopathogens are indicated as PCL depolymerases showing enzymatic activity [114]. Based on earlier works studying and identifying aerobic microorganisms able to biodegrade PCL, it has been reported that the natural polymers cutin and suberin are enzymatically degraded by lipases; since these materials are considered as natural analogous to PCL, the enzymatic activity of PCL by lipases was potentially considered. Nishida et al. demonstrated that lipases are highly active in the degradation of PCL [556]. This finding was also indicative of potential microbial populations for PCL biodegradation being extensive in natural environments such as soil, home composting, and water. When the enzymatic degradation of PCL by lipases and Proteinase K available in those environments was studied, lipase activity was reported but none for Proteinase K; the authors associated this result to the preferential specificity of lipases for ester bonds on hydrophobic substrates as in PCL [344].

Temperature and pH are key factors also identified in playing a main role in the degradation of PCL. The high stability of cutinases able to degrade PCL was associated with stabilization of the enzymes by neutral surfaces and additional disulfide bond formation [303]. Baker et al. compared cutinases for PCL degradation and showed that enzyme activity, stability, and efficiency was affected by the type of microorganism that releases the extracellular enzyme and by temperature; the authors reported a similar residual activity for the enzymes at 25 °C but reduced residual activity at 45 °C for some of the cutinases [303].

Li et al. reported the presence of 6-hydroxy-hexanoic acid instead of PCL oligomers during the enzymatic degradation of PCL by *Penicillium oxalicum*, which is indicative of an exo-type chain-end scission by the enzyme [363].

PCL-degrading microorganisms and extracellular enzymes have been reported also in marine environments, showing relatively good activity in comparison to other aliphatic polyesters as PLA [557].

**Figure 19:**
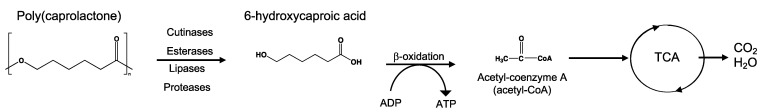
Tentative biodegradation pathway for poly(caprolactone) (PCL) in aerobic conditions. PCL is enzymatically hydrolyzed by extracellular enzymes, namely lipase, esterase and cutinase to 6-hydroxycaproic acid. 6-Hydroxycaproic acid is metabolized via β-oxidation to acetyl-CoA units, which is later metabolized through the tricarboxylic acid (TCA) cycle to produce energy, releasing CO_2_ and water. Adapted from [549].

When 6-hydroxycaproic acid becomes available for bioassimilation after the chemical and enzymatic depolymerization of PCL (Figure 14), it is transported through the semipermeable membrane and then is converted to acetyl-CoA by the β-oxidation of fatty acids, becoming available for the TCA cycle (Figure 19).

A few studies have demonstrated PCL biodegradation with the production of CO_2_ and mineralization at mesophilic conditions such as soil or home composting. Ohtaki et al. reported a low mineralization value of c. 15% for PCL powder (*M_w_* c. 100 kDa) in compost after 8 days of testing [137]. Modelli et al. reported ≈100% mineralization for PCL in a soil environment in laboratory conditions at 22 °C after 270 days of testing [224]. Narancic et al. tested the biodegradation of PCL sheets in home composting and in marine (30 °C) and fresh water (21 °C) environments [127]. In home composting, the PCL reached mineralization values of c. 90% after 180 days of testing relative to the reference material, with a negligible abiotic degradation stage; however, PCL failed the marine (56 days) and freshwater tests, with mineralization values of c. 80 and 50% relative to the reference material, respectively.

### 8.6. Poly(Alkylene Dicarboxylate)s

Poly(alkylene dicarboxylate)s are a family of biodegradable aliphatic polyesters derived from dicarboxylic acids and dihydroxy compounds [44]. This family includes PBA, PBS, PBSA, PBST, PBSe, PBSeT, PEA, and PES, among others. Their general structures are presented in Table 4, and a general description of the main polymers of the family is provided here.

PBA is a biodegradable polyester that can be synthesized via the polycondensation of adipic acid with 1,4-butanediol in the presence of a catalyst. Due to its low *T_m_* (41–61 °C), PBA is generally copolymerized to obtain polyesters with improved mechanical properties. Potential applications for PBA are mainly in the medical area [44].

PBS, a biodegradable, linear, semicrystalline, thermoplastic aliphatic polyester, is the result of the condensation polymerization of succinic acid (SA) and 1,4-butanediol (BDO). PBS can be 100% bio-based (bio-based SA and BDO), partially bio-based (bio-based SA and petrochemical BDO) or fossil-based (petrochemical SA and BDO), depending on the production route used [558]. The SA is derived from maleic anhydride, which can be produced by the oxidation of butane or benzene or from the fermentation of carbohydrate sources such as glucose and starch [559]. The BDO, on the other hand, can be derived via three routes: using petrochemicals, hydrogenation of SA, or fermentation of sugars [560]. PBS provides easy processability and mechanical properties comparable to LDPE and PP. The fact that PBS is flexible and not rigid and brittle like other biodegradable polymers (e.g., PLA, PBAT, and PHB) makes it a more viable and a cost-effective option for common applications [561]. The properties of PBS can be further fine-tuned for designated applications by blending with other polymers. For example, PBS/PLA blends have improved toughness and elongation at break with the help of random copolymers of poly(butylene succinate-*co*-lactic acid) compared with neat blends [562]. PBS applications vary from food packaging, agriculture mulch films, hygiene products, fishing nets, plant pots, and coffee capsules, among others [563].

PBSA is obtained when adipic acid is added when synthesizing PBS. The addition of adipic acid decreases the crystallinity and increases the degradation rate [44]. In comparison to PBS, PBSA has a lower *T_m_* of c. 95 °C (Table 4) and higher flexibility in terms of mechanical properties [498].

PBST is an aliphatic/aromatic polyester synthesized by direct esterification and polycondensation using titanium tetraisopropoxide as a catalyst. PBST has a potential development, however works about PBST biodegradation are limited [44].

PEA, an aliphatic polyester, is produced by the polycondensation of ethylene glycol and adipic acid or by the polycondensation of dimethyl adipate and ethylene glycol [564]. PEA has a *T_g_* of c. −50 °C and *T_m_* of c. 48 °C (Table 4). The polymer displays good flexibility due to the low *T_g_*, but at the same time, it demonstrates low mechanical strength [565]. PEA is usually blended with other polymers. When blended with PLA, PEA helps in reducing the brittleness, improving the thermal stability, and it has also been shown to increase the elongation at break compared with neat PLA [566]. PEA has application as a plasticizer, when low migration, good plasticity and better mechanical properties are desired for the copolymer blend [567].

PES is synthesized by ring-opening polymerization of succinic anhydride with ethylene oxide or by the polycondensation of succinic acid and ethylene glycol (Table 4). PES is highly permeable to O_2_ [44].

In terms of abiotic degradation, the density of the ester bond affects the chemical hydrolysis rate of poly(alkylene dicarboxylates). As reported for PES, a smaller ester bond reduces the hydrophilicity, affecting the overall process of hydrolysis [44]. For PBS, abiotic degradation generally occurs through chemical hydrolysis, with bulk erosion as the predominant mechanism [42]. In general, PBS is copolymerized with the aim of increasing the hydrolysis rate. For example, the addition of more hydrophilic components, such as PEG, has been reported to increase abiotic hydrolysis; however, adipic acid is the most common component added to obtain PBSA [44]. Hayase et al. reported a higher degradation of PBSA than PBS in the presence of *Bacillus pumilus*, which was attributed to the faster degradation of the adipate units [466].

Extracellular enzymes with activity for poly(alkylene dicarboxylate)s have been reported, mostly for PBA, PBS, PBSA, PEA, and PES (Table 8). In general, lipase activity data are scarce for PBA, PBS, and PBSA; however, the activity of cutinases is well reported for PBS and PBSA (Table 8). Cutinases are indicated as being more active for polyesters with chain lengths less than 10 C [498].

Fungi and bacteria have been shown to depolymerize PBS. For example, Ishii et al. [463] reported succinic acid and 1,4-butanediol as hydrolysis products due to the action of *Aspergillus fumigatus* strain NKCM1706. Li et al. [361] reported the action of an exo extracellular enzyme on PBS as an exo attack, since products identified by mass spectrometry were succinic acid and butylene succinate monomers rather than PBS oligomers. Furthermore, the presence of butylene succinate monomers and not 1,4-butanediol showed that the enzyme cut the polymer chain from the carboxyl end [361]. In the case of PBSA, 1,4-butanediol, succinic acid, and adipic acid were detected by HPLC during depolymerization by *Leptothrix* sp. TB-71 [323]. The enzymatic activity of *Rhizopus delemar* lipases against PEA produced, besides ethylene glycol and adipic acid, PEA oligomers, which was indicative of an endo attack of the lipase extracellular enzyme [354].

**Figure 20:**
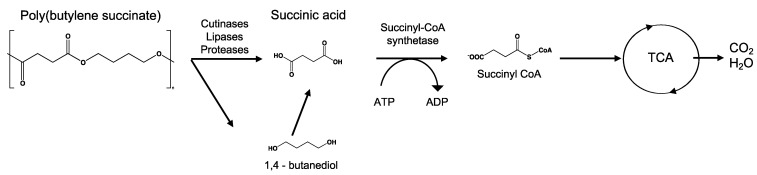
Tentative biodegradation pathway for poly(butylene succinate) (PBS) in aerobic conditions. PBS is enzymatically hydrolyzed by extracellular enzymes, namely lipases, proteases, and cutinases to monomers 1,4-butanediol and succinic acid. Succinic acid is catalytically converted by succinyl-CoA synthetase to succinyl-CoA units, which is later metabolized through the tricarboxylic acid (TCA) cycle to produce energy, releasing CO_2_ and water. Adapted from [568].

When SA and BDO become available for bioassimilation (Figure 14) after chemical and enzymatic hydrolysis, they are transported through the semipermeable membrane and converted to succinyl-CoA, becoming available for the TCA cycle (Figure 20).

Some studies reported mineralization (or CO_2_ production) for PBS, showing limited biodegradation in the mesophilic range, including home composting, soil, marine and freshwater environments. Narancic et al. tested the biodegradation of PBS sheets in soil, home composting, and in marine and freshwater environments, and they found mineralization values lower than 20% after 365 days of testing in soil and home composting relative to the reference material, whereas values were c. 20 and 5% in marine and freshwater after 56 days of testing relative to the reference material, respectively [127]. However, another group evaluated PBS in powder form in soil environments at 25 and 37 °C, and mineralization values reached c. 85 and 80%, respectively, after 180 days of testing [216].

PBSe and PBSeT (films) were assessed in a marine environment at 25 °C under laboratory conditions, and similar mineralization values of c. 90% were obtained after 360 days of testing with stirring and without stirring the media containing the samples [217]. PBSe and PBSeT (films) in soil at 25 °C reached mineralization values of c. 90% after 360 days of testing [215]. Furthermore, when PBSe (powder) was evaluated in soil at 28 °C, mineralization improved for samples with higher available surface area; at day 140, the mineralization values were c. 55% for samples with 33 cm^2^ surface area and 80 to 95% for samples with 89 to 1650 cm^2^ surface area [194].

PBSA films was evaluated in compost at 25 °C with values of mineralization of ≈70% after 55 days of testing, with an abiotic phase duration of ≈1 week [241].

### 8.7. Poly(Hydroxyalkanoates)

PHAs comprise a family of naturally occurring biodegradable aliphatic polyesters produced due to the fermentation of carbohydrate sources, such as sugar and lipids, by the action of a broad range of microorganisms [536,569]. PHAs are synthesized and stored as an intracellular energy resource for later metabolism under conditions of scarcity. PHAs can be classified according to the length of the side chain. The most common are short-chain-length PHAs, with three to five carbon atoms in their monomeric structure [44]. Poly(3-hydroxybutyrate) (PHB), poly(hydroxyvalerate) (PHV), and the copolymer poly(hydroxy-butyrate-*co*-valerate) (PHBV) are the most common, and PHB is abundantly manufactured. PHAs can be derived from renewable and non-renewable sources [570], and they have excellent barrier and good thermo-mechanical properties [571]. However, drawbacks for PHAs in conventional thermal processing include a narrow processing window and high production costs. To improve the processability and ensure large-scale production, PHAs are often blended with other polymers. PHAs have been commonly used for cutlery, trays, food packaging, and cosmetics, and in the development of medical devices, surgical sutures, implants and tissue engineering, among others [572].

The most common PHAs undergo abiotic degradation by chemical hydrolysis scission of the ester bonds (Figure 14). A discussion is still open in the field regarding whether PHAs go through a bulk or surface erosion process regardless of the thickness [42,44]. However, some studies have reported a fast reduction in mass loss, and low reduction of *M_w_* and mechanical properties deterioration, which is indicative of surface erosion as the dominant mechanism [573,574]. Specifically, for the copolymer PHBV, a surface erosion process for both enzymatic and chemical hydrolysis was reported [575].

**Figure 21:**
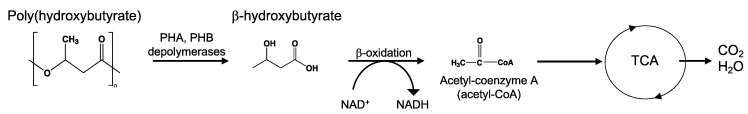
Tentative biodegradation pathway for poly(hydroxybutyrate) (PHB) in aerobic conditions. PHB is enzymatically hydrolyzed by extracellular enzymes, namely PHA, PHB depolymerases to β-hydroxybutyrate. β-hydroxybutyrate is metabolized via β-oxidation to acetyl-CoA units, which is later metabolized through the tricarboxylic acid (TCA) cycle to produce energy and releasing CO_2_ and water.

After the depolymerization of PHB (as an example of PHAs), 3-hydroxybutyric acid is bioassimilated and, through a redox reaction, converted to acetyl-CoA, which feeds the TCA cycle (Figure 21).

The degradation of short-chain-length PHAs by enzymatic activity from bacteria and fungi has been extensively reported (Table 8) [42,44,498,576]. In general, an increase in side-chain length decreases the hydrolytic rate of the PHAs [368].

PHAs can be metabolized by intra or extracellular depolymerases depending on its location. In this sense, in vivo granules are amorphous PHAs that can be metabolized by intracellular enzymes. Denatured PHAs, after cell lysis, become semicrystalline and can be depolymerized by microorganisms that release extracellular depolymerases [498,577].

Table 8 lists PHB depolymerases (3.1.1.75) and PHA depolymerases (3.1.1.76) as the main enzymes reported as able to degrade PHB and other PHAs. A bacterial PHB depolymerase has been shown to have two functions during the hydrolysis of PHB films, which takes place via adsorption and hydrolysis, binding, and catalytic domains [375]. Investigations revealed that the binding domain of the enzyme is non-specific for binding to the surface of PHA films; however, the active site in a catalytic domain is specific for the hydrolysis of the PHB molecule [375].

The activity of extracellular PHB depolymerases in enzymatic depolymerization occurs initially on the surfaces of the polymer after biofilm formation, and the rate is dependent on the *M_w_*, crystallinity, and microbial community [578]. PHA depolymerases can be described as serine hydrolases with protein progression formed by four regions. First is the signal sequence, second is a catalytic area which contains the lipase box, third is a substrate-binding domain where the adsorption of the polymer substrate takes place, and eventually, there is a domain that connects the catalytic area to the substrate securing areas [114].

Stimulation activity for PHB depolymerase was observed in the presence of Na^+^, K^+^, Ca^2+^, and Mg^2+^ [116,389]. However, Fe^+2^, Hg^+2^, Mn^+2^, and Cu^+2^ were reported as inhibitory of enzymatic PHB activity [408,498].

Nishida et al. reported the effect of crystallinity and amorphous fraction on microbial degradation [579], showing that increased crystallinity repressed microbial activity.

PHB, PHV, and PHBV have been reported to be biodegradable in several mesophilic environments such as soil, compost, and water (Table 7). A large microbial population has been identified as associated with biodegradation of PHB and the copolymer PHBV in mesophilic conditions [44]. Biodegradation in the soil of PHBV films was reported as the combined action of fungi, bacteria and actinomycetes; however, the fungi population was identified as the dominant one due to the ability to increase the surface growth of hyphae [42,457]. Copolymer composition, crystallinity, microstructure, and surface morphology are factors reported to play an important role during the biodegradation of PHBV in soil [222]. During the degradation of PHBV in seawater, an increase in surface roughness was observed, which was reported as both surface erosion by chemical hydrolysis and enzymatic activity [42,580].

During the biodegradation of PHBV (films) evaluated in soil at 25 °C, mineralization values of c. 25% were achieved after 190 days of testing [226]. Higher mineralization values were reported for PHBV in marine environments. Thellen et al. [238] reported high mineralization for high *M_w_* PHBV films with different contents of valerate in a marine environment at 30 °C; after 100 days of testing, mineralization values reached c. 100% for PHBV with 5 and 8% of valerate, and c. 90% for PHBV with 12% valerate. A recent study by Meereboer et al. [240] evaluated PHBV in powder form in a simulated marine environment at 25 °C; mineralization values were higher than 50% at day 190, and values reached c. 90% at the end of the test (450 days).

The mineralization of PHB in a soil environment was reported after 360 days of testing, with a degree of biodegradation of c. 95% at 25 °C [215]. High mineralization values were also reported by Narancic et al. [127]. After 136 days of testing in soil at 25 °C, mineralization values higher than 100%, showing priming effect, were reported; however, when evaluated in home composting, low mineralization values (less than 20%) were reported for PHB at 28 °C [127].

On the other hand, PHB in a marine environment showed c. 70% of mineralization after 360 days of testing and c. 95% in the same test after 200 days, but with a stirring system; the difference in CO_2_ evolution was attributed to the shortage of O_2_ in the system without stirring [217]. Thellen et al. also reported high mineralization values (in the range of 80 to 90%) for high *M_w_* and high crystallinity content PHB films in a simulated marine environment at 30 °C; this work was indicative of the high degradability of polymers from the PHA family, even though the percentage crystallization and initial *M_w_* was high for both PHB and PHBV [238]. Narancic et al. reported mineralization values of c. 90% for PHB sheets at day 56 of testing in a marine environment at 30 °C relative to the reference material, whereas in a freshwater environment at 21 °C, the values were c. 90% relative to the reference material for PHB [127].

PHA biodegradation has been evaluated in soil. Gómez et al. reported mineralization values of c. 70% for PHA (injection molding samples) in soil after 650 days at 20 °C [234]. A more recent study reported mineralization values of c. 90% for PHA (powder) in soil after 150 days at both 25 and 37 °C [216].

### 8.8. Poly(Butylene Adipate-co-Terephthalate)

PBAT is a co-polyester synthesized from 1,4-butanediol (BDO), adipic acid (AA), and dimethyl terephthalate by a polycondensation reaction. Adipic acid and BDO polymerize to produce their own polyester and water. Dimethyl terephthalate and BDO react to form their own polyester and methanol. The resulting polyester then reacts with the polyester of AA and BDO using tetrabutoxytitanium as a catalyst for the transesterification. The reactions are carried out at temperatures higher than 190 °C, under high vacuum, and usually require long times [581]. PBAT has a *T*_g_ of c. 30 °C and *T*_m_ of c. 106 °C. PBAT has good toughness and ductility, biodegradability, and is flexible. However, higher production costs, coupled with lower mechanical and heat resistance in comparison to common fossil-based plastics, has hindered PBAT development and acceptance in the consumer market [581]. These shortcomings can be overcome by blending PBAT with other biodegradable polymers. For example, blends of PBAT and PLA demonstrated higher yield stress, modulus, and rheological properties than those of neat PBAT [582]. PBAT is widely used for agricultural mulch films and also for packaging applications including trash bags, shopping bags, wrapping films, and disposable food containers [583].

The reported abiotic mechanisms of degradation associated with PBAT are primarily mechanical, photodegradation, thermal, and hydrolysis. Mechanical degradation is, in general, associated with the entire spectra of biodegradable polymers; in the case of PBAT, erosion is a common situation due to its main application in agricultural mulch films. Photodegradation has been reported as the main abiotic mechanism of degradation for PBAT agricultural mulch films. A crosslinking effect as a result of exposure to sunlight has been reported; the effect is known to delay the biodegradation rate of PBAT by decreasing the chain mobility of the polymer [70,180,284].

The addition of aliphatic acids to aromatic polyesters improves the water uptake and hydrolysis of these polymers. However, PBAT still offers more resistance to chemical hydrolysis than aliphatic polyesters, such as PLA, due to the steric hindrance of the large aromatic ring repeating units.

**Figure 22:**
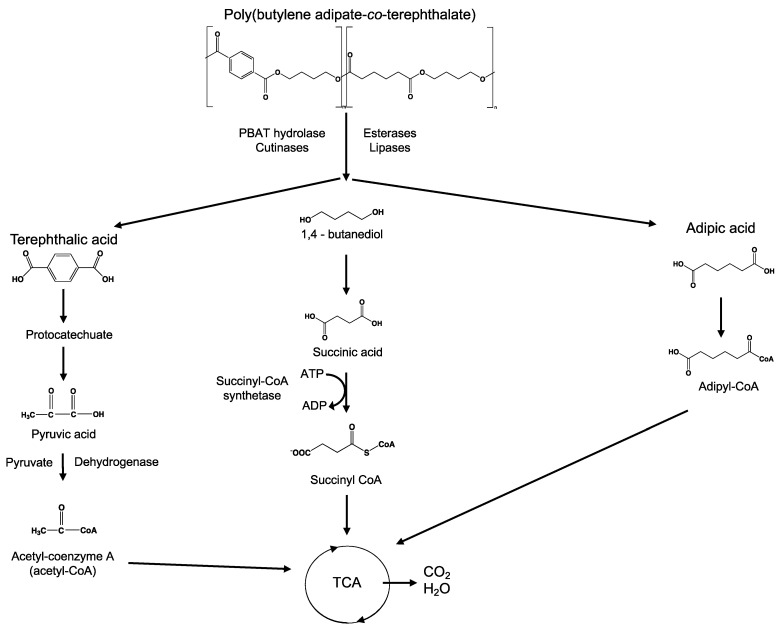
Tentative biodegradation pathway for poly(butylene adipate-*co*-terephthalate) (PBAT) in aerobic conditions. PBAT is enzymatically hydrolyzed by extracellular enzymes—namely, PBAT hydrolase, cutinases, esterases, and lipases—to its monomer constituents of terephthalic acid, 1,4-butanediol, and adipic acid. Terephthalic acid is broken down into protocatechuate, which is further metabolized to pyruvic acid, and acetyl-CoA units. 1,4-butanediol is oxidized to 4-hydroxybutyrate and subsequently to succinyl-CoA units. Adipic enters the tricarboxylic acid (TCA) cycle by conversion to adipyl-CoA, which is later metabolized through the tricarboxylic acid (TCA) cycle to produce energy, thereby releasing CO_2_ and water. Adapted from [568,584].

PBAT is depolymerized through chemical and enzymatic hydrolysis into adipic acid, 1,4-butanediol, and terephthalic acid (Figure 14). Then, each compound is bioassimilated or undergoes a redox reaction to feed the TCA cycle, as shown in Figure 22. The adipic acid pathway is through adipyl-CoA, and 1,4-butanediol is converted to succinic acid and to succinyl-CoA. Several bioassimilation pathways have been reported for terephthalic acid. The most probable, in the case of PBAT, seems to be the transport of terephthalic acid through the cell membrane, which is followed by degradation to protocatechuic acid and then through the pyruvic acid pathway to Acetyl-CoA to enter the TCA cycle [568].

In terms of the biodegradation and enzymatic activity at mesophilic conditions, PBAT has been reported to be degraded by cutinases [307], lipases [340], and PBAT hydrolases [268] (Table 8). As for chemical hydrolysis, enzymatic activity is affected due to the presence of aromatic groups that make enzyme accessibility more difficult for scission of the ester bonds that are close to these groups [44,45]. The presence of the aromatic ring has been associated with a decrease in the enzymatic activity by creating a steric impediment to access the active site of the enzymes. Butanediol-terephthalate bonds have been reported to be hydrolyzed at a lower rate in comparison to adipate-butanediol bonds [95].

The low values of enzymatic activity reported for the actinobacteria *Rhodococcus fascians* in comparison to a mesophilic PBAT-degrading fungus show that both the type of enzyme and the microorganism producing the enzyme play major main roles in activity [267,268]. These results could be associated with the favorable conditions offered to microorganism populations in soil environments in the mesophilic range. The enzymatic hydrolysis of PBAT by a fungal strain generated terephthalic acid, adipic acid, and 1,4-butanediol, as identified by mass spectroscopy [268]. Furthermore, the enzymatic activity of PBAT hydrolase by *Bacillus pumilus* on PBAT showed degradation products such as adipate, 1,4-butanediol, and terephthalate [95].

Crosslinking due to exposure to UV-radiation treatment has also been shown to decrease the enzymatic activity against PBAT due to the reduced flexibility of the polymer chains after crosslinking [585].

PBAT has been reported to be degraded in soil environments or soil in laboratory conditions (Table 7). In general, rates of biodegradation at mesophilic conditions are low. Biodegradation studies of PBAT showing CO_2_ and mineralization in simulated and controlled media in the mesophilic range are limited [148]. Studies in more controlled environments such as culture and/or buffer media are more commonly focused on the identification of microbial activity and/or enzymatic activity toward PBAT. However, the identification of extracellular enzymes able to degrade PBAT is relatively limited in comparison to those for common aliphatic polyesters. Most of the environments assessed for PBAT degradation are agricultural soils. CO_2_ production from PBAT in soil environment media has been reported, with mineralization values of c. 10% after six weeks [148].

A novel approach by Zumstein et al. [148] demonstrated the mineralization of PBAT ^13^C to ^13^CO_2_, with higher values of mineralization for ^13^C derived from depolymerization of the adipate structure, and lower values of mineralization associated with depolymerization of the aromatic terephthalate fraction. This finding is indicative of the increased complexity of aromatic polyesters toward depolymerization and assimilation. On the other hand, the presence of the aromatic component in the co-polyester was shown to improve the overall rate of biodegradation, even in the mesophilic range like in the soil environment evaluated [148].

PBAT films with 1% of a chain extender had low mineralization values of c. 20% after 180 days in soil at 28 °C [219]. The effect of the chain extender on delaying *M_w_* reduction and biodegradation was evident.

An interesting outcome of biodegradation studies for PBAT in a soil environment is that the degradation products have been shown to be harmless to the microbial population [586]. Although biodegradation can be a longer process in the mesophilic environment, and the formation of PBAT degradation products does not affect the quality and health of the soil and its microbial population, the development of some microorganisms over others can be modified [587].

### 8.9. Poly(Urethane)—PU from Esters

**Figure 23:**
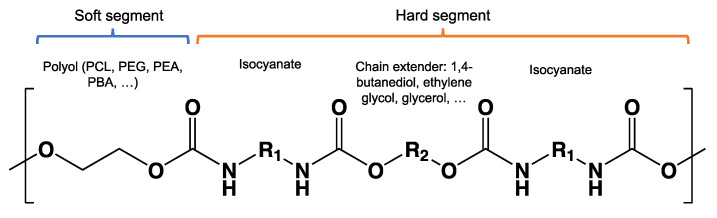
Soft segment (polyols) and hard segment (isocyanates) of the poly(urethane) structure. Adapted from [504].

PUs are synthetic plastics, insoluble in water, and produced by the condensation reaction of polyols and polyisocyanate having urethane bonds [43,588]. Polyisocyanates and polyols react with a chain extender to give polyurethane polymers with alternate soft and rigid segments. Polyol forms the soft segment and can be obtained from polyester or polyether polyols; whereas the rigid segment is derived from the isocyanate and chain extender, and it has restricted mobility compared with the soft polyol segment (Figure 23) [504]. The rigid segment is considered the crystalline region and the soft segment is considered the non-crystalline or amorphous region of PUs [502,589]. Depending on the polyol used, the resulting PU can be identified as polyester PU or polyether PU. The resulting properties and degradation behavior are dependent upon the selection and chemistry of the soft segment [590].

Poly(urethane)s are used in the medical, construction, and automotive fields, among others. Products that contain PUs include furniture, paints, fibers, flexible foams, rigid foams, coatings, adhesives, synthetic skins, sutures, and tissue scaffolds [503,591,592]. Poly(urethane) elastomers (thermoplastic) are used in the medical field due to their high elasticity and toughness compared with other elastomers [588,591]. The good mechanical, thermal, and electrical properties of PUs allow these polymers to offer good adhesion for coatings, tensile strength, and abrasion resistance for several uses [591]. Poly(urethane) foam are a typically example of thermoset PUs [503].

**Figure 24:**
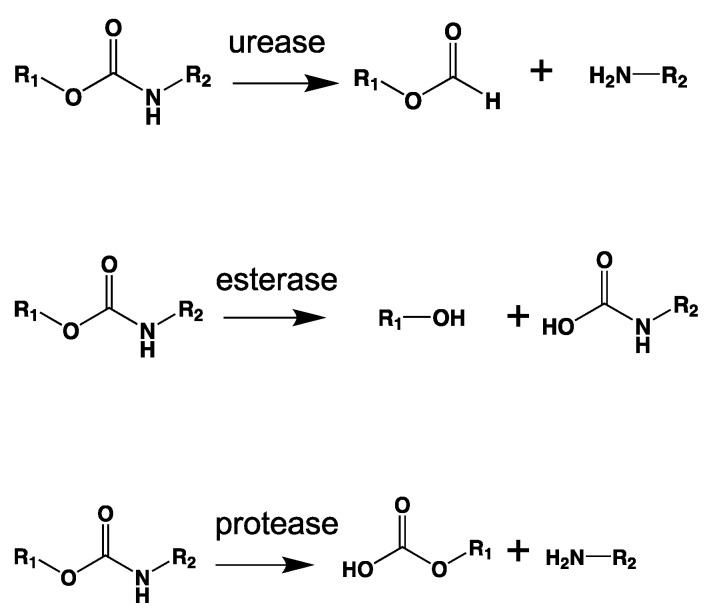
Sites of scission for urethane bonds by extracellular enzymes. Different products are produced depending on the scission site and extracellular enzymes involved. Three different sites of breakdown are possible because of the action of ureases, esterases, and proteases enzymes. Adapted from [589].

Early studies demonstrated that PUs with long repeating units and hydrolytic groups were susceptible to some extent of biodegradation [593]. This review concentrates on polyester PUs. The ester bond of polyester PUs is susceptible to hydrolytic degradation and can be catalyzed with the help of extracellular enzymes (Table 8). The extracellular enzymes for PU degradation have a hydrophobic area, which assists in attaching onto the polymer surface [589,591]. The microbial attack of PUs can occur by the action of extracellular hydrolases such as ureases (3.5.1.5), amidases (3.5.1.4), proteases, and esterases (Figure 13). The cleavage site and the product of the breakdown is dependent on the type of the enzyme acting during depolymerization (Figure 24). Adipic acid and diethylene glycol were reported as degradation products by the action of extracellular enzymes on polyester PUs; however, no identification of the isocyanate hard segment by-products was reported [324,325]. Later work by Shah et al. reported the probable presence of a hydrolyzed portion of the hard segment, detected by FTIR spectrum, when polyester PU was attacked by both *Bacillus subtilis* MZA-75 and *Pseudomonas aeruginosa* MZA-85 [254]. Furthermore, the mixing of esterase and amidase has been reported to hydrolyze the hard segment via the urethane bonds [445].

A bacterial esterase was identified to degrade ester PUs by acting in a two-step reaction: first, a hydrophobic adsorption of the enzyme on the surface of the PU; and second, the hydrolysis of the ester bonds of the PU [325]. Studies of enzymatic activity have shown that the rate of biodegradation decreases with decreasing ester content, indicating the impact of the esterase activity as relevant for PU depolymerization [588]. Fungal communities have been identified to degrade PU to some extent (Table 8) [490].

**Figure 25:**
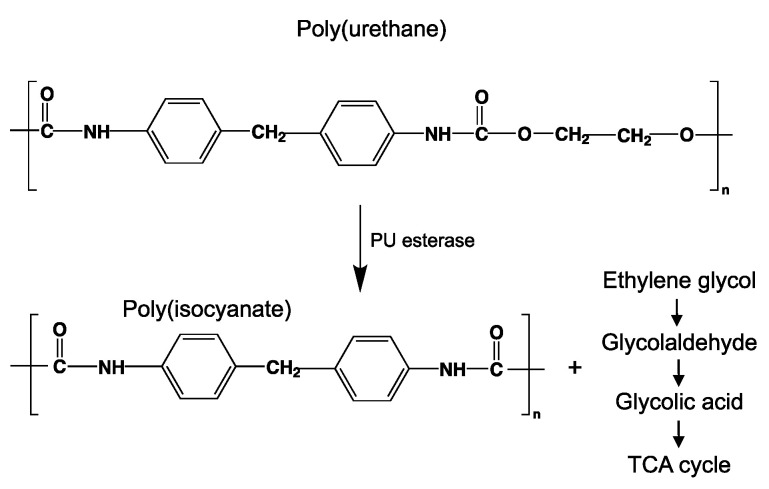
Tentative metabolism pathway for poly(urethane) (PU) derived from esters. PU is enzymatically hydrolyzed by extracellular enzyme, PU esterase to polyisocyanate and ethylene glycol. Ethylene glycol is oxidized to glycolaldehyde and subsequently to glycolic acid. Glycolic acid is converted to pyruvic acid via glycolysis. Pyruvate dehydrogenase catalyzes the conversion of pyruvic acid to acetyl-CoA, which is later metabolized through the tricarboxylic acid (TCA) cycle to produce energy, thereby releasing CO_2_ and water. Adapted from [594].

A tentative route for metabolism of the soft segment (PEG) is presented in Figure 25 for PU derived from ester.

Biodegradation studies showing CO_2_ evolution or mineralization in mesophilic environments for PUs are limited and show scarce evolution. Most of the reported studies on PUs are for the enzymatic activity of both fungi and bacteria (Table 8). More investigations of the abiotic degradation process of PUs prior to the biotic degradation stage, such as hydrolysis or photodegradation, in the mesophilic range would help determine whether PUs derived from esters are biodegradable in soil, home composting, industrial composting, or water environments. The biodegradation of polyester-PUs studied under mesophilic composting conditions resulted in mineralization between 5 and 43% after 45 days of testing, and this wide range was attributed to the different chemical structures of the PUs [595]. A high content of the hard segment led to decreased biodegradation rates and mineralization, whereas biodegradation increased as the amount of diol carbon chains of the polyol (soft segment) increased. The hard segment composition in PUs was presented as a more dominant effect than the crystallinity or surface properties during PU biodegradation in composting [595]. The presence of aromatic diisocyanates decreased the rate of biodegradation in comparison to PUs with aliphatic diisocyanates [595]. 

The biodegradation of PU films during the Sturm test showed high CO_2_ evolution at 30 °C for 28 days in comparison to the control [249]. In addition, a Sturm test revealed the production of CO_2_ during the enzymatic hydrolysis of PU films by *Bacillus subtilis* MZA-75 and *Pseudomonas aeruginosa* MZA-85, hydrolyzing the ester portion in 1,4-butanediol and adipic acid products [254,255,256]. This result indicated that *Bacillus subtilis* was able to hydrolyze and assimilate the intermediates as carbon sources with final mineralization. An interesting outcome of the reported enzymes attacking ester PUs is the evidence of the presence of membrane-bound enzymes, besides extracellular enzymes. For an esterase not secreted to the culture medium, its high hydrophobicity was reported as the most probably cause for its membrane-bound characteristic [552].

A new approach is the development of non-isocyanate PUs (NIPU). NIPU are a promising and more sustainable alternative for traditional PUs [596,597]. However, studies in this area looking at degradation and biodegradation are still limited. Production and biodegradation assessment of polyhydroxyurethane, a NIUP based on cyclic carbonate and polyamine, was reported by Ghasemlou et al. [230]. Mineralization values for film samples reached c. 40% after 120 days of testing in soil conditions.

### 8.10. Poly(Vinyl Alcohol)—PVOH

PVOH is a synthetic, water-soluble polymer produced by the partial or complete hydrolysis of polyvinyl acetate. Unlike other polymers, PVOH is not synthesized from the polymerization of its monomer (vinyl alcohol), which is due to the unstable nature of the high density of hydroxyl groups in the monomer. Polyvinyl acetate is first synthesized by the polymerization of vinyl acetate and then subjected to saponification, wherein the ester groups of vinyl acetate are replaced by hydroxyl groups in the presence of caustic soda [598]. Different grades and properties of PVOH are available, depending on the degree of hydrolysis and the variation in initial length of the vinyl acetate polymer. PVOH is odorless and non-toxic in nature, has excellent resistance to aroma and gases, is resistant to solvents and oil, and it has good optical and adhesive properties as well as film-forming capacity [599]. In terms of disadvantages, PVOH is expensive, and mechanical properties are highly conditioned by the presence of water or humidity, so it needs to be blended with other polymers to achieve more desirable properties [600]. Due to its good adhesion to other hydrophilic surfaces, PVOH is used widely in emulsifiers, binders, and hydrogels for a broad range of industries, including textile, paper sizing, fabrics, and packaging films as a protective film for laundry and dish detergents [601,602]. The applications are not limited and extend to the biomedical, cosmetic, and food packaging industries [603,604].

The degree of solubility of PVOH in water can be tailored, depending on the amount of OH groups and remaining acetate bonds.

In addition to the abiotic mechanism of biodegradation, PVOH could be considered as partially biodegradable, since the number of microorganisms and enzymes identified to biodegrade it is rather scarce in comparison to polyesters.

The biodegradation of PVOH has been reported to start from random chain scission where the action of oxidative enzymes catalyzes the break of the carbon backbone. Mostly dehydrogenases or oxidases are responsible for the carbon-carbon bond scission. Hydrolases or aldolases have been reported as responsible for the chain scission of the hydroxyl group (Figure 14). Furthermore, a two-step process has been proposed for the enzymatic degradation of PVOH: the first step, by the action of PVOH oxidases, involves the oxidation of hydroxyl groups to form diketone or monoketone structures; and the second step involves hydrolysis of the carbonyl structure formed by oxidized PVOH hydrolases [605].

Since PVOH is a water-soluble polymer, its biodegradation has been studied mostly in aqueous media. The identified microorganisms and enzymes able to biodegrade PVOH are associated mainly with contaminated environments, such as waste sludge, which are common end-of-life scenarios for PVOH (Table 8).

Abiotic degradation of PVOH by UV/chlorine oxidation via the generation of active free radicals has been investigated; in acidic media, the efficiency was higher due to the higher ratio of [HOCl]/[OCl^-^] [606]. The abiotic degradation of PVOH by photocatalytic oxidation or radiation and ozone also has been reported [74,487].

**Figure 26:**
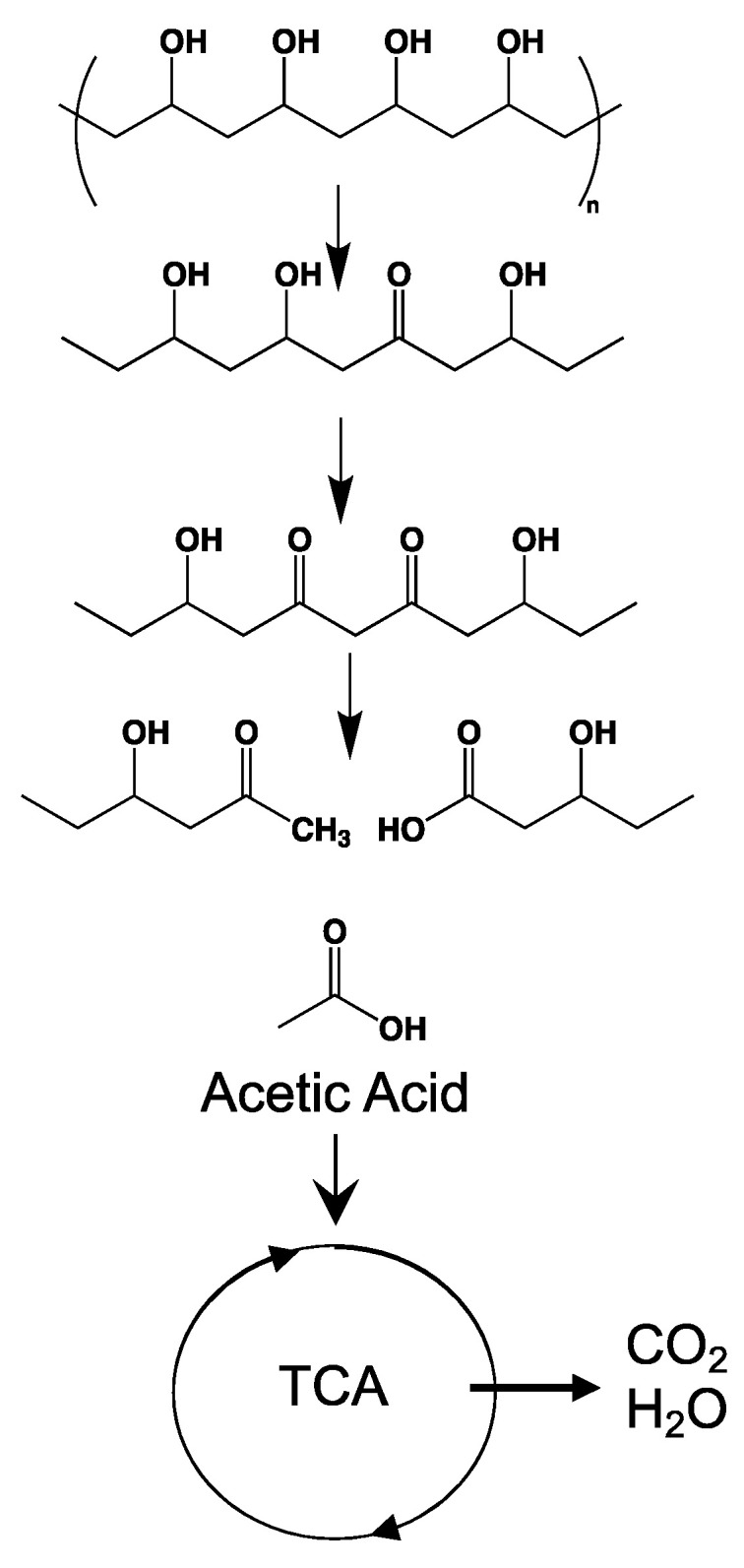
Proposed pathway for metabolism of poly(vinyl alcohol) (PVOH). PVOH is cleaved by extracellular enzymes, dehydrogenase, and oxidase. Subsequently, hydrolase and aldolase further break down to acetic acid, which is further metabolized through the tricarboxylic acid (TCA) cycle to produce energy, thereby releasing CO_2_ and water. Adapted from [121,608].

Published works have identified that microorganisms able to biodegrade PVOH are mostly from the genus *Pseudomonas* [607]. In addition, many PVOH degradation pathways have been proposed for different bacteria such as *Alcaligenes* and *Pseudomonas* species [608]. These routes include scission of the polymer chain by an extracellular oxidase (dehydrogenase), followed by aldolase and hydrolase reactions, releasing compounds such as acetic acid and hydroxyl fatty acids that can be incorporated into the β-oxidation and TCA cycle, respectively [114]. Figure 26 presents a tentative metabolization route for PVOH [447].

As stated, scarce mineralization was reported for PVOH films in water conditions, with ≈10% after 100 days of testing at c. 30 °C [239].

## 9. Final Remarks and Future Perspective

The impacts of plastic waste and pollution in terms of global climate change, health, and social effects, circular economy, sustainable use of resources and production, and improved waste management systems have garnered the attention of industry, government, NGO stakeholders, and society in general. The development of new plastic waste pacts and commitments to curb the use of virgin plastic are ongoing globally, with targets for 2025 [18]. However, the damage to ecosystems has already created detrimental impacts, which will require forward-thinking actions to remediate, to mitigate, and to avoid permanent damage [6].

The development of biodegradable polymers derived from both bio- and fossil-based resources has transcended from the lab scale to commercial applications in the last two decades, and these polymers have become an option for packaging and consumer goods applications to mitigate the impact of plastic waste. However, biodegradable polymers must reach a waste management end-of-life to avoid a rebound effect on creating additional pollution.

The degradation process for biodegradable polymers starts by the action of external abiotic and biotic factors. The main abiotic mechanisms of degradation associated with mesophilic environments are mechanical degradation, photodegradation, and chemical hydrolysis. The initial deterioration of the polymer structure enhances the mechanical degradation, generating micro and nano plastics but not guaranteeing biodegradation. Photodegradation in the presence of O_2_ introduces modifications during the degradation of biodegradable polymers in specific environments such as agriculture soils, inducing a dual effect: chain scission that contributes to the degradation and crosslinking that acts to delay the process. Chemical and enzymatic hydrolysis are the crucial mechanism for most biodegradable polymers, since most of them contain ester bonds that are prone to water attack.

The formation of biofilms affects the whole dynamic of the degradation process. Since biofilms create an extra layer on the polymer surface and potentially affect water diffusion during chemical hydrolysis, a better understanding of biofilm interactions on polymer surfaces and its effect on water diffusion and bulk erosion are needed.

Extracellular enzymes act at the surface level of polymers, making enzymatic activity a surface erosion process. As presented in this review, the main groups of enzymes reportedly able to break chemical bonds in polymers belong to the esterase group (amidases, cutinases, esterases, lipases, and PHA depolymerases), proteases (specific for PLLA), and oxidoreductases (for PVOH and PUs derived from esters). Recent advances in the identification of protein sequences and residues, structural domains, mechanisms of substrate binding, kinetic analysis, and the presence and effects of cofactors have provided a better understanding of enzymatic activity on biodegradable polymers. However, a better understanding of bioassimilation and mineralization is still needed at the biochemical level of monomer compounds produced from the chemical and enzymatic hydrolysis of biodegradable polymers. 

In terms of polymer properties, the key bulk properties affecting biodegradation in the mesophilic range are stereochemistry, crystallinity, and *M_w_*, which are tailored for each application. The amorphous region offers the optimal conditions for chemical hydrolysis due to the easy diffusion of water and for exo and endo enzymatic attack by extracellular enzymes. Microorganisms start the assimilation process when low *M_w_* compounds such as dimers and monomers are released. Since biofilm formation, microbial colonization, and enzymatic activity are surface-related processes, the key surface properties of polymers impacting biofilm formation and colonization are hydrophobic/hydrophilic balance, roughness, and surface energy.

This review has summarized enzymes and microorganisms isolated from several environments that show activity toward aliphatic, aliphatic–aromatic polyesters, PUs derived from esters, and PVOH. Usually, the identification of microorganisms and/or enzymes involves techniques, such as culturing, where the polymer is the sole source of carbon for the biotic process. These studies provide unique insights on enzymatic activity and pathways of degradation. However, natural environments introduce far more complexities to the degradation process, creating a dynamic that undoubtedly affects the rate of degradation. Microbial consortia have demonstrated an increased efficiency for the elimination of toxic metabolites in comparison to pure cultures. Studies showed that some microorganisms are directly involved in the degradation process, while other microorganisms showed activity toward eliminating toxic metabolites excreted by the first ones. However, besides symbiotic, mutual, and synergistic interactions, efficiency differs among microbial consortia. The complex tracking of microorganism population dynamics during biodegradation should provide better insights on the real pathways of degradation and assimilation of these polymers in actual environments. Research in the areas of biostimulation, bioaugmentation, and engineering of enzymes are needed to address the complexities associated with microbial consortia involved in the biodegradation process, extracellular enzymes, and biocatalytic cascades of enzymes.

Standards, methodologies, and techniques have been developed to assess the degradation of polymers in the environments. Some are more focused on evaluating the degradation of mechanical properties and the mass loss due to various factors of abiotic mechanisms. However, the assessment of CO_2_ or O_2_ and ultimate mineralization must be the definitive assessment to determine the extent of biodegradability in a specific environment. The use of complementary techniques, such as carbon tracking and *M_w_* reduction, constitutes important tracking parameters that must be incorporated when evaluating biodegradation to the mineralization level.

Many works reported the CO_2_ and/or mineralization for the biodegradable polymers available in the market in soil, home composting, and aquatic environments at mesophilic conditions using several standards. From the aliphatic polyesters group, chemical hydrolysis has been reported to be the main controlling step. Aliphatic polyesters, such as PCL, PBS, and PBSA, were consistently reported to biodegrade in soil conditions. However, for the aliphatic–aromatic polyester PBAT, biodegradation in soil and marine environments is limited. The degradation of the natural polyesters PHB and PHBV in aquatic environments has been extensively reported, showing the high level of biodegradability at mild conditions. For the PUs, the presence of the soft segment offers availability for enzymatic attack and biodegradation with mineralization at some low extent; however, the bioassimilation pathway of the hard segment has not been identified and/or described. At this point, results show that the hard segment is unlikely to be mineralized at mild environmental conditions. 

Currently, innovative approaches to tailor the biodegradation of biodegradable polymers are opening new routes to accelerate the biodegradation process, especially for polyesters [609]. The addition of specific compounds to trigger depolymerization in particular conditions, biostimulation, bioaugmentation, and the addition of natural [610,611,612] and/or modified enzymes [613] are state-of-the-art methods. These new methods must be connected to standards and techniques that allow for full tracking of the biodegradation process and the end products even under mild conditions so that further insights on the biodegradation and metabolic pathways of polymers can be successfully clarified. In terms of future perspectives, a redesigned polymer constitutional unit, based on novel circular principles, to tailor degradation is highly needed if the goal is to eliminate the persistence of plastics across environments. This will require not only polymer chemistry/processing professionals but more meaningful transdisciplinary work, including microbiologists and biochemists.

## Figures and Tables

**Table 1:** Half-lives (time required for 50% hydrolysis) of hydrolysable bonds for different polymers (in water at pH 7 and 25 °C). Adapted from [85].

Polymer	Chemical Structure	Half-Life *
Poly(anhydrides)	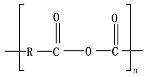	0.1 h
Poly(ketal)	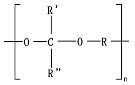	3 h
Poly(ortho esters)	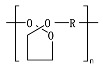	4 h
Poly(acetal)	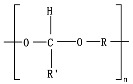	0.8 years
Poly(ester)	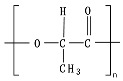	3.3 years
Poly(urea)	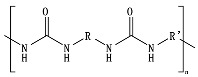	33 years
Polycarbonate	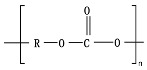	42,000 years
Polyurethane	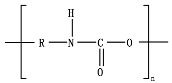	42,000 years
Polyamides	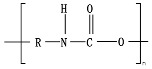	83,000 years

* Half-life: time required for 50% hydrolysis in water at pH 7 and 25 °C for the low *M_w_* (methyl, ethyl) model compounds.

**Table 2:** International Union of Biochemistry and Molecular Biology (IUBMB) classification of enzymes by the type of reactions they catalyze. Adapted from [112].

EC * Number	Enzyme Class	Reaction
1	Oxidoreductases	Oxidation-reduction
2	Transferases	Chemical group transfers
3	Hydrolases	Hydrolytic bond cleavages
4	Lyases	Nonhydrolytic bond cleavages
5	Isomerases	Changes in arrangements of atoms in molecules
6	Ligases	Joining together of two or more molecules

* Enzyme codes according to the Enzyme Commission (EC) nomenclature [112].

**Table 3:** Typical temperature ranges and conditions for different environments where polymers can be subjected to aerobic biodegradation.

Temperature Range, °C	Environment	General Description	Management
20–30	Soil/Agricultural soils	Large scale. Soil structure (texture, porosity), moisture, aeration, radiation	Uncontrolled
20–45	Home composting	Small scale. C/N ratio, moisture, aeration, heat, pH	Controlled
45–60	Industrial composting	Medium scale. C/N ratio, moisture, aeration, pH	Controlled
0–30	Aquatic	Large scale	Uncontrolled

**Table 4:** Polymer structure, glass transition temperature (*T_g_*) and melting temperature (*T_m_*) of the biodegradable polymers discussed in this review.

Polymer	Structure	T_g_, °C	T_m_, °C
Poly(3-hydroxybutyrate-co-3-hydroxyvalerate) (PHBV)	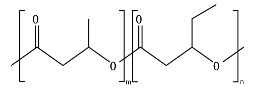	−8 to −1	180
Poly(butylene adipate-co-terephthalate) (PBAT)	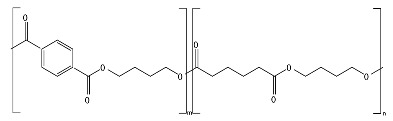	−30	106
Poly(butylene adipate) (PBA)	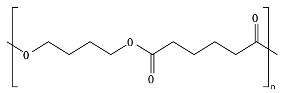	−61 to −64	41–61
Poly(butylene sebacate terephthalate) (PBSeT)	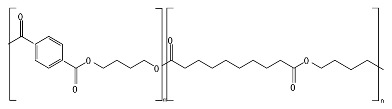	≈ −43	25 to 91
Poly(butylene sebacate) (PBSe)	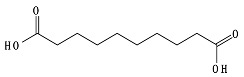	−62	65
Poly(butylene succinate terephthalate) (PBST)	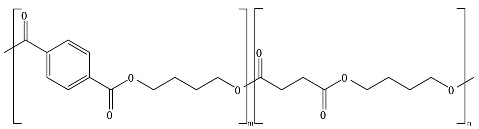	−20 to −30	≈179
Poly(butylene succinate-co-adipate) (PBSA)	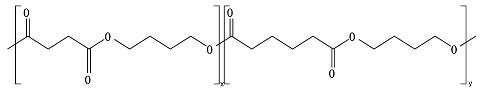	−43 to −45	95
Poly(butylene succinate) (PBS)	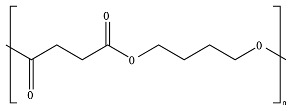	−28 to −32	112–114
Poly(caprolactone) (PCL)	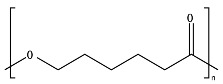	−60	58–63
Poly(ethylene adipate) (PEA)	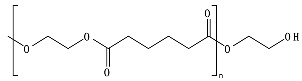	−46 to −50	48
Poly(ethylene succinate) (PES)	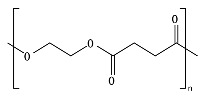	−9 to −17	96–105
Poly(glycolic acid) (PGA)	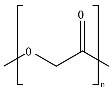	35–40	220–230
Poly(hydroxybutyrate) (PHB)	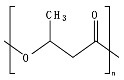	4	180
Poly(hydroxyvalerate) (PHV)	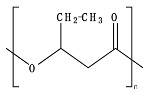	−10	100–200
Poly(lactic acid) (PLA)	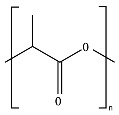	55–65	170–200
Poly(urethane) (PU)	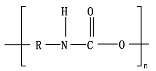	−63	-
Poly(vinyl alcohol) (PVOH)	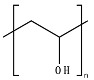	-	-

**Table 5:** Water diffusion and surface property as related to the main degradation process and associated polymer examples.

Water Diffusion	Surface	Degradation Process	Example
Low	Hydrophilic	Surface	PHA
High	Hydrophilic	Bulk/surface	Starch, TPS, Cellulose
High	Hydrophobic	Bulk	PLA, PCL, PBS
Low	Hydrophobic	Surface (depending on the ratio of hydrophobic depletion and water diffusion)	PLA with chain extender

PHA, poly(hydroxyalkanoates); TPS, thermoplastic starch; PLA, poly(lactic acid); PCL, poly(caprolactone); PBS, poly(butylene succinate).

**Table 6:** Standards for assessing aerobic biodegradation of polymers at mesophilic conditions in different environments, and selected studies that used the standards to conduct their biodegradation tests. The method to measure biodegradation, biodegradation requirements to be fulfilled, temperature range to conduct the biodegradation studies and time frame are tabulated.

Standard	Name	Parameter Evaluated	Biodegradation Requirement	Environment	Temperature Range	Time Frame	Selected Published Works
ISO 14852:2018	Determination of the ultimate aerobic biodegradability of plastic materials in an aqueous medium—Method by analysis of evolved carbon dioxide	Measure CO_2_ evolved	>60% for reference material (end of test)	Natural aqueous medium (inoculum from activated sludge, compost, or soil)	20–25 °C(± 1 °C)	6 months	[204,205,206]
ISO 14851:2019	Determination of the ultimate aerobic biodegradability of plastic materials in an aqueous medium—Method by measuring the oxygen demand in a closed respirometer	Measure O_2_ demand	>60% for reference material (end of test)	Natural aqueous medium (inoculum from activated sludge, compost, or soil)	20–25 °C(± 1 °C)	6 months	[127,207,208,209,210,211,212,213]
ISO 17556:2019	Plastics—Determination of the ultimate aerobic biodegradability of plastic materials in soil by measuring the oxygen demand in a respirometer or the amount of carbon dioxide evolved	Measure O_2_ demand, CO_2_ evolved	>60% for reference material (plateau phase or end of test)	Soil	20–28 °C (preferably 25 °C, ± 2 °C)	6 months	[127,214,215,216]
ISO 19679:2019	Plastics—Determination of aerobic biodegradation of non-floating plastic materials in a seawater/sediment interface—Method by analysis of evolved carbon dioxide	Measure CO_2_ evolved	>60% for reference material after 180 days	Seawater/sandy sediment interface	15–25 °C (don’t exceed 28 °C, ± 2 °C)	≤24 months.	[217]
ISO 18830:2016	Plastics—Determination of aerobic biodegradation of non-floating plastic materials in a seawater/sandy sediment interface—Method by measuring the oxygen demand in closed respirometer	Measure O_2_ demand	>60% for reference material (after 180 days)	Seawater/sandy sediment interface	15–25 °C (don’t exceed 28 °C, ± 2 °C)	≤24 months.	
ISO 22403:2020	Plastics—Assessment of the intrinsic biodegradability of materials exposed to marine inocula under mesophilic aerobic laboratory conditions—Test methods and requirements	Measure CO_2_ evolved	≥90% for reference material (within 2 years)	Marine	15–25 °C (don’t exceed 28 °C, ± 2 °C)	24 months.	
ISO 22404:2019	Plastics—Determination of the aerobic biodegradation of non-floating materials exposed to marine sediment—Method by analysis of evolved carbon dioxide	Measure CO_2_ evolved	>60% for reference material (after 180 days)	Marine sediment	15–25 °C (don’t exceed 28 °C, ± 2 °C)	≤24 months.	
ISO 23977-1:2020	Plastics—Determination of the aerobic biodegradation of plastic materials exposed to seawater—Part 1: Method by analysis of evolved carbon dioxide	Measure CO_2_ evolved		Sea water	15–25 °C	≤24 months	
ISO 23977-2:2020	Plastics—Determination of the aerobic biodegradation of plastic materials exposed to seawater—Part 2: Method by measuring the oxygen demand in closed respirometer	Measure O_2_ demand		Sea water	15–25 °C	≤24 months	
EN 17033:2018	Plastics—Biodegradable mulch films for use in agriculture and horticulture—Requirements and test methods	Measure CO_2_ evolved	>90% conversion	Agriculture soil	20–28 °C (25 °C preferred, ± 2 °C)	24 months	[218]
ASTM D5988-18	Standard Test Method forDetermining Aerobic Biodegradation of Plastic Materials in Soil	Measure CO_2_ evolved	>70% for reference material after 180 days (starch or cellulose)	Soil and mature compost	25 ± 2 °C	6 months	[194,214,215,218,219,220,221,222,223,224,225,226,227,228,229,230,231,232,233,234,235,236]
ASTM D6691-17	Standard Test Method forDetermining Aerobic Biodegradation of Plastic Materials in the Marine Environment by a Defined Microbial Consortium or Natural Sea Water Inoculum	Measure CO_2_ evolved	>70% for reference material	Marine (seashore and open ocean). Synthetic seawater with pre-grown population of at least 10 aerobic marine micro-organisms. Natural seawater with inorganic nutrients	30 ± 2 °C	10–90 days	[127,237,238,239]
ASTM D7991-15	Standard Test Method for Determining Aerobic Biodegradation of Plastics Buried in Sandy Marine Sediment under Controlled Laboratory Conditions	Measure CO_2_ evolved	>60% for reference material (after 180 days)	Marine (tidal zone, sandy sediment + seawater)	15–25 °C (do not exceed 28 °C, ± 2 °C)	24 months	[237,240]
ASTM D5929-18	Standard Test Method for Determining Biodegradability of Materials Exposed to Source-Separated Organic Municipal Solid Waste Mesophilic Composting Conditions by Respirometry	Measure O_2_ uptake, Measure CO_2_ evolved	Total O_2_ uptake >80 g Volatile fatty acids > 2 g/kg (invalid test)	Municipal solid waste inoculated with compost	40 ± 2 °C	45 days	
AS 5810-2010	Biodegradable plastics—Biodegradable plastics suitable for home composting	Measure CO_2_ evolved	≥90% (dry weight) degradation of test sample.	Organic waste, kitchen waste	25 ± 5 °C (< 30 °C)	12 months	[241]
NF U52-001:2005	Biodegradable materials for use in agriculture and horticulture—Mulching products—Requirements and test methods	Measure CO_2_ evolved	60% for reference (cellulose) in soil, 90% for cellulose in compost or water media	Soil, compost, and water	28 ± 5 °C	12 months in soil, 6 months in compost, 6 months in water	

CO_2_: Carbon dioxide, O_2_: Oxygen.

**Table 7:** Biotic degradation of polymers at mesophilic conditions measuring CO_2_ evolution or O_2_ demand. Polymer details as shape, initial molecular weight (*M_w_*), and initial crystallinity (*X_c_*); environment in which the biodegradation study is conducted, testing temperature, the extent of biodegradation with the time frame, and the corresponding selected studies are mentioned.

Parameter	Polymer (Shape, Initial *M_w_*, Initial *X_c_*)	Environment	Temperature, °C	Main Result (Test Duration)	Published Studies
CO_2_	Cellulose (powder)	Soil	15, 20, 28	-	[218]
CO_2_	Cellulose (paper mulch)	Soil in laboratory conditions	27	-	[236]
CO_2_	PBS (dumbbell, 21.2 kDa, 57.6%)	Soil compost in laboratory conditions	25 ± 2	65% CO_2_ evolution (180 days)	[225]
CO_2_	PCL (powder, 100 kDa)	Compost in laboratory composting conditions	40	20% mineralization (180 days)	[245]
CO_2_	PLA (films, 100–200 kDa), starch (powder)	Soil in laboratory conditions	28, 40	PLA (100 kDa): 10–40% mineralization (28 °C, 180 days), PLA (200 kDa): 30–95% mineralization (40 °C, 180 days)	[246]
CO_2_	PLA (sheets, 170 and 180 kDa)	Soil inoculated in laboratory conditions	30	5–40% mineralization (60 days)	[221]
CO_2_	PLLA (film, 100 kDa, 30–35%)	Aquatic laboratory conditions	25, 37	PLA (25 ºC): 10% mineralization (180 days), PLA (37 °C): 12% mineralization (180 days)	[247]
CO_2_	PLA (films, 163 kDa)	Soil in laboratory conditions	30	10–25% mineralization (150 days)	[235]
CO_2_	PHB (powder and film), PCL (powder), starch (powder)	Soil in laboratory conditions	22 ± 3	PHB powder: 91% mineralization (90 days), PCL powder: 102% mineralization (270 days), PHB films: 26% mineralization (210 days)	[224]
CO_2_	PHBV (powder, -, 68.9%), cellulose (powder)	Marine in laboratory conditions	25	PHBV: 90% mineralization (450 days)	[240]
CO_2_	PHBV (film), cellulose (powder)	Soil in laboratory conditions	28	PHBV: 90% mineralization (120 days)	[223]
CO_2_	PHB (film), PBSe (film), PBSeT (film)	Marine in laboratory conditions	25	PHB: 70% mineralization (360 days) and 95% mineralization (200 days), PBSe: 95% mineralization (365 and 200 days), PBSeT: 85% mineralization (360 days) and 90% mineralization (200 days)	[217]
CO_2_	PLLA (powder and film, 5, 11, 34, 256 kDa, 0, 18, 42%)	Compost	30, 37	PLA (5 kDa): 70% mineralization (40 days), PLA (11 kDa): 55% mineralization (40 days), PLA (34 kDa): 35% mineralization (40 days), PLA (256 kDa): 20% mineralization (40 days)	[248]
CO_2_	PHA, PBS, cellulose (powder)	Soil in laboratory conditions	25, 37	PHA (25 ºC): 95% mineralization (150 days), PHA (37 ºC): 90% mineralization (180 days), PBS (25 ºC): 90% mineralization (200 days), PBS (37 ºC): 75% mineralization (180 days)	[216]
CO_2_	PU (films)	Soil/Sturm test	30	10 g CO_2_ evolution (30 days)	[249]
CO_2_	PBAT (films, -, 9%)	Soil	25	5% mineralization (100 days)	[250]
CO_2_	PBSe (powder), cellulose (powder)	Soil	28	55–90% mineralization (140 days)	[194]
CO_2_	PHB (film), PBSe (film), PBSeT (film), cellulose (powder)	Soil	25	PHB: 95% mineralization (360 days), PBSe: 90% mineralization (360 days), PBSeT: 90% mineralization (360 days)	[215]
CO_2_	Cellulose (powder)	Soil	25 ± 2	-	[233]
CO_2_	UV irradiated PLA (powder, 198 kDa)	Inoculated sterilized compost, Sturm test	37	PLA (compost): 35–45% mineralization (40 days), PLA (Sturm test): 10–20% mineralization (40 days)	[72]
CO_2_	PHB (powder, 470 kDa))	Sturm test	27	10–80% mineralization (28 days)	[251]
CO_2_	PLA (film, -, 20.8), PHBV (film, -, 72.6), cellulose	Soil	23–25	PLA: 5% mineralization (190 days), PHBV: 25% mineralization (190 days)	[226]
CO_2_	PHA (films), PHB (films)	Soil	23 ± 4	PHA: 0.2 mM/mg CO_2_ (90 days), PHB: 0.3 mM/mg CO_2_ (90 days)	[220]
CO_2_	PHB (film, 175–225 kDa, 48–52%) PHBV (films, 400–300 kDa, 48–52%) with 1% nucleating agent	Microorganisms from marine environment in simulated laboratory conditions	30	PHB: 80–95% mineralization (115 days), PHBV: 90–100% mineralization (115 days)	[238]
CO_2_	PHBV (films, 455 kDa, 47%), cellulose (powder)	Marine (foreshore sand, sand & seawater, seawater) in laboratory conditions	25	PHBV (foreshore sand): 90% mineralization (250 days)	[252]
CO_2_	PHA (film), PLA (bag, bottle)	Marine	30	PHA: 38–45% mineralization (180 days), PLA (bag): 4.5% mineralization (180 days), PLA (bottle): 3.1% mineralization (180 days)	[253]
CO_2_	PHBV (film, 500–600 kDa, 14–58%), cellulose, starch	Soil	25	PHBV: 90% mineralization (250 weeks)	[222]
CO_2_	PHA (film), cellulose (paper)	Soil	20 ± 2	PHA: 70% mineralization (660 days)	[234]
CO_2_	PLA with chain extender (films sheets, 449 kDa, 0.9%), PBAT (films sheet, 44 kDa, 15.2%), cellulose (powder)	Soil in laboratory conditions	28	PLA: 10% mineralization (180 days), PBAT: 20% mineralization (180 days)	[219]
CO_2_	PLA (sheets), PHB (sheets), PBS (sheets), TPS (sheets), PCL (sheets), cellulose (powder)	Soil, home composting *, marine pelagic, and fresh water	25 ± 2, 28 ± 2, 30 ± 1, and 21 ± 1	PLA (soil): negligible (141 days), PLA (home composting): <20% mineralization (365 days), PLA (marine water): <10% relative biodegradation ** (56 days), PLA (fresh water): negligible (56 days), PHB (soil): ≈100% mineralization (136 days), PHB (home composting): <20% mineralization (365 days), PHB (marine water): 90% relative biodegradation (60 days), PHB (fresh water): ≈90% mineralization respect to the reference material (56 days), PBS (soil): negligible, PBS (home composting): <20% mineralization (365 days), PBS (marine water): ≈20% relative biodegradation (56 days), PBS (fresh water): ≈5% relative biodegradation, PCL (soil): ≈90% relative biodegradation (136 days), PCL (home composting): 90% mineralization (200 days), PCL (marine water): ≈80% relative biodegradation (56 days), PCL (fresh water): ≈55% relative biodegradation (56 days),	[127]
CO_2_	PU (films, 48.7 kDa)	Sturm test	35, 30	7.6–8.6 g/L CO_2_	[254,255,256]
CO_2_	PBAT (films)	Soil	30	15% mineralization (120 days)	[257]
CO_2_	PU (films)	Sturm test	35	4.46 g/L CO_2_	[258]
CO_2_	PBSA (films)	Sturm test	37	78% mineralization (40 days)	[259]
CO_2_	PLA (sheets)	Sterilized soil, non-sterilized soil, non-sterilized inoculated soil in laboratory conditions	30	PLA inoculated: 20% mineralization (60 days)	[227]
CO_2_	Cellulose (foil)	Respirometer	20	-	[214]
CO_2_	PBS (sheets, 90 kDa, 58.9%), PEA (sheets, 88 kDa, 40.6%)	Sturm test (activated sludge)	25	PBS: 18% mineralization (40 days), PEA: 12% mineralization (50 days)	[260]
CO_2_	PBSA (films), cellulose (powder)	Compost	25	70% mineralization (55 days)	[241]
CO_2_	PHA (films), PVOH (films)	Sea water	30	PHA: 100% mineralization (100 days), PVOH: 85% mineralization (100 days)	[239]
CO_2_	PCL, PHBV, PBSA, PVOH, PEA, starch, cellulose	Aqueous solution	30	PCL: 26% mineralization, PHBV: 53% mineralization, PBSA: 3% mineralization, PVOH: 5% mineralization, PEA: 36% mineralization (2 weeks)	[204,205]
CO_2_	PLA 3001D (films, -, 7.7%), cellulose (powder)	Aqueous mineral solution (including wastewater)	30	5% mineralization (115 days)	[206]
CO_2_	PBAT (films, 56–38 kDa)	Soil incubation	25	7–15% mineralization (6 weeks)	[148]
CO_2_	PU (foam)	Soil	21 ± 2	43% mineralization (192 days)	[228]
CO_2_	PU (foam), cellulose (paper)	Soil	27 ± 1	10% mineralization (320 days)	[229]
CO_2_	PU (foam)	Sewage water/modified Sturm test	22 ± 2	32–45.6% mineralization (60 days)	[261]
CO_2_	Non-isocyanate polyurethane (NIPU) polyhydroxyurethane (PHU) (film)	Soil	20–28	40% mineralization (120 days)	[230]
O_2_	PCL (powder), cellulose (powder)	Aqueous environment	25	30–35% BOD (150 days)	[207]
O_2_	PHB (film, 735 kDa, 65%), PHBV (film 484 kDa, 46%), PCL (films, 187 kDa, 63%), PES (film, 87 kDa, 61%), PEA (film, 144 kDa, 74%), PBS (film, 79 kDa, 63%), PBA (film, 81 kDa, 70%), PBSe (films, 31.5 kDa, 68%)	Freshwater (river)	25	PHB: 75 ± 16% BOD, PHBV: 76 ± 2% BOD, PCL: 75 ± 8% BOD, PES: 83 ± 2% BOD, PEA: 70 ± 3% BOD, PBS: 3 ± 1% BOD, PBA: 20 ± 4% BOD, PBSe: 6 ± 3% BOD (28 days)	[262,263]
O_2_	PHB (film, 735 kDa, 65%), PHBV (film 484 kDa, 46%), PCL (films, 187 kDa, 63%), PES (film, 87 kDa, 61%), PEA (film, 144 kDa, 74%), PBS (film, 79 kDa, 63%), PBA (film, 81 kDa, 70%)	Freshwater (lake)	25	PHB: 52 ± 7% BOD, PHBV: 71 ± 1% BOD, PCL: 77 ± 1% BOD, PES: 77 ± 1% BOD, PEA: 68 ± 8% BOD, PBS: 12 ± 8% BOD, PBA: 80 ± 13% BOD (28 days)	[262]
O_2_	PHB, PHBV, PCL, PES, PEA, PBS, PBA	Seawater (bay)	25	PHB: 27 ± 10% BOD, PHBV: 84 ± 2% BOD, PCL: 79 ± 2% BOD, PES: 1 ± 1% BOD, PEA: 65 ± 3% BOD, PBS: 1 ± 1% BOD, PBA: 20 ± 2% BOD (28 days)	[262]
O_2_	PHB, PHBV, PCL, PES, PEA, PBS, PBA	Seawater (ocean)	25	PHB: 14 ± 10% BOD, PHBV: 78 ± 5% BOD, PCL: 43 ± 14% BOD, PES: 3 ± 2% BOD, PEA: 46 ± 13% BOD, PBS: 2 ± 0% BOD, PBA: 10 ± 5% BOD (28 days)	[262]
O_2_	Cellulose (filter paper)	Seawater (pelagic, eulittoral, sublittoral, supralittoral, deep sea, buried under sediments)	11–26	-	[212]
O_2_	PLA (film), PBAT (film), PCL (film and powder), cellulose (powder)	Inoculum from activated sludge	30 ± 2	PLA: 3.7% BOD, PBAT: 15.1% BOD, PCL (film): 34.8% BOD, PCL (powder): 37.7% BOD (28 days)	[208]
O_2_	PLA (films, fibers), PHA (films)	Soil	30, 40	PLA (films, 30 ºC, 20 days): 9.8–10.3% BOD, PLA (films, 40 ºC, 10 days): 11.8–17.9% BOD, PLA (fiber, 30 ºC, 20 days): 9% BOD, PLA (fiber, 40 ºC, 10 days): 16% BOD, PHA (films, 30 ºC, 20 days): 26.3% BOD, PHA (films, 40 ºC, 12 days): 49.5% BOD	[264]
O_2_	PBS (sheets), cellulose (powder)	Inoculum from activated sludge	25	PBS: 31% BOD (80 days)	[211]
O_2_	PHBV (powder, 376 kDa, 58.5%), cellulose (powder)	Aqueous conditions	20	PHBV: 80% BOD (28 days)	[213]
O_2_	PLA (film)	Lake water, compost, soil in laboratory conditions	20	PLA (lake water): ≈5 mgO_2_/dm^3^ water, PLA (compost): ≈25 mgO_2_/kg compost, PLA (soil): ≈100 mgO_2_/kg soil (28 days)	[265]
O_2_	PCL (film), PLA (film)	Compost, activated sludge, river water, sea water	20	PCL (compost): 140 mgO_2_/dm^3^, PLA (compost): 125 mgO_2_/dm^3^, PCL (activated sludge): 120 mgO_2_/dm^3^, PLA (activated sludge): 115 mgO_2_/dm^3^, PCL (river water): 10 mgO_2_/dm^3^, PLA (river water): 8 mgO_2_/dm^3^, PCL (sea water): 5 mgO_2_/dm^3^, PLA (sea water): 5 mgO_2_/dm^3^ (7 days)	[266]
O_2_	PBAT (film, 16 kDa)	Mineral medium	25	10% BOD (22 days), 45% BOD (45 days)	[267,268]
O_2_	PHBV (powder, film, undrawn fiber, fivefold-drawn fiber, 250 kDa)	Freshwater, seawater	25	Powder: 18% BOD, film: 18% BOD, undrawn fiber: 18% BOD, fivefold-drawn fiber: 8% BOD (28 days)	[195]
O_2_	PCL (powder), cellulose (powder)	Activated sludge	25	PCL: 20–100% (100 days)	[209]
O_2_	PLA (powder), PCL (powder)	Aqueous conditions	30	PLA: 35% (40 days), PCL: 100% (days)	[210]
O_2_	PLA (film, particle), PBAT (film, particle), PBS (film, particle), PBSA (film, particle), PCL (film, particle), PHB (particle)	Seawater in laboratory conditions	27	PLA: 0.3% BOD, PBAT: 1–1.4% BOD, PBS: 0.1–1.3% BOD, PBSA: 0.4–29.2% BOD, PCL: 14.5–40.9% BOD, PHB: 44–60.4% BOD (4 weeks)	[269]

BOD: biochemical oxygen demand; * using ISO 14855; ** relative to the reference material.

## Data Availability

Not applicable.
